# gSELECT: A novel pre-analysis machine-learning library enabling early hypothesis testing and predictive gene selection in single-cell data

**DOI:** 10.1016/j.csbj.2025.07.047

**Published:** 2025-08-05

**Authors:** Deniz Caliskan, Aylin Caliskan, Thomas Dandekar, Tim Breitenbach

**Affiliations:** Department of Bioinformatics, Biocenter, University of Würzburg, Am Hubland, Würzburg D-97074, Germany

**Keywords:** Gene combinations, Prioritise genes, Machine learning, Dimension reduction, Explainable AI, Generative AI, Transcriptome

## Abstract

Identifying biologically meaningful gene sets and evaluating their ability to separate conditions based on gene expression is an important step in many transcriptomic analyses. While most workflows support data-driven feature selection, few allow direct evaluation of predefined gene sets in a classification context. This limits the ability to assess literature-derived panels or biologically motivated hypotheses prior to downstream analysis. For this, we developed gSELECT, a Python library for evaluating the classification performance of both automatically ranked and user-defined gene sets. It operates on .csv or .h5ad expression matrices with group labels and can be easily integrated into existing analysis pipelines. Gene selection can be based on mutual information ranking, random sampling, or custom input. This supports hypothesis-driven testing without data-derived selection bias and allows direct evaluation of known or candidate markers. Classification is performed using multilayer perceptrons with Monte Carlo cross-validation, either on the full dataset or with a user-defined train/test split. Exhaustive and greedy strategies are available to explore combinatorial effects among genes to identify minimal gene combinations with high predictive power. gSELECT is intended as a pre-analysis tool to evaluate dataset separability and to support early assessment of candidate genes before committing to resource-intensive downstream analyses.

## Graphical Abstract

**Figure:**
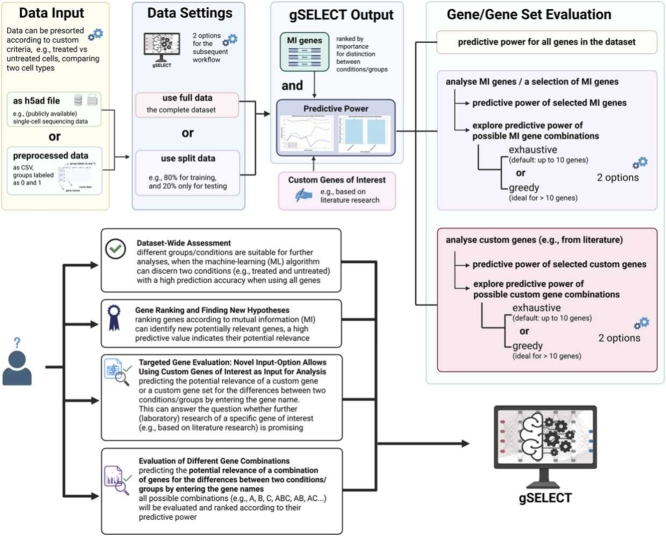

## Data Availability

The analysed lung cancer single cell data is publicly available via the GEO database [16] as GSE137912 and was published by Xue et al. (2020) [15]. Although it contains three tumour models (H358, H2122, and SW1573), which were treated with the KRASG^G12C^ inhibitor ARS-1620 for 0 h, 4 h, 24 h, and 72 h [15], we only analysed the H358 data. The SMART-Seq v4 data analysed for the second scenario was originally published by Yao et al. (2021), who analysed taxonomy of transcriptomic cell types across the isocortex and hippocampal formation in mice [17] and is publicly available via GSE185862. The set of four human pancreatic islet cell datasets produced using different technologies: CelSeq (GSE81076) CelSeq2 (GSE85241), Fluidigm C1 (GSE86469), and SMART-Seq2 (E-MTAB-5061) [18], which was analysed for the third scenario, is available via the R package SeuratData [19]. The adipose tissue data used in the Tutorial was created by Emont et al. (2022) [20] and is available via the Single Cell Portal (https://singlecell.broadinstitute.org/single_cell/study/SCP1376/a-single-cell-atlas-of-human-and-mouse-white-adipose-tissue#study-summary).

## 1 Introduction

**gSELECT is a logical extension of current analysis tools:** Single-cell sequencing has become an essential tool in research, enabling the identification of cellular heterogeneity, drug response and resistance, as well as potential biomarkers [1]. Through single-cell RNA sequencing, researchers can analyse transcriptome heterogeneity, discover distinct cell states and rare cell types [2], and identify specific cell subpopulations [3].

Analytical frameworks in single-cell transcriptomics often rely on clustering, dimensionality reduction methods such as PCA, UMAP, and t-SNE and differential expression analysis to explore cellular heterogeneity. These approaches provide essential insights into transcriptomic variation but may not fully reflect the impact of selected genes [3].

To address this, differential gene expression (DE) analysis is widely used to identify genes associated with specific cellular states, phenotypic differences, or different conditions. In single-cell RNA sequencing (scRNA-seq) data analysis, the term “DE genes” generally refers to genes with statistically significant differences in gene expression compared to others. This comparison can be between different clusters of the same sample or between the same cluster but in different samples, or between cell groups [4]. A variety of methods, which have been compared and discussed in detail by Mou et al. (2020) [5] and by Das et al. (2021) [6], can be applied to find differentially expressed genes in single-cell data [5].

The combination of single-cell measurement techniques with advanced mathematical algorithms has significantly advanced biological research. These algorithms help extract insights from large-scale data, leading to new hypotheses and guiding further experiments to deepen scientific understanding. Examples are automated cell type annotation for single-cell data [7] and tools such as those presented in Breitenbach et al. (2022), Caliskan et al. (2023) and Rasbach et al. (2024) [8], [9], [10]. However, mathematical models rely on specific assumptions about the data and the underlying biological processes. That means results derived from these algorithms may sometimes reflect technical artefacts rather than true biological insights. If subsequent research is based on such artefacts, experiments may fail, leading to unnecessary costs and wasted resources. Furthermore, testing all differentially expressed genes in an experimental setting is almost impossible. As a result, researchers often rely on literature-based target selection, either before or after computational analysis.

AI-based text-mining approaches are increasingly used to support this process. A recently introduced deep-learning-based natural language processing (NLP) model, for instance, can suggest potential target genes based on literature [11]. In the future, these AI-driven approaches are likely to play an increasingly important role in hypothesis generation, providing researchers with candidate genes before any computational or experimental validation. Since such solutions can also fail, e.g. hallucinating potential candidates, further validation may prevent unnecessary work.

However, systematically evaluating such candidate genes within a given single-cell dataset remains a challenge, as existing tools do not provide an intuitive way to integrate external gene lists into exploratory analyses before downstream processing.

Motivated by this need, we developed gSELECT, a pre-analysis framework designed to pre-select genes with high predictive value, and allowing downstream analyses to focus on the most informative candidates.

**Specific abilities of gSELECT:** gSELECT is designed as a complementary tool to enhance already existing workflows by offering a machine-learning-driven perspective on gene selection and assessing the predictive power of certain genes of interest. Rather than replacing DE methods or standard exploratory analyses, gSELECT integrates into existing pipelines by prioritising genes based on their predictive relevance before or after traditional analyses. The key concept is that the highest predictive power is associated with carrying the most information for separating the observed differences. Consequently, these genes might be promising candidates for further causal hypotheses and model building in the investigated research issue.

The gSELECT library requires only an h5ad file as input, or a CSV file, and integrates multiple analytical methods based on different mathematical principles, such as Mutual Information (MI)-based gene ranking with a Machine Learning (ML) model to assess the predictive power of genes while minimising programming effort. Mutual information, a concept explained in detail in Zeng (2015) [12], can be used to quantify the dependence of two random variables [13]. A higher mutual information value represents a higher predictability [14]. The concept of MI genes is also employed by the principal feature analysis [8], [9], which this work builds upon. Accordingly, we use MI-based gene ranking for identifying genes with high discriminative power.

The resulting ranked MI genes are then evaluated using a multilayer perceptron (MLP) classifier, which is trained to assess their predictive strength. While standard approaches such as data visualisation and differential expression analysis remain essential, gSELECT can give an additional perspective based on the predictive power of (a small number of) selected genes.

The rationale behind this approach is a dual strategy that if one method identifies a promising result, yet another method contradicts it, the findings should be critically reassessed. By evaluating the predictive power of certain genes before or after downstream analysis, gSELECT can give an additional computational perspective to reduce false discoveries and guide hypothesis generation.

A key novel feature of gSELECT is its ability to also assess user-defined gene sets, whether derived from literature, hypothesis-driven selection, or AI-based predictions. To our knowledge, there is currently no existing tool that allows for the direct evaluation of the predictive power of custom selected genes in single-cell datasets without needing pre-selection through a downstream analysis. With gSELECT, it is possible to input custom genes of interest and evaluate their relevance for the given dataset. Additionally, gSELECT can evaluate custom gene combinations by systematically testing all possible combinations of the selected genes and identifying sets with the highest predictive power in distinguishing between conditions.

## 2 Methods

To demonstrate the features of gSELECT, we analyse single-cell data published by Xue et al. (2020), who examined KRAS^G12C^ mutant cell populations upon ARS-1620 treatment [15]. Their single-cell RNA sequencing data, which is publicly available via the GEO database [16] as GSE137912, contains three tumour models (H358, H2122, and SW1573) which were treated with the KRAS^G12C^ inhibitor ARS-1620 for 0 h, 4 h, 24 h, and 72 h [15]. A detailed case study using gSELECT to analyse this lung cancer cell line data and various standard methods to verify the results can be found in the Supplement. Additionally, to showcase the versatility of gSELECT, we analyse SMART-Seq v4 data (GSE185862), which was published by Yao et al. (2021), who analysed taxonomy of transcriptomic cell types across the isocortex and hippocampal formation in mice [17], and a set of four human pancreatic islet cell datasets produced using different technologies: CelSeq (GSE81076) CelSeq2 (GSE85241), Fluidigm C1 (GSE86469), and SMART-Seq2 (E-MTAB-5061) [18], which is available as “panc8” via the R package SeuratData [19] (version 0.2.2.9002, available at https://github.com/satijalab/seurat-data). Additionally, we provide a detailed Tutorial demonstrating all functions of gSELECT using a subset of the single cell atlas of human and mouse white adipose tissue by Emont et al. (2022) [20] in the Supplement.

All steps were executed in a structured and modular pipeline, ensuring full reproducibility. The code used for preprocessing, quality control, and analysis is available in the Supplement. To streamline the Methods section, we will focus on gSELECT methods here, all relevant preparation steps required for gSELECT are described in detail in the Supplement and in the gSELECT Tutorial in the Supplement.

### 2.1 gSELECT

We developed gSELECT, a Python-based tool for evaluating gene subset performance in classification tasks. gSELECT supports multiple gene selection strategies, including ranking genes by mutual information (MI), using all non-constant genes, and drawing random subsets for comparison. One of the main features of the tool is its flexibility: Users can bypass automated ranking entirely and evaluate custom, user-defined gene sets, allowing direct assessment of biologically motivated candidates or literature-derived panels.

Once expression data is pre-processed from .csv or .h5ad files (see Supplement), gSELECT performs classification using a multilayer perceptron (MLP) with repeated training and validation sweeps. The tool also supports exploratory search methods such as exhaustive or greedy subset evaluation, enabling the discovery of compact gene panels with high predictive power.

The full implementation is available on GitHub (https://github.com/CaliskanDeniz/gSELECT). Further methodological details are provided in the sections below.

#### 2.1.1 Implementation and design

We implemented gSELECT as a Python library, designed with a clear separation between four functional layers: data handling, feature selection, classification, and visualisation. This design enables the tool to be extensible and easily adaptable to different workflows. Each module was designed to operate independently.

**Fig. 1:**
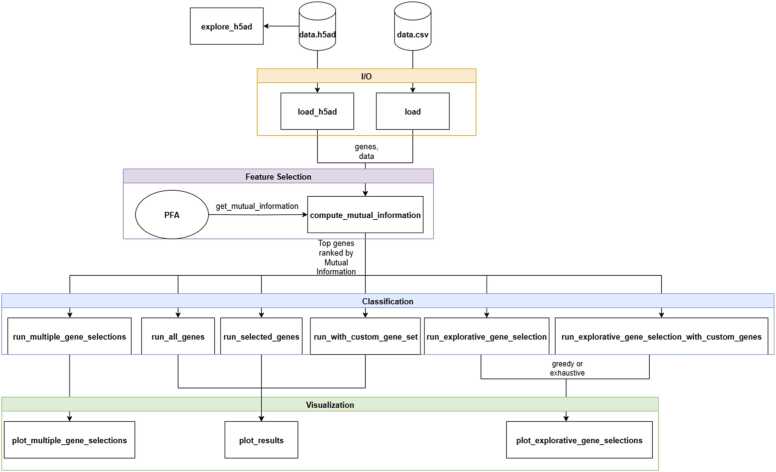
Overall system architecture of gSELECT.

Internally, gSELECT follows a function-oriented design pattern, allowing users to build reproducible and customizable pipelines and integrate the library into their own pipelines. For instance, the classification logic is designed to remain entirely independent of the feature selection process, which makes it easy to swap out selection methods or experiment with different classifier types without disrupting the rest of the pipeline. The tool works with both .csv and .h5ad files as input, to make it compatible with standard data formats in transcriptomics workflows. Multithreading is used for computationally intensive tasks such as explorative gene selection. A schematic overview of the system is provided in the UML diagram in Fig. 1.

#### 2.1.2 Data handling and preprocessing

gSELECT supports input from both .h5ad files, which are commonly used in AnnData-based single-cell analyses, and standard .csv files, ensuring compatibility with a wide range of workflows. Expression data is loaded into memory as Pandas DataFrames, with built-in support for sparse matrices to optimize memory usage during large-scale analyses.

For .h5ad input, the load_h5ad() function reads the dataset using Scanpy[21], filters cells based on a specified metadata column (e.g. cell type or condition), and encodes class labels for classification. The returned expression matrix includes both the label row and gene expression values, properly transposed for downstream use. Gene names are also returned as a separate DataFrame for reference.

The exploratory utility explore_h5ad() allows users to preview key aspects of the dataset, including metadata structure, unique values in annotations, and matrix sparsity, providing a quick diagnostic before analysis begins.

For .csv input, the load() function uses Polars [22] for efficient parsing. Further information on the functions is provided in Table 1.

**Table 1:** Overview of gSELECT functions for data handling and processing.

**Function Name**	**Purpose**	**Inputs**	**Outputs**	**Notes**
explore_h5ad()	Provides summary and metadata diagnostics from an .h5ad file.	file_path (str)	None (prints output to console)	Prints shape, metadata contents, sparsity info, and unique values in annotations
load_h5ad()	Loads and filters single-cell expression data from .h5ad, returning gene names and label-encoded expression matrix.	file_path, filter_column, filter_values	Tuple: (gene_names_df, expression_df)	Encodes labels as 0/1 and transposes data for classification; supports sparse matrices
load()	Loads a gene expression matrix from a .csv file using Polars, with optional threading and memory optimization.	path, n_threads, use_low_memory	Tuple: (gene_names_df, expression_df)	Fast CSV parser for large bulk datasets; returns Pandas DataFrames

#### 2.1.3 Feature selection

Gene selection is performed using mutual information as implemented in Principal Feature Analysis (PFA) and explained in detail in the respective publications by Breitenbach et al. (2022) [8] and Caliskan et al. (2023) [9]. Rather than implementing the calculation of mutual information within the framework, gSELECT relies on established methods from the PFA approach. The main interface, compute_mutual_information(), ranks genes by their MI scores relative to class labels (e.g. cell types or conditions). By default, gSELECT adapts this bin size based on dataset size to avoid both over-discretization and unnecessary computational overhead. gSELECT adjusts the number of bins following Sturges’ rule (1 + log₂(n), where n is the number of samples). This method offers a data-driven discretisation. Each bin divides the gene expression range into equal intervals. Mutual information is calculated only when every bin contains a minimum number of samples (10 by default). This helps to prevent inflated or unstable MI scores that may happen when bins have few samples, which can occur for genes with highly skewed or zero-inflated expression distributions. The minimum number of samples per bin can be changed if needed to fit specific dataset properties or analysis goals. While this method usually works well across various sample sizes, the number of bins is intentionally kept low to ensure enough support in each bin. In cases where expression values are highly skewed or sparse, as is common for some genes in single-cell data, this may limit resolution but increases robustness. If a different discretisation is desired, for example to increase resolution in specific use cases, the number of bins can be set manually as part of the configuration.

Importantly, MI-based selection is not mandatory. If a user provides a custom list of genes (e.g. from prior knowledge or literature), gSELECT will bypass MI calculation and assign mock scores to validate and encode those genes for classification. This enables evaluation of user-defined panels alongside automatically ranked genes without altering core workflows. Mutual information results are automatically sorted and can be cached as CSV files for reproducibility. Genes specified in an exclusion list can be filtered post hoc from the ranked output. The resulting ranked table can be passed directly to downstream classification or exploratory search functions. The functions are summarised in Table 2.

**Table 2:** Overview of gSELECT functions for feature selection.

**Function Name**	**Purpose**	**Inputs**	**Outputs**	**Notes**
compute_ mutual_ information()	Main interface for computing or loading mutual information (MI) scores. Supports saving and filtering.	gene_names, expression_data, gene_list, exclusion_list, top_mutual_information, output_folder	DataFrame of ranked genes with MI scores	Loads cached MI file if it exists; filters excluded genes
get_mutual_ information()	Computes mutual information scores from scratch or mocks scores for a custom gene list.	gene_names, expression_data, gene_list, top_mutual_information, min_datapoints, basis_log	DataFrame with gene_name, index feature, and mutual information	Uses Principal Feature Analysis (PFA); mock MI = 1 for user-defined genes

#### 2.1.4 Classification and exploratory gene selection

Classification in gSELECT is built around a supervised learning pipeline using the MLPClassifier from *scikit-learn* [23]. Gene expression data are first min-max normalized to ensure numerical stability. The MLP model is trained using the Adam optimizer with a configurable number of iterations (default: 500).

To estimate classification performance under variability of the available data, gSELECT uses Monte Carlo cross-validation (MCCV): classification is repeated across randomly shuffled train/test splits. The number of iterations, specified by number_sweeps (default = 10), can be adjusted to allow for a trade-off between computational load and statistical precision. The classification core, run_gene_classification(), supports multiple selection modes: (i) user-defined genes, (ii) randomly sampled sets of genes of equivalent size, and (iii) all genes with a non-constant expression pattern (non-constant genes). Results from each sweep include test/train balanced accuracy and misclassification counts. Higher-level wrappers like run_selected_genes() and run_with_custom_gene_set() simplify benchmarking across gene selection strategies.

For performance analysis across panel sizes, run_multiple_gene_selections() evaluates classifier performance using incrementally larger subsets (e.g. top 1 to *n* genes), revealing trade-offs between model size and accuracy.

To support exploratory gene discovery, gSELECT includes an exhaustive subset evaluator via run_explorative_gene_selections(). This function scores all possible non-empty subsets of the top-*n* MI-ranked genes. For large *n*, where complete enumeration becomes infeasible, the tool defaults to a greedy forward selection strategy (run_greedy_selection()), with optional enhancements like beam search and swap-based optimization. The functions are summarised in Table 3.

**Table 3:** Overview of gSELECT functions for classification and exploratory gene selection.

**Function Name**	**Purpose**	**Inputs**	**Key Parameters**	**Output**
run_gene_classification	Core engine that performs repeated MLP classification on a selected gene set.	expression_data, selected_gene_indices, gene_selection, test_data	number_sweeps, max_iterations	Test/train accuracy, misclassifications, mode ID
run_selected_genes	Evaluates top-n MI-ranked genes, optionally with random baseline.	expression_data, gene_mutual_information, test_data	top_n_genes, include_random	List of classification result tuples
run_with_custom_gene_set	Evaluates a user-defined gene panel, optionally with random baseline.	expression_data, selected_gene_names, gene_mutual_information, test_data	number_sweeps, include_random	List of classification result tuples
run_all_genes	Trains classifier using all non-constant genes.	expression_data, gene_mutual_information, test_data	number_sweeps, max_iterations	List containing classification result tuple
run_multiple_gene_selections	Benchmarks classification across multiple gene panel sizes.	expression_data, gene_mutual_information, test_data	gene_selection (list of sizes)	Dict mapping panel size → result list
run_explorative_gene_selections	Performs exhaustive or greedy subset search over top-n MI genes.	expression_data, gene_mutual_information, test_data	top_n_genes, greedy_threshold, beam_width, allow_swaps	Dict mapping gene subset → result list
run_greedy_selection	Greedy forward selection with optional swaps and beam search.	expression_data, gene_mutual_information, test_data	max_panel_size, allow_swaps, beam_width	Dict mapping gene subset → result list
run_explorative_gene_selections_with_custom_set	Exhaustive subset evaluation of a custom user-defined gene list.	expression_data, selected_gene_names, gene_mutual_information, test_data	num_threads, number_sweeps, max_iterations	Dict mapping gene subset → result list

Both exploratory modes are parallelized using Python’s ThreadPoolExecutor for efficient execution in multicore systems. All classification outputs include per-sweep results for downstream visualisation and analysis. Greedy and exploratory searches can both be performed with custom gene sets.

#### 2.1.5 Visualisation and Interpretation of Results

To evaluate classification performance and gene selection efficacy, gSELECT includes a visualisation module that summarizes model behaviour across different experimental conditions.

The results are visualised in a series of figures that support interpretation at multiple levels:

1. Per-strategy accuracy curves
  A line plot shows mean test and train accuracy across sweeps, with standard deviation bands. This allows users to assess the consistency and generalization of each gene selection strategy, including mutual information ranking, random gene sets, and full feature inclusion.
2. Misclassification summary
  A horizontal bar chart reports the average number of misclassified samples per strategy, with error bars reflecting variation across sweeps. This provides an interpretable summary of absolute error, independent of accuracy percentages.
3. Gene panel size effects
  gSELECT generates comparative plots across multiple selection sizes. Accuracy and misclassification trends are shown as functions of the number of selected genes, helping users identify minimal gene sets that retain high performance.
4. Exploratory subset ranking
  For in-depth analysis, gSELECT ranks gene subsets by classification accuracy based on exhaustive or greedy exploration. Top-performing combinations are shown in a ranked bar chart, annotated with mean ± standard deviation, and accompanied by a legend mapping shorthand labels (e.g. S1, S2) to gene identifiers.
  All figures are exported at 600dpi resolution and are also exported to CSV files containing statistics. The functions are summarised in Table 4.

**Table 4:** Overview of gSELECT functions for visualisation and interpretation of results.

**Function Name**	**Purpose**	**Input(s)**	**Output(s)**	**Notes**
plot_results()	Summarizes classification strategies across sweeps using line and bar plots.	results (list of 4-tuples), output_folder, save_csv, save_png	Balanced accuracy plot, misclassification bar chart, CSV summary	Pools replicates; supports multiple gene selection modes
plot_multiple_gene_selections()	Compares model accuracy and misclassification as a function of gene panel size.	results (dict of panel size → 4-tuples), output_folder	Line plot (accuracy), bar chart (errors), CSV summary	Useful for finding optimal gene panel sizes
plot_explorative_gene_selections()	Ranks and visualises top-performing gene subsets (exhaustive or greedy).	results (dict of subset → list of runs), top_n, output_folder	Bar chart with gene subset legend, CSV of ranked subsets	Optionally shows delta accuracy; adapts layout to gene label length

## 2.1.6 Computational performance considerations

The computational demands of gene selection in gSELECT vary depending on the strategy employed, particularly in the context of exploratory subset evaluation. By default, the function run_explorative_gene_selections() performs an exhaustive search over all non-empty subsets of the top *n* genes ranked by mutual information. This search is combinatorial in nature:

• 5 genes → 31 subsets
• 10 genes → 1023 subsets
• 15 genes → ∼32,000 subsets
• 20 genes → > 1 million subsets

While this strategy offers a complete landscape of subset performance, its exponential scaling quickly becomes impractical as *n* increases.

To mitigate this, gSELECT incorporates a greedy forward selection engine that automatically activates when the number of candidate genes exceeds a threshold (default: 10). In greedy mode, the algorithm incrementally assembles the best-performing panel by adding one gene at a time, based on performance gains.

Two advanced features are optionally available in greedy mode:

• **Backtracking (with replacement)**: After each gene is added, the algorithm may optionally attempt to *swap* one selected gene with an unselected one to see if it improves performance. This allows the method to escape early suboptimal decisions and refine the panel further.
• **Beam search**: Rather than following a single best path, the beam search maintains multiple top-performing partial paths at each step (the “beam”). This increases the chance of discovering better solutions while keeping the search space tractable.

Both features are disabled by default to keep runtime short and interpretation simple, but they can be enabled for more thorough panel exploration when computational budget permits.

Regardless of strategy, all classification tasks are parallelized using Python’s ThreadPoolExecutor, with the number of worker threads determined dynamically based on available CPU cores. Gene expression data is internally handled in dense format, even if loaded from sparse inputs. This simplifies computation but can increase memory usage for large-scale single-cell datasets. In sum, gSELECT balances exhaustive accuracy evaluation with scalable heuristics and parallel execution, making it suitable for a wide range of gene selection tasks, from small biomarker panels to high-dimensional screens.

## 2.2 gSELECT visualisations

For the analyses and visualisations, gSELECT was employed to compute mutual information (MI) for feature selection, ranking genes based on their information content. Explorative gene selection was applied using multiple sweeps to identify biologically relevant genes. The standard analysis methods to validate the MI genes and the standard methods to create the respective visualisations are described in detail in the Supplement.

## 3 Results

gSELECT is a pre-analysis Python library that integrates easily into any existing single-cell analysis workflows using h5ad or csv files. It complements standard methods by offering a method to assess the predictive power of gene expression differences between user-defined groups (e.g. treated vs. untreated cells or other phenotypic differences). Using mutual information (MI), gSELECT ranks genes, referred to as MI genes, based on how informative they are for distinguishing between groups. Ideally, a small number of top-ranked MI genes is sufficient to correctly classify single cells (here referred to as samples), highlighting their relevance for the observed differences.

In addition to MI-based ranking, gSELECT allows evaluation of user-defined gene sets, for example derived from literature, prior hypotheses, or results from other analyses such as differential expression testing (e.g. Wilcoxon test or DESeq2, which are for example available via Seurat’s [24], [25], [26], [27], [28] FindMarkers function, or Scanpy’s [21] rank_genes_groups) or logistic regression. It enables assessing the predictive power of these genes, both individually and in combination, to explore smaller gene panels while evaluating classification performance. For context, predictive performance can also be compared to that achieved with randomly selected genes or with models trained on all available genes. These functions can support interpretation alongside common visualisation approaches.

Visualisation methods such as UMAP [29] or t-SNE [30] can reveal different levels of separation between groups. These considerations are important when deciding how to proceed with further analysis. Depending on the data, different scenarios can occur: (1) no clear separation and little signal, (2) no clear separation but informative differences, (3) or clear separation between groups. These three scenarios are examined in this context. In the first, we use a synthetic dataset based on H358 lung cancer cells with only minimal artificially introduced variation, representing a scenario with no meaningful signal, which means that the gene expression set seems to contain no information about the desired investigated differences modelled by the labels. The second examines sex differences in mouse cortex SMART-Seq data, where UMAP shows no clear separation, but predictive information is present. The third scenario analyses two human pancreatic islet cell types, which display clear differences and separation in the UMAP visualisation.

To provide comprehensive context, the Supplementary Material includes both a practical tutorial (demonstrating core functions with adipose tissue single-cell data from Emont et al., (2022) [20]) and an in-depth case study applying the full gSELECT workflow to a dataset of lung cancer cells undergoing ARS-1620 treatment, which was published by Xue et al. (2020) [15]. These materials show the broader range of analyses possible with gSELECT. The case study illustrates gene ranking and selection, predictive power evaluation across different conditions and preprocessing choices, comparison with established methods (e.g. DESeq2 [60], Wilcoxon, MAST [61]), enrichment and network analyses, survival analysis, and evaluation of custom gene panels informed by literature. It also includes additional benchmarking analyses comparing gSELECT-derived gene rankings with results from multiple standard approaches in R and Python (e.g. Seurat, Scanpy [24], [25], [26], [27], [28], scClassify [31], scanpy [21], and AUROC-based scoring), providing further validation and context for interpreting MI-based gene selection. While these extended analyses are detailed in the Supplement, here we focus on three concise scenarios to illustrate gSELECT’s core functions and its intended use in pre-analysis. These examples were selected to cover different species, sequencing technologies, and file formats commonly used in single-cell workflows. A detailed description of how to use the respective functions mentioned in the following analyses is available in the Tutorial provided in the Supplementary Data. The tool will be available as Python library and via GitHub (https://github.com/CaliskanDeniz/gSELECT), and can be integrated in any pipeline or analysis workflow.

### 3.1 First scenario: No differences between the two groups

If cells are treated with a certain drug that does not affect them, treated and untreated cells will typically show minimal differences in gene expression. To simulate this scenario, we created a synthetic dataset based on the data of the untreated H358 cells, published by Xue et al. (2020), who investigated gene expression after treatment with the KRAS^G12C^ inhibitor ARS-1620 in human tumour cell models [15].

**Fig. 2:**
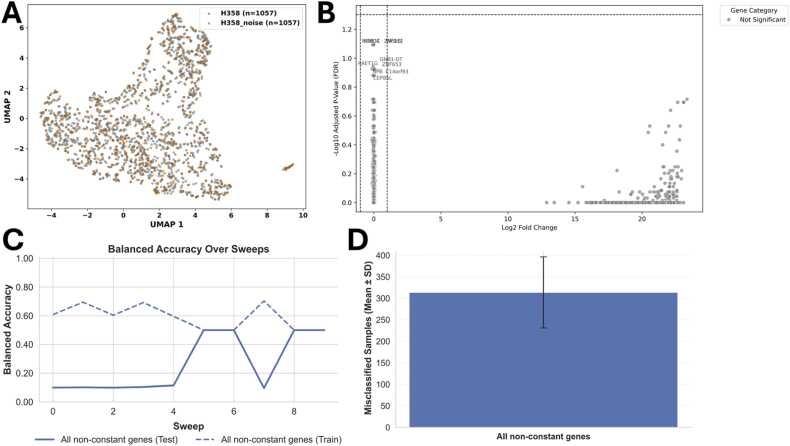
Results for the analysis of two (very) similar groups, with 1 % noise (scenario 1).**(A)** UMAP visualisation of the two conditions. **(B)** Volcano plot visualising the differentially expressed genes according to Wilcoxon analysis. **(C)** Balanced Accuracy across sweeps for all available non-constant genes. **(D)** Number of misclassified samples for all non-constant genes (a mean of 313.5 misclassified samples (with a standard deviation of 87.03) out of a total number of 2114 cells (here referred to as samples, 1057 cells for each group)).

One group contained the original data, while the other group was generated by introducing only 1 % Gaussian noise, resulting in two very similar groups, as also seen in the UMAP visualisation in Fig. 2A. Accordingly, Wilcoxon analysis (via scanpy’s rank_genes_groups [21]) could not identify significant genes between the two groups, and a volcano plot confirmed the absence of clearly differentially expressed features (Fig. 2B).

We then used gSELECT to assess the predictive power of all available non-constant genes in this dataset (as recommended as an initial step in the Tutorial). The balanced accuracy of approximately 58 % in training (dashed line) and 26 % in test (solid line) indicates that the model cannot reliably distinguish between the two groups (Fig. 2C). This is also reflected in the high number of misclassified cells (Fig. 2D), with a mean of 313.5 misclassifications (standard deviation of 87.03) out of 2114 cells (1057 per group). Such results suggest a high degree of similarity between the groups, indicating that further analyses (e.g. pseudobulk approaches or targeted validation experiments) may not be warranted. This illustrates how gSELECT can serve as an early assessment step to evaluate whether a sufficient predictive signal is present to justify more detailed downstream analyses.

However, there is a possibility that the selected model may not accurately capture the functional relationships between gene expression and the observed differences or the cell fate. Furthermore, the training of the models can fail, e.g., due to a unfortunate hyperparameter setting. Therefore, performing a second analysis step, such as Wilcoxon analysis or considering the top-ranked MI genes, might help to avoid false negatives and “overlooking” potentially relevant genes. Here, calculating the balanced accuracy for custom genes (e.g. the top-ranked MI genes or relevant genes identified by other analysis methods), which is one of the novel features of gSELECT and is described in detail below and in the Tutorial in the Supplementary Data, can be used for additional validation.

### 3.2 Second scenario: Two rather similar groups according to their UMAP-visualisation

Another possible scenario in single-cell analysis is that a comparison between two groups shows no clear separation between the two groups in a UMAP visualisation. This could indicate a rather high similarity between the two groups and might prompt the question whether additional investigations are justified.

For this scenario and to assess the generalisability of our approach across technologies and species, we used the gSELECT library to analyse a publicly available Smart-Seq v4 single-cell dataset of mouse cortex and hippocampal formation (GSE185862) [17]. This dataset includes high-depth, full-length transcriptome profiles of ∼73,000 cells isolated from adult mouse brain tissue. We filtered for cells annotated as male (M) or female (F) based on donor sex metadata and treated sex as the prediction target.

#### 3.2.1 Evaluating predictive power despite overlapping UMAP clusters

gSELECT can be used to evaluate the presence of discriminative information in a dataset, supporting decisions about subsequent analyses through MI gene ranking and classification performance assessment. The resulting classification performance can be summarised using balanced accuracy, which indicates how well the model separates the groups. A high balanced accuracy would suggest that the ML algorithm is able to distinguish the groups using a certain number of MI genes, ideally consistently across multiple sweeps.

**Fig. 3:**
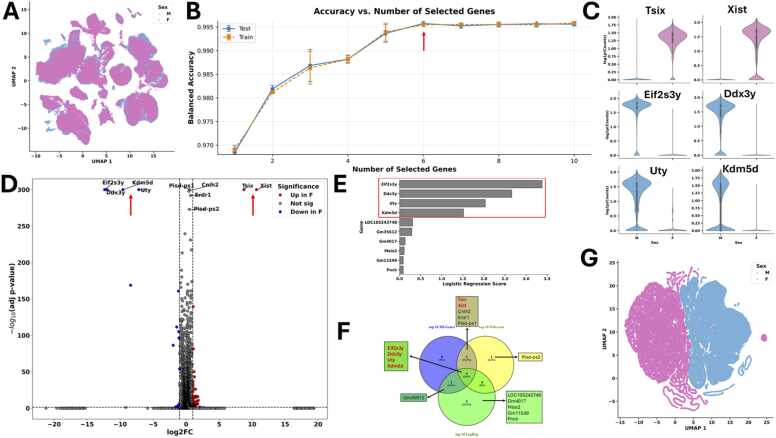
Analysis of sex-based differences in mouse cortex single-cell data (GSE185862).Smart-Seq v4 single-cell RNA-seq data from adult mouse cortex and hippocampal formation (∼73,000 cells) was used to test gSELECT in a cross-species, high-depth setting with donor sex (male vs. female) as classification target. **(A)** UMAP embedding computed from all expressed genes shows broad overlap between male (M) and female (F) cells, with no clear separation into distinct clusters, indicating similarity across sexes at the global level. **(B)** gSELECT analysis with Mutual information (MI) and Machine Learning. Balanced accuracy improves with the number of top MI-ranked genes included, reaching ∼99.5 % with the top 6 genes, indicating that these few markers capture nearly all discriminative signal for sex classification. **(C)** Violin plots of the top 6 MI-selected genes highlight known sex-specific expression patterns: Xist and Tsix (X-chromosome inactivation markers) are enriched in female cells, while Eif2s3y, Ddx3y, Uty, and Kdm5d are Y-linked genes predominantly expressed in male cells. **(D)** Volcano plot of differential expression (based on the results of a Wilcoxon rank sum test via scanpy’s rank_genes_groups() function) comparing male and female cells identifies the same sex-specific markers among the most significant genes, supporting the robustness of MI-based selection. **(E)** Logistic regression feature importance ranks also prioritise the same Y-linked markers among top features. **(F)** Venn diagram comparing the top 10 markers selected by MI ranking, differential expression, and regression feature importance highlights overlap among methods. **(G)** UMAP embedding computed using only the top 6 MI-selected genes shows clear clustering between male and female cells.

In the UMAP projection computed from all expressed genes, male and female cells show a broad overlap with no clear clustering by sex (Fig. 3A). This pattern is consistent with the expectation that most gene expression is shared across sexes, and that sex-specific differences may be relatively subtle.

Applying gSELECT to this dataset helps with systematic investigation of these subtle differences. By ranking genes via mutual information, we observed that balanced accuracy increased as more top-ranked genes were included, reaching ∼99.5 % with only six genes (Fig. 3B). This demonstrates that despite the lack of visible separation in the global UMAP, these genes together capture nearly all the discriminative signal needed to distinguish male from female cells.

Examination of the top six MI-ranked genes confirms their biological plausibility: four (*Eif2s3y*, *Ddx3y*, *Uty*, *Kdm5d*) are Y-chromosome-linked and expressed predominantly in male cells [32], [33]. At the same time, Xist and Tsix are involved in X-chromosome inactivation and enriched in female cells [33], [34] (Fig. 3C). Differential expression testing and logistic regression feature importance analyses using scanpy’s rank_genes_groups() function similarly prioritise these markers (Fig. 3D and E), and a Venn diagram (Fig. 3F) shows robust overlap among selection methods.

Notably, when we recomputed UMAP using only these six genes, the separation between male and female cells became more apparent (Fig. 3G). The reason is that UMAP has no information about the phenotypic difference of interest and separates cells according to minimising its objective to respect the neighbouring of cells of the high dimensional space also in the low dimensional space. Consequently, phenotypic differences do not necessarily coincide with the clustering in the low dimensional space. However, in the space of genes that are most associated to the phenotype of interest (in this case six genes), neighbouring of cells is more associated with the phenotypic difference of interest which is thus reflected in the 2-dimensional projection as well. In other words, gSELECT can infuse specific information about the research objective into methods, such as UMAP, and thus make them more powerful.

#### 3.2.2 Using gSELECT to evaluate remaining signal after excluding known markers

To further demonstrate the use of gSELECT as a pre-analysis tool, we tested how excluding known marker genes influences classification performance. This type of analysis helps evaluate whether additional genes in the dataset contain discriminative information once well-established features are removed.

For this test, we excluded the six top-ranked sex-specific genes identified in the previous analysis (*Tsix, Xist, Eif2s3y, Ddx3y, Uty, Kdm5d*). The goal was to determine whether the remaining genes are sufficient to separate male and female cells.

**Fig. 4:**
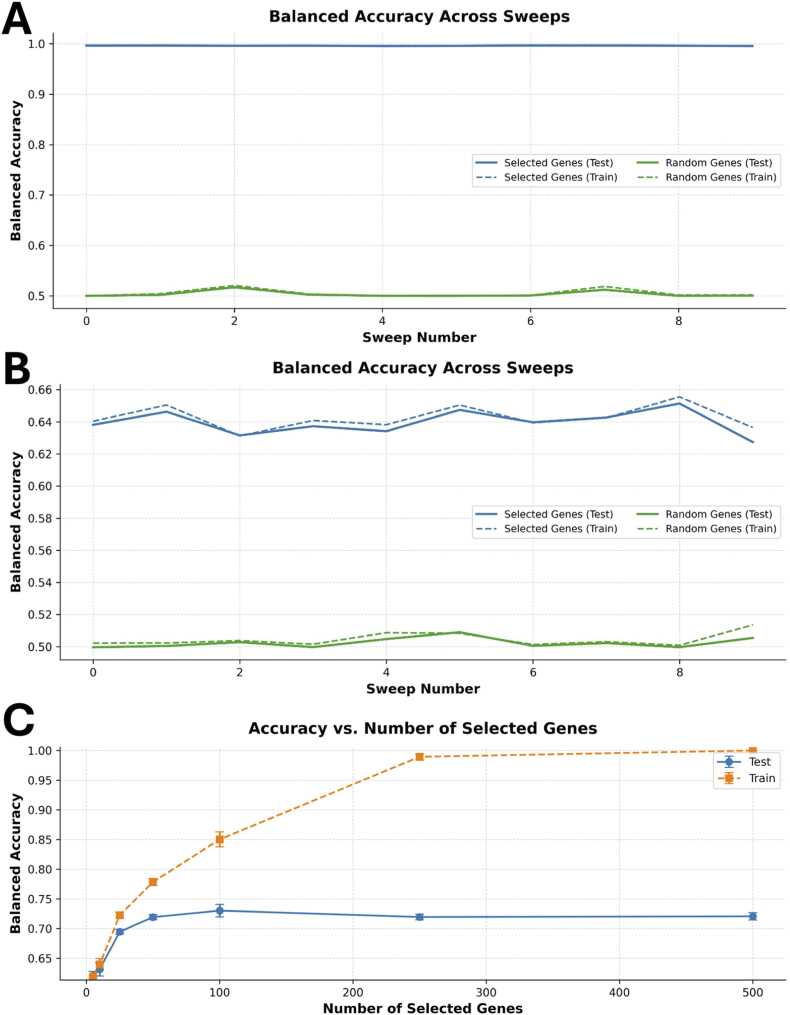
Classification performance after exclusion of known marker genes. **(A)** Balanced accuracy across 10 Monte Carlo cross-validation sweeps using the top 10 mutual information (MI)-ranked genes, including known sex-specific markers (*Tsix*, *Xist*, *Eif2s3y*, *Ddx3y*, *Uty*, *Kdm5d*). Test accuracy remains close to 100 %, indicating strong separability between male and female cells. **(B)** After excluding the six marker genes, classification using the next top 10 MI-ranked genes results in test accuracy near random (∼56 %), suggesting that the majority of predictive signal was contained in the excluded genes. **(C)** Balanced accuracy as a function of the number of selected genes, based on the MI ranking excluding known markers. Test accuracy increases up to ∼25 genes, then plateaus, while training accuracy continues to rise, indicating potential overfitting.

Fig. 4A shows classification performance using the ten top-ranked MI genes, including known sex-specific markers. In this setting, balanced accuracy was ∼99.5 %. After removing the six sex-specific genes from the ranking, we repeated the classification using the top 10 remaining genes (Fig. 4B). Balanced accuracy dropped to ∼56 %, close to random performance. This confirms that the excluded genes account for most of the discriminative signal with regard to sex of the mice in this dataset.

We next evaluated how classification performance varies with the number of selected genes. Fig. 4C shows the relationship between gene panel size and balanced accuracy on training and test data. Performance increased steeply up to around 25 genes, after which test accuracy plateaued around 72 %. In contrast, training accuracy continued to rise and eventually approached 100 %.

This divergence could indicate possible overfitting, where the model learns dataset-specific patterns that do not generalise to unseen data. The results suggest that most of the discriminative information is captured by the top ∼25 genes, while additional features facilitate overfitting. These findings support the use of gSELECT to identify minimal gene panels that seem to have some predictive power and could be analysed further while avoiding model overfitting.

### 3.3 Third scenario: Two clearly different groups

In some datasets, distinct biological groups can be distinguished based on global gene expression patterns. To illustrate gSELECT’s behaviour under such conditions, we analysed the “panc8” dataset from the SeuratData R package [19] (version 0.2.2.9002), which includes single-cell RNA-seq data from four human pancreatic islet cell studies generated using different technologies: CelSeq (GSE81076) CelSeq2 (GSE85241), Fluidigm C1 (GSE86469), and SMART-Seq2 (E-MTAB-5061) [18]. The dataset is commonly used in Seurat tutorials, for instance in workflows demonstrating data integration (https://satijalab.org/seurat/articles/integration_mapping.html). gSELECT supports comparisons between any groups defined by the user, whether cell types, conditions, or metadata annotations, making it flexible for a wide range of questions. It can be used on raw counts, normalized data, or HVG-filtered matrices, with or without batch correction, depending on the specific question. To assess how batch effects and integration impact classification, we applied gSELECT to both the unintegrated and integrated versions of the dataset. For both versions (integrated and non-integrated), we subset the dataset by annotated cell types, selecting alpha (labelled as 0) and beta cells (labelled as 1) as the two cell types for the analysis, and prepared the CSV file as described in the Supplement (please see Supplementary Method Descriptions and Additional Validation of gSELECT with Different Datasets for details on dataset preparation). The analysis of the uncorrected data can also be found in the Supplement.

For the results of the integrated data, which are displayed in the following, we proceeded with the integrated data for gSELECT analysis. The expression matrix was prepared in R and exported to.csv format. As described in the Supplementary Methods section, alpha cells were labelled as 0 and beta cells as 1 prior to importing the dataset into gSELECT.

The goal of this scenario was to examine gene ranking and classification performance in settings where group separation is visually apparent.

#### 3.3.1 Classification performance in clearly separated cell types

The previous scenarios demonstrated how gSELECT can be utilised as a pre-analysis tool to assess whether two groups differ in their transcriptomic profiles. As demonstrated in Fig. 2, this includes testing whether a machine learning model can distinguish between groups when trained on all available genes.

**Fig. 5:**
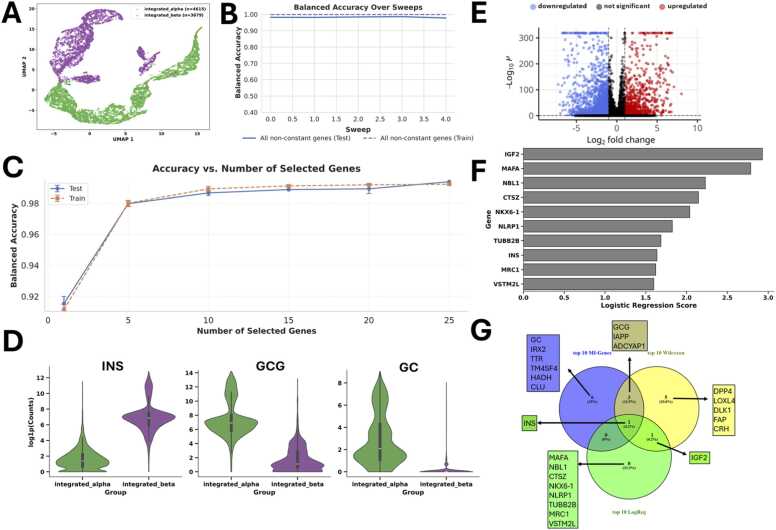
Classification performance and marker evaluation for alpha and beta cells in the panc8 dataset. **(A)** UMAP projection of integrated alpha (green) and beta (purple) cells from the panc8 dataset. The two cell populations show clear separation. **(B)** Balanced accuracy across five Monte Carlo sweeps using all non-constant genes. Accuracy remains at approximately 99 %-100 % for both training and test sets, indicating that the two groups are fully separable based on global expression. **(C)** Classification performance as a function of the number of top MI-ranked genes. Balanced accuracy increases when the highest-ranked genes are included and reaches a near-saturation plateau around 10 genes. **(D)** Violin plots of the top three MI-ranked genes (*INS*, *GCG*, *GC*), showing strong cell-type-specific expression. *INS* is highly expressed in beta cells, while *GCG* and *GC* are enriched in alpha cells. **(E)** Volcano plot showing results from differential expression analysis using Seurat’s FindMarkers() function, using ‘wilcox’ as test method, thresholds set to 0, visualised with the EnhancedVolcano package, showing significantly (adjusted p-value < 0.05) downregulated (log2 fold change < −1) genes in blue and significantly upregulated (log2 fold change < −1) genes in red). Out of 34363 genes, 2873 were significant DEGs, with 897 being upregulated and 1976 being downregulated. **(F)** Logistic regression feature importance scores show *IGF2* as one of the top MI genes. **(G)** Venn diagram comparing the top 10 genes identified by mutual information (blue ellipse), Wilcoxon test (via Seurat’s FindMarkers() function, yellow ellipse), and logistic regression (via scanpy’s rank_genes_groups() function, green ellipse). Only *INS* is shared across all three methods. Wilcoxon and MI ranking identified *INS*, *GCC*, *IAPP*, and *ADCYAP1* as shared top 10 significant genes. Most of the top 10 genes are unique to one or two selection strategies, showing methodological differences in gene prioritisation.

In this example, two transcriptionally distinct cell types were compared: alpha and beta cells from human pancreatic islets. These cell types differ in both function and gene expression, including the production of glucagon and insulin, respectively [35]. By selecting alpha and beta cells for the analysis, we are also able to examine gene ranking and classification performance in settings where group separation is visually apparent (Fig. 5).

As expected, the UMAP projection of the integrated dataset shows clear separation between the two populations (Fig. 5A). We first trained a classifier using all non-constant genes. In contrast to the noise-only scenario in Fig. 2, the model achieved near-perfect accuracy. This confirms that the expression profiles of alpha and beta cells are clearly distinguishable (Fig. 5B). This is also confirmed by the volcano plot, which is based on the genes identified using Seurat’s FindMarkers() function (using ‘wilcox’ as test method, thresholds set to 0, visualised with the EnhancedVolcano package [36], Fig. 5E).

Next, we tested how many genes are required for high classification performance. A single gene, insulin (*INS*), was sufficient to reach over 90 % balanced accuracy. This gene, a known marker of beta cells [35], [37], [38], [39], was ranked highest by mutual information. All three top-ranked genes (Fig. 5D), *INS*, glucagon (*GCG*), and group-specific component (vitamin D binding protein) (*GC*), are known important markers for alpha or beta cells, respectively. With the ten top-ranked MI genes, accuracy increased to ∼99 % (Fig. 5C). Most of these genes are known markers for alpha or beta cell types.

To contextualise these results, we compared MI-based gene rankings with other common selection strategies. Differential expression analysis (Wilcoxon test via Seurat’s [24], [25], [26], [27], [28] FindMarkers() function) and logistic regression (using scanpy’s rank_genes_groups() [21], Fig. 5F) yielded gene sets that overlapped only partially with the MI ranking. *INS* was the only gene consistently identified by all three methods (Fig. 5G). This reflects methodological differences in feature selection and illustrates how MI-based ranking can complement other approaches in early-stage analyses.

As a pre-analysis tool, gSELECT enables early identification of genes with high predictive value and supports classification-based assessment of gene sets in settings with strong biological separation. In this scenario, comparing alpha and beta cells, key markers such as *INS* and *GCG* were identified with very high balanced accuracy. By comparing mutual-information-based rankings to, for example, differential expression and logistic regression results, this example shows how gSELECT can support marker prioritisation and offers an additional layer of evidence that can complement standard selection methods, particularly in the initial stages of analysis.

#### 3.3.2 Evaluation of user-defined gene sets

In addition to ranking genes by mutual information, gSELECT allows evaluating the predictive power of user-defined gene sets. If such a list is provided, classification is performed directly based on the selected features. To the best of our knowledge, at the time of publication, gSELECT is the only available tool that allows direct input of custom gene sets to evaluate their classification performance on a given dataset using a consistent machine learning framework.

This functionality can be used as part of a pre-analysis step to examine whether a set of literature-derived, or AI-provided, such as from AI-assisted search engines or chatbots, or hypothesis-driven genes is informative in a specific dataset. To illustrate this use case, we tested three manually selected gene panels in the comparison between alpha and beta cells.

**Fig. 6:**
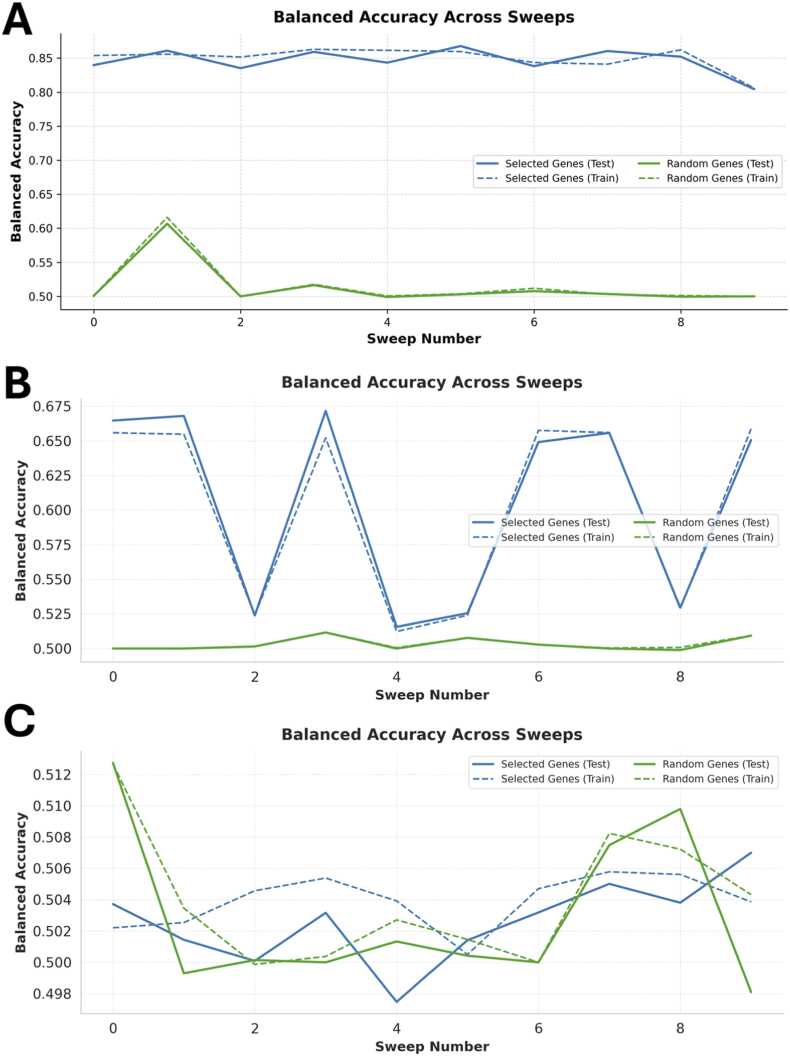
Evaluation of User-Defined Gene Sets Using gSELECT. Balanced accuracy across multiple classification sweeps (x-axis) is shown for three manually defined gene panels in an alpha vs. beta cell setting. For each panel, the performance of selected genes (blue lines) is compared to random gene sets of equal size (green lines). Solid lines represent test set accuracy; dashed lines denote training set accuracy. **(A)** Literature-based alpha/beta markers (*ALDH1A1*, *B2M*, *ALCAM*, *ALDOA*) achieve high predictive accuracy (>85 %), substantially outperforming random sets. **(B)** Ductal markers (*KRT19*, *CFTR*, *SOX9*, *ANXA4*) show moderate predictive power (∼65 %), indicating limited discriminative value between alpha and beta cells. **(C)** Brain-specific genes (*MBP*, *NEFL*, *GRIN1*, *SLC17A7*, *DCX*) perform at chance level (∼50 %), confirming their irrelevance in this context.

In the first example (Fig. 6A), we selected four genes that are not among the top-ranked MI genes in this dataset but have been described in the literature as beta or alpha cell markers: *ALDH1A1* [38], [39], *B2M* [38], [39], *ALCAM* [38], [39], and *ALDOA* [38], [39]. Panels of this kind may arise when known markers are extracted from previous studies (e.g. Dorrell et al. (2011) [40], [41], Segerstolpe et al. (2016) [35], van Gurp et al. (2022) [38], [39]). Classification based on this gene set achieved a balanced accuracy of over 85 % (blue lines in Fig. 6A), indicating that these genes seem to contain a discriminative signal in this context. A comparison with random gene sets of equal size (green lines in Fig. 6A) yielded accuracies near chance (∼50 %). However, based on the used data set, our six suggested genes might be a better set of marker genes, as its expression profile allows a more accuracy separation of the cell types. Apart from the correlation of the expression pattern of the selected genes with the cell type, these genes might also be a starting point for a causal explanation why the differences of the expression of these genes cause the differences in the phenotype.

In a second example (Fig. 6B), we evaluated a set of genes associated with pancreatic ductal cells: *KRT19* [35], [42], *CFTR* [43], *SOX9* [42], [44], and *ANXA4* [44]. As these genes are not expected to distinguish alpha and beta cells, lower classification performance was anticipated. The observed accuracy was ∼65 %, supporting the expectation of limited group separation.

Finally, we tested five genes typically expressed in neuronal tissue (*MBP* [45], [46], [47], [48], *NEFL* [45], *GRIN1* [45], [48], *SLC17A7* [47], [48], *DCX* [46]) but not in the pancreas (Fig. 6C). Classification performance in this case remained at chance level (∼50 %).

#### 3.3.3 Exploratory Subset Evaluation

One of the interesting functions of gSELECT is the ability to perform exploratory search across combinations of genes to identify compact panels with high predictive performance.

This functionality can be used as part of a pre-analysis step to investigate whether small subsets of genes are sufficient to distinguish two groups of interest, and to explore whether compact gene combinations provide sufficient discriminative power in a given setting. Unlike simple ranking, which evaluates genes independently, the exploratory function tests all possible combinations and thus considers cross information between genes. Furthermore, since the separated ranking does not account for redundancy across the genes within a set, this method is an option to reduce redundancy in the selected gene set without a loss of predictive power. However, it is also possible to balance the number of genes and thus the complexity of a model with the predictive power.

It can be used not only on MI-ranked features but also on user-defined gene sets. This enables evaluation of small, hypothesis-driven gene panels to determine which combinations, or individual genes within them, carry the highest predictive signal. This function returns a ranked list of gene combinations, each annotated with their mean balanced accuracy and standard deviation across repeated Monte Carlo sweeps.

Two search modes are supported: exhaustive and greedy. In exhaustive mode, all non-empty subsets of the top n MI-ranked genes are evaluated. The number of combinations grows exponentially with *n*, following a $2^{n}-1$ growth pattern where n represents the number of genes (e.g. 10 genes → 1023 subsets; 15 genes → ∼32000 subsets; 20 genes → >1 million subsets), which quickly becomes computationally infeasible for large *n*. To address this, gSELECT switches to greedy mode by default if *n* exceeds 10. This threshold can be manually adjusted. Greedy mode incrementally assembles gene panels by selecting one additional gene at each step, based on improvements in classification performance. Optional extensions such as beam search or gene swapping (backtracking), as described in the methods, can be enabled to refine the result further. All evaluations are run using parallel execution, and the number of threads can also be adjusted.

Here we first show an example, where we selected five genes based on prior knowledge: two typically expressed in pancreatic ductal cells (*SOX9* [42], [44], *KRT19*[35], [42]), two with known expression in alpha or beta cells (*B2M* [38], [39], *ALDOA* [38], [39]), and *INS*, which ranked highest by mutual information in the current dataset and is also a well-known marker of beta cells [35], [37], [38], [39]. This set reflects a potential real-world use case, where a researcher may wish to test whether a group of candidate genes, derived from literature or previous experiments, contains a signal relevant to a classification task, such as finding a small but meaningful set of genes that can be used as markers for the cell types or the different conditions (e.g. treated and untreated).

**Fig. 7:**
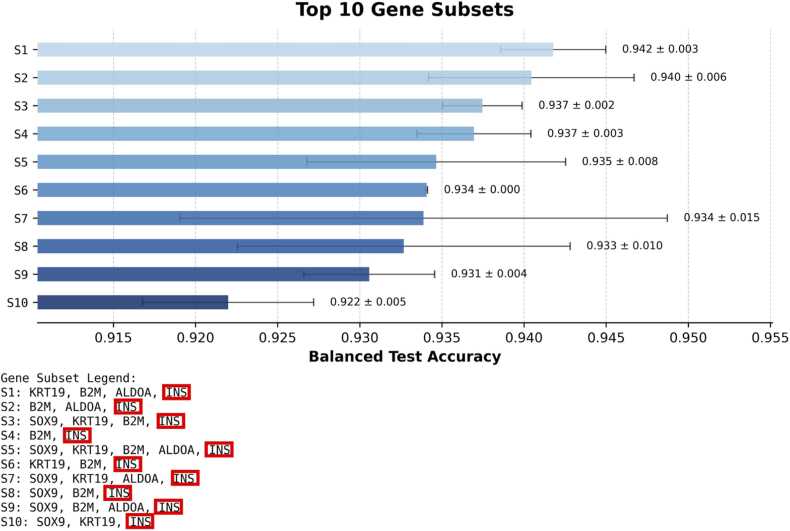
Visualisation of the ten top-ranked gene subsets for different combinations of INS (top-ranked MI gene and well known beta cell marker), B2M and ALDOA (known as alpha and beta cell marker, respectively), and SOX9 and KRT19 (known markers for pancreatic ductal cells).

We applied exhaustive search to evaluate all non-empty subsets of these five genes (Fig. 7). As expected, *INS* consistently appeared in the top-performing subsets, and combinations including *INS* achieved the highest classification accuracy.

Table 5 shows all combinations and their ranking. Each row shows the mean balanced test accuracy achieved with a specific gene subset. Combinations including *INS* are grouped separately, as they consistently outperform subsets without *INS*.

**Table 5:** Classification performance of gene subsets selected from *INS*, *SOX9*, *KRT19*, *B2M*, and *ALDOA*.

**mean balanced test accuracy**	**gene_subset**	
0.94	*KRT19*, *B2M*, *ALDOA*, *INS*	**Combinations with*****INS***
0.94	*B2M*, *ALDOA*, *INS*	
0.94	*SOX9*, *KRT19*, *B2M*, *INS*	
0.94	*B2M*, *INS*	
0.93	*SOX9*, *KRT19*, *B2M*, *ALDOA*, *INS*	
0.93	*KRT19*, *B2M*, *INS*	
0.93	*SOX9*, *KRT19*, *ALDOA*, *INS*	
0.93	*SOX9*, *B2M*, *INS*	
0.93	*SOX9*, *B2M*, *ALDOA*, *INS*	
0.92	*SOX9*, *KRT19*, *INS*	
0.92	*ALDOA*, *INS*	
0.92	*SOX9*, *ALDOA*, *INS*	
0.92	*KRT19*, *ALDOA*, *INS*	
0.91	*SOX9*, *INS*	
0.91	*KRT19*, *INS*	
**0.91**	***INS***	
0.76	*SOX9*, *KRT19*, *B2M*, *ALDOA*	
**0.75**	***B2M*, *ALDOA***	
0.73	*KRT19*, *B2M*, *ALDOA*	
0.54	*SOX9*, *KRT19*, *ALDOA*	
0.54	*SOX9*, *B2M*, *ALDOA*	
0.51	*SOX9*, *ALDOA*	
0.51	*KRT19*, *ALDOA*	
0.51	*ALDOA*	
0.51	*SOX9*, *KRT19*	
0.50	*SOX9*, *KRT19*, *B2M*	
0.50	*SOX9*, *B2M*	
0.50	*SOX9*	
0.50	*B2M*	

As expected, *INS* was the most informative individual marker, resulting in a balanced accuracy of 0.91. Gene sets that included INS achieved the highest classification performance, with accuracies reaching up to 0.94.

In comparison, subsets without *INS* resulted in a substantially lower accuracy. The best-performing *INS*-free combination, consisting of *B2M* and *ALDOA*, reached 0.75 balanced accuracy. In contrast, *B2M* or *ALDOA* alone produced near-random performance (0.50–0.51), indicating limited discriminatory value when used individually.

Gene sets composed solely of *SOX9* and *KRT19* likewise performed at baseline level, reflecting their low relevance for distinguishing the cell types in this setting.

Together, the presented analyses demonstrate that gSELECT can be used as a pre-analysis tool to assess the extent to which differences in gene expression between groups are sufficient for classification. In contrast to methods that focus solely on statistical significance, gSELECT emphasises predictive performance and supports evaluating both data-driven and hypothesis-based gene sets in a unified framework.

In particular, the ability to directly test user-defined gene panels, whether derived from prior studies, experimental hypotheses, or other selection strategies, such as conversational AI, offers a way to contextualise their relevance in a given dataset. This may help inform decisions about downstream validation, prioritisation of markers, or the development of reduced gene panels.

Although classification performance alone should not be interpreted as a substitute for biological interpretation, the results shown here indicate that gSELECT can support early-stage evaluation and guide further exploration of transcriptomic patterns in single-cell data, such as causal explanations that drive the observed differences and what influences might cause a significant change on such observations, e.g. the cell fate. We remark that for such further investigations conversational AI or AI assistants that are grounded with a web search might be a useful extension for coming to causal explanations. More details and descriptions about the combination of gSELECT and conversational AI for validating the results and building further explanations, in particular with AI-assisted literature research, can be found in the supplement under Additional Literature Validation of Selected MI Genes.

## 4 Discussion

Computational methods differ in their underlying assumptions and sensitivity to data characteristics and have been benchmarked and discussed in detail for single-cell RNA Sequencing by Krzak et al. (2019) [49]. This can lead to two critical issues:

1. False discoveries: A method might detect an effect that is actually an artifact of the computational approach rather than a real biological signal. For instance, differential expression (DE) analysis may highlight a gene as important which does not carry much information about the phenotypic difference compared to other genes. Similarly, dimensionality reduction techniques such as UMAP or t-SNE may fail to accurately separate phenotypic differences if the biological variation in the data does not exclusively align with the phenotypic difference of interest.
2. Missed discoveries: Some methods may fail to detect relevant genes due to their inherent assumptions. Principal Feature Analysis (PFA) [8], [9] may delete too many genes if assumptions are not valid or Principal Component Analysis (PCA) [50], for example, assumes linear relationships and orthogonality constraints, which may lead to a loss of important features relevant to phenotype differentiation, where the linearity of relations between gene expression and phenotypic differences might not always hold.

Consequently, we need to doublecheck computational results to avoid missing important information from the data and thus missing opportunities to get insights or to schedule lab experiments based on false analysis results, which wastes valuable resources.

Different methods often focus on different aspects of the data. Combining multiple strategies can help reduce the likelihood of overlooking important patterns or overinterpreting spurious relations.

### 4.1 Intended use of gSELECT

There are four core use cases for using gSELECT as a pre-analysis tool:

**1. Dataset-Wide Assessment.** Determines whether expression profiles differ between experimental conditions (e.g. treated vs. untreated cells), based directly on count data. Before investing time and resources into downstream analysis, gSELECT can be used to evaluate whether a selected dataset contains sufficient discriminative signal. This is particularly useful when group differences are subtle or uncertain, as demonstrated in the results with our synthetic data and sex-difference scenarios.

**2. Gene Ranking.** Mutual information ranking enables the identification of genes with high predictive value, independently of their statistical significance in DE testing. gSELECT uses mutual information and ML to rank genes by their relevance for distinguishing between groups, with the top-ranked genes being the most relevant genes for differentiating between the groups. This complements existing tools for analysing single cell data such as Seurat [24], [25], [26], [27], [28] by focusing on the predictive power of individual genes. It also quantifies how well selected genes, either top-ranked or user-defined, can distinguish between sample groups. The default pipeline for gSELECT calculates mutual information on the complete set of expression data; however, it also includes an option to split the dataset before computing mutual information, which is demonstrated in the Tutorial in the Supplement. In this mode, a designated training subset (e.g. 80 %) is used exclusively for feature selection and model fitting, while the remaining data is held out for final evaluation. This provides a safeguard against potential circularity, especially when reporting gene panels with claimed predictive power and is also a good option when a stricter separation of analysis steps is desired.

**3. Evaluation of custom or literature-derived gene sets.** This is one of the main advantages of gSELECT, as it enables the evaluation of custom gene sets from literature, predictive models, or prior biological knowledge. Using this function can support hypothesis-driven workflows and help decide whether a known gene panel is relevant in a given dataset before setting up further bioinformatic analyses or laboratory experiments.

**4. Exploration of compact gene panels.** By testing small subsets of selected genes, gSELECT can help identify gene combinations with high predictive power. This functionality can be useful for experimental follow-up studies (e.g. qPCR validation, targeted panels), where resource constraints require prioritisation.

### 4.2 Performance of gSELECT in different scenarios

As previously mentioned, we tested gSELECT in three scenarios: (1) two very similar groups, (2) two rather similar groups according to UMAP analysis, which might prompt the question whether further analysis is worthwhile, and (3) two clearly different groups, which are separated in UMAP clustering. The first two scenarios were used to test the basic functionalities of gSELECT, while the third scenario demonstrated the two novel functions of gSELECT: evaluating custom genes of interest and calculating the predictive value of different gene combinations.

**In the first scenario**, the balanced accuracy of analysing all non-constant genes in the dataset, which contained very similar data in both groups (only altered by 1 % of Gaussian noise) was around 50 %, which equals the accuracy of mere guessing. This was to be expected due to the similarity of the two groups and shows that a gSELECT analysis with a low prediction accuracy can indicate whether there is no information in the data to separate the groups accordingly. Additionally, the high number of misclassified samples when using all genes for prediction further confirms the similarity of the two groups.

This scenario demonstrates the use of gSELECT as a pre-analysis tool. If run directly after obtaining the h5ad file, a result resembling that of the first scenario suggests high similarity between the analysed groups, indicating that further analysis of these groups is unlikely to yield additional meaningful insights. Therefore, a gSELECT pre-analysis can help to avoid wasting time and resources. Additionally, a second analysis method, such as the Wilcoxon rank sum test, which is available via standard analysis tools, such as, for example, Seurat [24], [25], [26], [27], [28] or scanpy [21], can be employed.

**In the second scenario**, we applied gSELECT to a SMART-Seq v4 dataset of the adult mouse isocortex and hippocampal formation [17]. Instead of replicating the original clustering into GABAergic and glutamatergic neurons, we focused on sex-based separation (female vs. male) across all neuronal cell types. Although UMAP projections showed only subtle differences between groups, gSELECT identified a subset of genes with mutual information. Among the top-ranked features were known sex-specific markers such as *Xist* and *Eif2s3y*, which have established roles in female and male gene regulation, respectively. This example demonstrates that gSELECT can identify a predictive signal in biologically plausible yet weakly separable cases.

To further assess the diagnostic value of gSELECT, we explicitly removed the top-ranked sex-specific genes (*Tsix, Xist, Eif2s3y, Ddx3y, Uty, Kdm5d*) from the analysis and reevaluated the classification performance. This exclusion step tested whether the remaining signal still allowed meaningful separation between male and female cells. Balanced accuracy dropped from ∼99.5 % to near-random ∼56 % when only ten of the remaining top-ranked MI genes were used, confirming that the removed markers captured most of the group differences. However, when the number of features was increased to ∼25, test accuracy improved and plateaued at ∼72 %, while training accuracy continued to rise. This indicates a residual, but weaker, discriminative signal, which helps determine whether a dataset contains predictive information beyond the dominant known features, while also hinting at a potential indication of overfitting.

**In the third scenario**, we applied gSELECT to a well-characterised single-cell dataset of human pancreatic islet cells ("panc8" of SeuratData [19]) to assess classification performance between distinct cell types, alpha and beta cells. The integrated dataset comprises samples from four studies, generated using different technologies, and represents a standard testbed for benchmarking analysis methods. Unlike the previous scenarios, visual inspection via UMAP revealed a clear separation between the two groups, and a model trained on all non-constant genes reached near-perfect classification accuracy. As expected, the mutual information-based ranking recovered canonical markers, such as *INS*, *GCG*, and *GC*[35], [38], [39], at the top of the list. These genes alone were sufficient to achieve very high classification accuracy, with *INS* individually reaching over 90 % balanced accuracy. The ten top-ranked MI genes, which are also known alpha or beta cell markers, respectively (see Supplement), yielded near-perfect performance (∼99 %). Notably, this performance was achieved without prior knowledge or parameter tuning, indicating that mutual information offers a strong and unbiased selection criterion.

These genes alone were sufficient to achieve very high classification accuracy, with *INS* individually reaching over 90 % balanced accuracy. The ten top-ranked MI genes yielded near-perfect performance (∼99 %). Notably, this performance was achieved without prior knowledge or parameter tuning, indicating that mutual information offers a strong and unbiased selection criterion.

The comparison of MI ranking with differential expression analysis and logistic regression highlighted overlaps (e.g. *INS* as a shared top gene), but also revealed method-specific selections, which may reflect differences in sensitivity and assumptions across the methods. This observation shows that different methods capture different aspects of the data, depending on their underlying assumptions and ranking logic. The subsequent validation step mapping predictive power to a set of genes regarding the (phenotypic) difference of interest is a helpful step to quantify the results of each selection method with regard to the information content of the corresponding gene set and the separation of the labels of interest.

However, the added value of gSELECT in this specific case lies less in the identification of known markers, since other established methods would also recover these, but in its ability to evaluate custom gene sets.

This function was used in the third scenario to assess manually curated gene panels, including known islet cell markers not ranked among top ten MI genes, ductal cell markers, and brain-specific genes.

The results showed that some literature-derived markers achieved high classification accuracy (>85 %), even if they were not highly ranked by MI, whereas irrelevant gene sets performed at a near-chance level. A reason can be that although each gene has a low MI-rank, there exists cross information over the whole set of genes, meaning that when considering the expression of each gene together a high predictive power can be generated. To also find such cross information scenarios is one use case for the exploratory search.

Nevertheless, we note that balanced accuracy should be interpreted cautiously: a high score indicates that the gene set effectively separates the groups but might not imply causality or biological function.

The third scenario also demonstrates the use of gSELECT's exploratory function. The exploratory function of gSELECT offers two distinct search strategies: exhaustive and greedy. In the alpha vs. beta cell setting, exhaustive subset selection was used to evaluate all possible combinations of five biologically motivated genes.

To evaluate the predictive power of known and candidate marker combinations in the alpha vs. beta cell comparison, we applied exhaustive subset selection to a panel of five genes:

• *INS*, the top-ranked gene by mutual information and a well-established beta cell marker [35], [37], [38], [39].
• *B2M* and *ALDOA*, both supported by literature as being expressed in pancreatic alpha and beta cells [38], [39], but not among the top ten MI genes in this dataset.
• Moreover, *SOX9* and *KRT19*, genes typically associated with ductal cell identity [35], [42], [44] and not expected to distinguish alpha from beta cells.

Given its known role and expression pattern, high classification accuracy was expected for subsets containing *INS*. Indeed, *INS* alone achieved ∼91 % balanced accuracy, confirming its strong predictive capacity. The central question was whether *B2M* and *ALDOA*, despite not being among the top-ranked MI genes, could contribute additional, non-redundant information when combined with *INS*, thereby improving classification beyond what *INS* could achieve alone.

This expectation was supported by the results: combinations including *INS*, along with *B2M* and/or *ALDOA*, reached accuracies of up to ∼94 %, slightly exceeding the performance of *INS* in isolation. This suggests that these additional genes, while not individually highly ranked, provide a cross-supporting discriminatory signal that complements the dominant effect of *INS*. An interpretation could be that the underlying causal mechanism is determined by at least these two genes. Subsets composed exclusively of *SOX9* and *KRT19* genes, which are expressed in pancreatic tissue but not explicitly associated with alpha or beta cell identity, failed to deliver meaningful classification performance. The best combinations among these genes yielded accuracies around 50–54 %, equivalent to random chance. These findings align with prior biological expectations that *SOX9* and *KRT19* are not relevant for distinguishing islet endocrine subtypes, and their presence does not improve model accuracy.

This analysis demonstrates how gSELECT can be used to identify whether genes within a panel provide redundant or complementary information. This can be relevant when designing reduced marker panels (e.g. for targeted assays or diagnostics), as they help determine which genes add value in combination and which do not improve classification beyond what is already captured by the top markers. The different scenarios and the respective applications of gSELECT are summarised in Table 6.

**Table 6:** Summary of gSELECT performance across representative scenarios.

**Scenario**	**Dataset and Comparison**	**Objective / Context**	**Key Results**	**Interpretation**
1. Minimal differences (negative control)	Artificial dataset based on data by Xue et al. [15] with 1 % Gaussian noise added to one group	Test gSELECT's ability to detect separability when no meaningful difference exists	Balanced accuracy ≈ 50 %; high number of misclassified samples	No detectable group-level signal. Suggests gSELECT can serve as an effective early-stage filter to avoid unnecessary downstream analyses
2. Subtle biological signal (sex classification)	SMART-Seq v4 data from adult mouse isocortex and hippocampus by Yao et al. [17]; male vs. female	Evaluate (i) whether classification is feasible despite no visible clustering in UMAP visualisation, and (ii) whether remaining genes carry predictive information once the strongest markers have been removed.	Top MI genes (*Xist*, *Eif2s3y*) yield ≈ 99.5 % accuracy. After exclusion of the top MI genes: test accuracy drops to ≈ 56 %, recovers to ≈ 72 % at ≥ 25 genes	gSELECT detects informative markers even in weakly separable settings; divergence between training/test accuracy reveals overfitting onset (∼25–50 genes)
3. Clear biological difference (alpha vs. beta cells)	panc8 dataset [19] (integrated human pancreatic islets); alpha vs. beta cell types	Evaluate both data-driven and literature-based gene sets; test combinatorial predictive value	*INS* alone achieves > 90 % accuracy; top 10 MI genes ≈ 99 %; curated panel (*INS*+*B2M*+*ALDOA*) slightly higher; irrelevant gene sets (e.g. *SOX9*, *KRT19*) ≈ 50 %	gSELECT recovers canonical markers and distinguishes between informative and redundant genes; supports rational panel refinement and validation of known or proposed markers

### 4.3 Limitations of gSELECT

While gSELECT offers a way to prioritise genes and test subsets, some limitations must be considered to ensure accurate interpretation of the results and responsible use. These include conceptual boundaries of the method, data dependencies, and computational constraints, as outlined below:

**Classification performance inherently depends on characteristics of the input dataset**, including group size, sparsity, dropout effects, and potential confounders such as batch effects. While high accuracy may reflect an accurate biological signal, it can also be because of technical artefacts if preprocessing is not adequately addressed. This was a design choice for gSELECT as we did not want to impose mandatory normalisation, batch correction, or integration steps. Instead, we deliberately leave these decisions to the user, allowing maximal flexibility in adapting the analysis to the biological question and data type. This allows gSELECT to be applied at an early stage in the pipeline, even before batch correction or other preprocessing decisions have been finalised.

In single-cell RNA sequencing (scRNA-seq), the total amount of transcribed mRNA per cell varies due to biological factors such as cell volume and growth state [51]. Additionally, gene expression levels are influenced by the cell cycle, with some genes exhibiting periodic fluctuations throughout different phases of the cell cycle [51], [52], [53]. Due to technical artifacts and biological heterogeneity in scRNA-seq data, normalisation methods for bulk RNA-seq cannot be applied directly [54]. Variations in the number of genes or molecules detected in the cells (sequencing depth) also need to be considered during normalisation in scRNA-seq data [55].

However, normalisation correcting for cell size can also correct for variations due to cell cycle effects [56] and thus remove meaningful information. Normalising based on genes that are strongly regulated by the cell cycle can introduce bias, which might complicate data interpretation and analysis [57]. Due to the impact of normalisation on the accurate identification of differentially expressed (DE) genes [58] and downstream analyses [54], it is critical to assess whether a given normalisation method preserves biologically relevant variation or inadvertently removes important signals.

In some cases, it may not be clear whether normalising the data is helpful or whether it discards valuable information. Our proposed interpretation of the model’s accuracy, in terms of predictive power, can be used as a data-driven approach to decide which data preprocessing is more suitable for a specific research question, as encoded in the labels. Since the labels are independent of the gene expression preprocessing procedure, by performing several procedures, we can determine which gene selection (based on specific preprocessing steps) has the highest predictive power regarding the differences of interest (labels). A posteriori, we can then justify a certain pre-processing procedure as being the most suitable one, deleting only the least amount of helpful information, or in other words, preserving the most useful information for separating the labels.

In our analysis of the panc8 dataset, we evaluated how batch effects could affect the MI-based gene selection by analysing alpha and beta cell data before and after integration to correct the batch effect (see Supplementary Results for the Analysis of the Multi-Batch Pancreas Data for further details). No substantial differences in MI gene ranking were observed across batches in this case, as also demonstrated in the analysis of the non-integrated data in the Supplement. This suggests that the observed signal was not primarily driven by batch structure. gSELECT can be applied to both raw and pre-processed data, which allows for comparisons across different preprocessing stages.

**Like all machine learning–based approaches, gSELECT is susceptible to overfitting,** particularly in settings with limited sample size, high dimensionality, or repeated evaluation of many gene combinations. Although standard safeguards, such as Monte Carlo cross-validation and train-test splits, are included, classification-based evaluation always carries an inherent risk of detecting artificial patterns. Overfitting can be explained as when the model begins to memorise dataset-specific noise rather than learning generalisable patterns [59]. This became apparent in our second scenario where sex-linked genes were purposefully excluded. As expected, balanced accuracy dropped markedly, especially for the test set. However, training accuracy remained relatively high, resulting in a growing divergence between training and test performance across different gene set sizes. In our case, we observed a growing gap between training and test accuracy after approximately 25–50 genes, indicating potential overfitting to noise or non-generalisable patterns. gSELECT visualisations make this divergence observable, helping to detect overfitting behaviour early and, depending on the biological context, still extract functional subsets before the breakdown point for further exploratory or confirmatory analysis.

**All results presented in this study are based on in silico analyses.** While the findings align well with established biological knowledge, for instance, the identification of canonical islet markers in the pancreas scenario or known sex-linked genes in the sex classification task, no experimental validation has been performed. The predictive performance of certain gene sets, including gene combinations based on literature research or prior knowledge, supports the potential relevance of the respective genes. However, these findings should also be confirmed by further experimental analysis before functional conclusions are drawn.

### 4.4 Computational constraints and scalability

**The computational cost of gSELECT depends strongly on dataset size, gene panel size, and chosen analysis mode**. Memory requirements increase with dataset size. Although sparse matrix formats are supported during loading, gSELECT internally converts expression data to dense format for compatibility with matrix operations and classification routines. This can result in elevated RAM usage, particularly when analysing large single-cell datasets. Several components of gSELECT, particularly exhaustive or greedy subset evaluation and repeated classification, can lead to high memory usage, especially in large datasets or when using multiple parallel threads. Parallelisation is handled via Python’s ThreadPoolExecutor.

gSELECT detects the number of available CPU cores by default and adjusts parallelisation accordingly. It is also possible to set the number of threads manually (via the num_threads parameter) to limit memory usage. This allows balancing runtime versus memory usage depending on system constraints. To make resource usage transparent, gSELECT includes an integrated reporting feature that logs peak memory usage during runtime and allows for monitoring whether analyses exceed system capabilities and adjusting accordingly.

**gSELECT uses repeated train/test splits (Monte Carlo cross-validation) to assess classification stability.** Reducing the number of sweeps or switching to greedy search lowers the runtime. While a small number of sweeps may suffice in preliminary analyses, more repetitions are recommended to get a reliable uncertainty and best values of the predictive power (model accuracy), where the standard deviation of the model accuracy provides us a measure for how much the values depend on the data sampling and the non-deterministic mechanisms during training.

**Exhaustive subset evaluation scales exponentially.** In exhaustive mode, all combinations of the selected genes are evaluated based on their balanced accuracy, enabling the identification of the optimal combination in terms of classification accuracy. Testing all possible combinations comes with a computational cost: the number of combinations increases exponentially with the number of input genes (e.g. 15 genes → ∼32000 subsets; 20 genes → >1 million). In practice, this mode is feasible only for relatively small panels and requires considerable computing resources and memory.

When analysing a larger set of genes, gSELECT offers a greedy search mode as a scalable alternative for evaluating larger gene sets.

In greedy mode, the algorithm incrementally constructs gene panels by iteratively selecting the following gene that yields the highest gain in classification performance. At each step, the gene that maximises improvement in balanced accuracy is added to the current subset.

It is considerably faster than exhaustive search and scales to larger gene sets, but may miss the globally optimal combination, especially when predictive signals depend on interactions between genes. Greedy selection may converge on a locally optimal but globally suboptimal combination. To address this, gSELECT offers a configurable search depth, beam search, and optional backtracking. These options could improve search results, especially for medium-sized gene sets. Still, greedy strategies have known limitations and should be used with caution, in particular, when the goal is to prioritise genes for biological interpretation, not just classification.

Despite its inherent limitations, the greedy strategy in gSELECT can be a good choice, particularly in settings where exhaustive search is computationally infeasible. In practice, many applications of gene panel selection do not require the identification of a globally optimal subset but instead aim to identify a small set of genes with sufficient predictive power for follow-up validation or experimental design. Therefore, gSELECT automatically switches to greedy selection when *n* exceeds a threshold (default: 10), but this can also be changed, as it is implemented as a variable.

**Exploratory evaluations in gSELECT are parallelised by default,** and the number of threads is set automatically based on available CPU cores. It can also be configured manually via the num_threads parameter. Reducing the number of threads may lower memory usage, which can be important when working on shared or memory-constrained systems.

## 5 Conclusion

gSELECT provides a framework for evaluating the predictive power of genes and gene sets in single-cell transcriptomic data. It complements existing methods by offering a model-based assessment of dataset separability, grounded in mutual information ranking and classification performance. Additionally, the ability to evaluate both data-derived and custom predefined gene sets, as well as to explore combinatorial effects of different genes of interest, enables users to identify potentially informative genes prior to downstream analysis.

Furthermore, gSELECT offers two novel functions, which, to the best of our knowledge, represent the first of their kind in this field: Analysing the predictive power of a custom gene or a group of genes of interest, and calculating the predictive power of different gene combinations and ranking them. This is particularly useful in early-stage hypothesis refinement and the prioritisation of candidate markers under practical constraints. While not a replacement for significance testing or mechanistic interpretation, gSELECT offers a quantitative perspective on the information content of expression features with respect to predefined group labels. Its modular design and compatibility with standard data formats allow for integration into diverse analytical workflows, supporting more informed decisions in exploratory and confirmatory settings.

## CRediT authorship contribution statement

**Tim Breitenbach:** Writing – review & editing, Writing – original draft, Supervision, Project administration, Methodology, Conceptualization. **Aylin Caliskan:** Writing – review & editing, Writing – original draft, Visualization, Validation, Project administration, Methodology, Investigation, Formal analysis, Data curation, Conceptualization. **Deniz Caliskan:** Writing – review & editing, Writing – original draft, Visualization, Supervision, Software, Methodology, Investigation, Data curation, Conceptualization. **Thomas Dandekar:** Writing – review & editing, Supervision, Funding acquisition.

## Funding

We acknowledge funding by DFG (10.13039/501100001659German Research Foundation) Project number 492620490 (CRC 1583/INF DECIDE).

## Code availability

The relevant code for gSELECT and our analyses is available via GitHub at https://github.com/CaliskanDeniz/gSELECT.

## Declaration of Competing Interest

The authors declare that there is no conflict of interest.
