# Supplementary material for: gSELECT: A novel pre-analysis machine-learning library enabling early hypothesis testing and predictive gene selection in single-cell data
Source: Comput Struct Biotechnol J. 2025 Aug 5;27:3510–27. doi: 10.1016/j.csbj.2025.07.047 (PMC12354962; doi:10.1016/j.csbj.2025.07.047)
Supplement: Supplementary file 1 — Supplementary material [file mmc1.pdf]

Content

Supplementary Method Descriptions 2

Dataset Acquisition and Processing 2

Quality Control and Filtering 3

Normalisation and Feature Selection 3

Synthetic Data Generation 3

Dimensionality Reduction and UMAP Analysis 4

Differential Expression Analysis and Volcano Plot 4

Validation of the gSELECT MI Genes Using the R-package Seurat 5

Survival Analysis Using TCGA-LUAD Data 6

Clinical and RNA-Seq Data Processing 6

Kaplan-Meier Survival Analysis 6

Additional Validation of gSELECT with Different Datasets 8

Data Processing and Differential Expression Analysis of Mouse Cortex Data 8

Data Processing and Differential Expression Analysis of Multi-Batch Pancreas Data 10

Supplementary Results for the Analysis of the Multi-Batch Pancreas Data 13

Additional Information on the MI Genes for the Comparison between Pancreatic Alpha and Beta Cells 16

Case Study Using a Lung Cancer Cell Line 19

Overview of the Case Study Analysis Pipeline 19

Second Scenario: Two Rather Similar Groups according to their UMAP-visualisation 20

Third Scenario: Two Clearly Different Groups 24

Top-ranked Mutual Information Genes: Validation by Enrichment Analysis 30

Top-ranked Mutual Information Genes: Validation by Patient Data 32

Novel Function of gSELECT: Validating Accuracy of Gene Sets Suggested from Literature 36

Additional Validation of the gSELECT MI Genes and Benchmarking 41

Seurat FindMarkers 41

Scanpy rank_genes_groups 43

AUROC 46

scClassify 48

***In silico* Validation of the 25 top-ranked MI Genes** 50

Additional Literature Validation of Selected MI Genes 53

Performance of gSELECT in Different Scenarios 55

Second Scenario – rather similar groups in UMAP 55

Third Scenario – distinct differences in UMAP 56

References 62

Supplementary Method Descriptions

Dataset Acquisition and Processing

Single-cell RNA sequencing (scRNA-seq) data were obtained from the Gene Expression Omnibus (GEO) [1, 2] under the accession number GSE137912 (NCBI GEO, https://www.ncbi.nlm.nih.gov/geo/query/acc.cgi?acc=GSE137912). The dataset includes raw count matrices in MTX format, as well as cell and gene annotation files in CSV format. Additionally, we analysed a SMART-Seq v4 dataset (GSE185862) and the "panc8" dataset of the R package SeuratData [3] (https://github.com/satijalab/seurat-data), which consists of four human pancreatic islet cell datasets obtained using different technologies. Further details on the respective datasets and the preparation of the datasets can be found in the Tutorial and in Additional Validation of gSELECT with Different Datasets).

All computations were performed using Python 3.10.9 (Conda-Forge distribution) in Jupyter Notebook 7.3.2 on a Windows 10 system with 24 CPU cores and 64 GB RAM. Libraries used for data processing were Scanpy (1.11.0) [4] for single-cell analysis, Scrublet (0.2.3) [5] for doublet detection, pandas (2.2.2) [6] for data handling, scipy (1.15.1) [7] for matrix operations, numpy (1.24.4) [8] for numerical computations, and matplotlib (3.9.1) [9] for visualisation. Pre-/Post-analysis was performed using gSELECT (https://github.com/CaliskanDeniz/gSELECT).

Raw count matrices were extracted from the MTX files and loaded into AnnData objects using Scanpy. Each dataset was mapped to its corresponding metadata, and gene identifiers were annotated based on the Ensembl [10] GRCh38 (release 113) reference genome, retrieved from ftp.ensembl.org. For each GSM sample, an H5AD file was generated, containing the raw UMI count matrix along with corresponding cell barcodes and gene annotations. Gene identifiers in ENSEMBL format were converted to gene symbols where available. Individual samples were merged into a single AnnData object while preserving sample identity.

Quality Control and Filtering

To ensure high data quality, several filtering steps were applied. Cells with mitochondrial RNA content >10% were removed. Genes belonging to the ribosomal gene families (RPS and RPL) were excluded. Additionally, cells with fewer than 500 total UMI counts or fewer than 10 detected genes were discarded to remove low-complexity samples.

To further refine the dataset, doublets were identified using Scrublet, and cells with high doublet scores were excluded. The effectiveness of this filtering step was validated using UMAP visualisation, comparing cell distributions before and after doublet removal.

Normalisation and Feature Selection

After quality control, library size normalisation was performed by scaling each cell's total counts to 10,000 UMI, followed by log-transformation to stabilise variance. Highly variable genes (HVGs) were identified using two independent approaches: Seurat v3 and Cell Ranger, with the top 2,000 HVGs retained for downstream analyses.

Synthetic Data Generation

To create a controlled synthetic dataset, gene expression data from untreated H358 cells (H358_untreated_as_0_H358_untreated_as_1.csv) was used as a baseline, which was prepared according to the data preparation workflow required for the Principal Feature Analysis, which has been described in detail in the respective publication [11] and is also available at GitHub (https://github.com/AC-PHD/Seurat_PFA_pipeline). A subset of samples originally labelled as Condition 2 was modified by introducing Gaussian noise to simulate a scenario in which a treatment had no biological effect.

For each gene, noise was sampled from a normal distribution with:

$$\mathcal{N}(\mu, \sigma^2)$$

where σ^2 represents the variance and thus the expected deviation of each gene's mean expression μ. In this study, noise a level of 1% (σ^2=0.01) was applied. This approach introduces controlled variability while preserving the overall structure of the expression data.

Dimensionality Reduction and UMAP Analysis

Principal component analysis (PCA) was performed using the selected highly variable genes. A k-nearest neighbour (k-NN) graph was computed, and UMAP embedding was generated with n_neighbors = 15, min_dist = 0.1, and n_components = 2, allowing visualisation of cellular heterogeneity. UMAP was conducted both on the full dataset and on a subset of genes identified through gSELECT, allowing targeted visualisation of biologically relevant genes. UMAP plots were generated for both the full gene set and the top 10 MI-selected genes, with additional custom-selected gene sets being evaluated for their clustering patterns

Differential Expression Analysis and Volcano Plot

Differentially expressed genes (DEGs) for the synthetic Data were identified using a Wilcoxon rank-sum test (Mann-Whitney U test), comparing expression distributions between Condition 1 and Condition 2. Multiple testing correction was applied using the Benjamini-Hochberg false discovery rate (FDR) adjustment. Genes were classified as differentially expressed if they met both criteria:

$$Adjusted\ P - Value\ (FDR)\ <\ 0.05$$

$$|\log_2 FC|\ >\ 1$$

Where the log2 fold change ($\log_2 FC$) was calculated as:

$$\log_2 FC = \log_2 \left(\frac{\text{Mean Expression (Condition 2)}}{\text{Mean Expression (Condition 1)}} \right)$$

A volcano plot was generated to visualise differentially expressed genes, with log2 fold change on the x-axis and -log10 transformed FDR-adjusted p-values on the y-axis. Genes were categorised based on their significance and fold change: Upregulated DEGs (log2FC > 1, FDR < 0.05) were shown in red, and downregulated DEGs (log2FC < -1, FDR < 0.05) were shown in blue. Genes that were significant (FDR < 0.05) but did not meet the log2FC threshold were displayed in green, and non-significant genes were shown in grey. The top 10 most significant genes (lowest FDR-adjusted p-values) were annotated in the volcano plot. To further validate differentially expressed genes, violin plots were generated to compare gene expression distributions across conditions.

Validation of the gSELECT MI Genes Using the R-package Seurat

To validate the genes obtained using gSELECT, which are based on mutual information, we performed standard analyses for single cell data using the R-package Seurat [12, 13] in a virtual Ubuntu (Ubuntu 20.04.2 LTS (OS-Type: 64-bit)) environment on a virtual machine (Virtual Box 6.1.34), with R (4.4.2) [14] and R Studio ("Chocolate Cosmos" Release (e4392fc9, 2024-06-05)) and the previously prepared (please, see data set acquisition and processing) h5ad file, containing filtered raw data, as input.

To load the h5ad file in a compatible format suitable for subsequent Seurat analysis, we used reticulate (1.38.0) [15] to create a conda-environment with Python 3.8, AnnData [4], and pandas [6].

Seurat (4.3.0) [12, 16-18], ggplot2 (3.5.1) [19] and tidyverse (2.0.0) [20] were used to analyse and visualise the data. To find potentially relevant genes, Seurat's FindMarkers() function was employed, using 'wilcox' (for Wilcoxon as standard Seurat method), 'DESeq2' (for DESeq2 (1.44.0) [21] analysis), and 'MAST' (for MAST (1.30.0) [22] analysis). The most relevant genes (by absolute log2 fold change) were visualised as heatmaps using the DoHeatmap() function of Seurat.

Survival Analysis Using TCGA-LUAD Data

To investigate the prognostic relevance of specific gene expression patterns, survival analysis was conducted using TCGA-LUAD (Lung Adenocarcinoma) [23] data. Clinical and transcriptomic data were retrieved from The Cancer Genome Atlas (TCGA, https://www.cancer.gov/tcga) via the GDC Data Portal. The specific dataset version used was Sat_Mar_15_09_24_44_2025.tar.gz.

Clinical and RNA-Seq Data Processing

Clinical metadata for TCGA-LUAD patients was extracted from XML files using GDCquery() and parsed to obtain vital status, days to death, and days to last follow-up. Overall survival time was determined as the maximum available follow-up duration per patient (either days to death or last follow-up). Patients with missing survival times were excluded from further analysis.

For transcriptomic data, RNA-Seq gene expression quantification was retrieved using GDCquery(), selecting STAR-aligned gene counts from primary tumour samples. Gene expression matrices were pre-processed using DESeq2 [21], where genes with low expression (sum of raw counts <10 across all samples) were filtered out, followed by variance-stabilising transformation (VST) normalisation. Ensemble gene identifiers were mapped to gene symbols using the latest annotation from TCGA. Only genes with unambiguous gene symbol annotations were retained.

Kaplan-Meier Survival Analysis

Kaplan-Meier survival curves were generated for both individual genes and multi-gene combinations. For each gene, patients were divided into high-expression (≥ median) and low-expression (< median) groups. The median was computed across the full cohort, ensuring comparability across all patients. Kaplan-Meier survival estimates were calculated using the survfit() function, and statistical significance was assessed using the log-rank test. A p-value < 0.05 was considered statistically significant.

For the multi-gene survival analysis, only patients in whom all selected genes were consistently either of high or low expression were included. Patients were assigned to the ALL_HIGH group if the expression levels of all selected genes were equal to or above the cohort median. Conversely, patients were classified as ALL_LOW if all selected genes exhibited expression levels below the median. To ensure clear distinctions between the survival groups, patients with an inconsistent expression pattern, where some genes were highly expressed while others were lowly expressed, were excluded from the analysis.

Kaplan-Meier survival curves were generated to compare the overall survival distributions between the ALL_HIGH and ALL_LOW groups. The survival differences were statistically assessed using the log-rank test, a widely used method to determine whether there is a significant difference in survival times between groups.

Kaplan-Meier curves were generated using survfit() and visualised using ggsurvplot() from the survminer package. The log-rank p-value was annotated in each plot.

Additional Validation of gSELECT with Different Datasets

To further validate and demonstrate the use of gSELECT, we performed gSELECT analyses for two additional datasets: one containing Smart-seq v4 data (GSE185862) and the "panc8" dataset of the R package SeuratData (https://github.com/satijalab/seurat-data), which consists of four human pancreatic islet cell datasets obtained using different technologies.

Data Processing and Differential Expression Analysis of Mouse Cortex Data

The Smart-seq v4 data was originally published by Yao et al. (2021), who analysed the taxonomy of transcriptomic cell types across the isocortex and hippocampal formation in mice [24], and is available via the GEO database (GSE185862). All analyses were performed in Python 3.11.2 under Windows 10 using the following libraries: scanpy[4] version 1.11.3, anndata [25]version 0.11.4, h5py [26] version, 3.14.0, pandas [6] version 2.2.2, NumPy [8] version 1.24.4, SciPy [7] version 1.15.3, Matplotlib [9] version 3.10.3, scrublet [5] version 0.2.3, and scikit-misc [27] version 0.5.1.

Raw expression data were obtained from the original .hdf5 matrix and joined with corresponding metadata (.csv.gz) based on standardised cell barcodes. Duplicate entries were removed, and missing labels were filled with "Unknown". A structured .h5ad object was constructed using anndata, including exon count matrices, gene symbols, and donor/sample annotations. UMAP coordinates were integrated from GSE185862_umap2d_ssv4.csv.gz (scripts also available at https://github.com/AC-PHD/gselect-singlecell-scripts).

Quality control was performed by calculating mitochondrial and ribosomal content (based on gene prefixes mt-, rpl, and rps) as well as total counts and detected genes per cell. Cells were retained if they had between 5,000 and 15,000 detected genes, total counts between 50,000 and 2,000,000, mitochondrial content <1%, and ribosomal content <20%. Gene-level filtering required a minimum of 3 cells expressing each gene. Potential doublets were identified using Scrublet (n=20,000 subset) with

an empirically determined threshold of 0.25 and removed from downstream analyses. Expression values were normalised to a total of 1,000,000 counts per cell and log-transformed (log1p).

For unsupervised representation, we applied principal component analysis (PCA) followed by uniform manifold approximation and projection (UMAP). Prior to dimensionality reduction, highly variable genes were selected (n=2000, Seurat flavour), and gene expression was scaled to unit variance (clipped at 10). Neighbourhood graphs were constructed using the first 30 PCs (n_neighbors=15). Sex-specific UMAP plots were generated with customised colour palettes and high-resolution export (600 dpi).

For differential gene expression (DEG) analysis, cells were filtered to compare female (F) vs. male (M) donors (donor_sex_label). Wilcoxon rank-sum tests and logistic regression were performed using scanpy.tl.rank_genes_groups() with default parameters. Global comparisons across all cells as well as stratified analyses within annotated cell types were conducted. For Wilcoxon, effect sizes (log2FC) and adjusted p-values (Benjamini–Hochberg FDR) were reported. For logistic regression, model scores and log2 fold changes were used to rank genes.

Top-ranked DEGs (n=20) were visualised using volcano plots and UMAP overlays. Barplots based on logistic regression scores were also used to display marker genes. All results were exported as .csv and .xlsx files.

The Wilcoxon test results were visualised via a volcano plot, showing log2 fold change against -log10 adjusted p-values (FDR). Genes were labelled as significant if $|\log2FC| > 1$ and $p\_val\_adj < 0.05$. The top 10 genes (smallest adjusted p-values) were annotated directly in the plot using dynamic text positioning. Logistic regression results were sorted by classifier weight, and the top 10 marker genes were visualised in a horizontal bar plot.

To visualise the expression of selected sex-linked genes (e.g. *Xist*, *Eif2s3y*, *Kdm5d*), raw counts (log1p-transformed) were plotted using violin plots (stratified by sex) with consistent axis styling, group order, and colour codes.

All plots were exported as high-resolution PNG files (600 dpi). No external normalisation or batch correction was applied, in line with the aim of evaluating predictive signals under minimally processed conditions.

Data Processing and Differential Expression Analysis of Multi-Batch Pancreas Data

To analyse the potential effects of batch effects, we also analysed the "panc8" dataset of the R package SeuratData [3] (version 0.2.2.9002, available at https://github.com/satijalab/seurat-data). As described in the main text, the non-integrated data shows clear batch effect (Supplementary Figure S 1A) and was subset according to cell type without batch effect correction.

Integration, in the sense of batch correction, was performed using Seurat's IntegrateLayers() function following the recommended workflow [28, 29]. This procedure harmonizes shared cell types across datasets and reduces technical variation. The integrated data (Supplementary Figure S 1B) was subsequently subset by cell type for downstream analysis.

The analysis of the MI genes identified using the integrated data is discussed in detail in the main text.

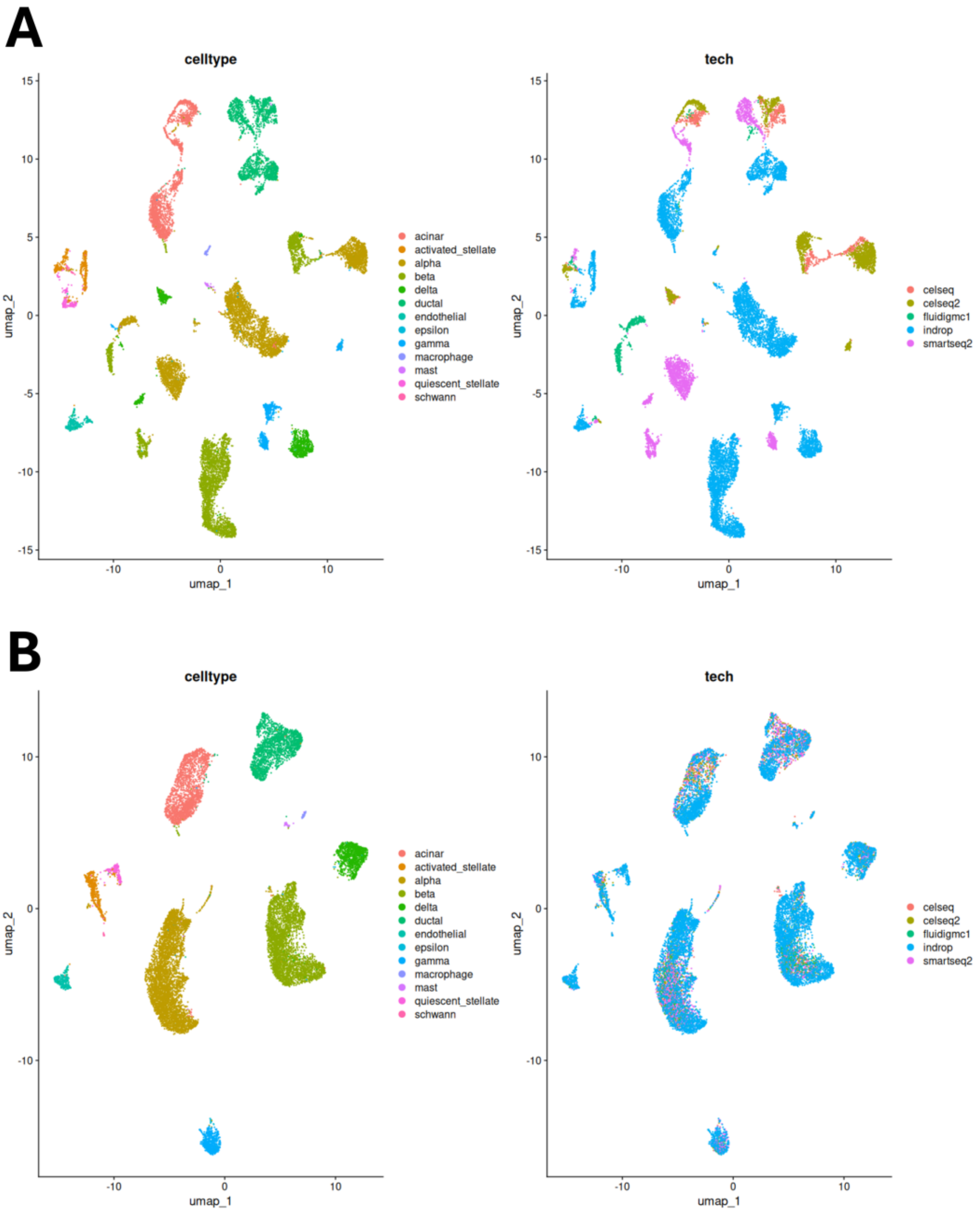

Supplementary Figure S 1: Visualisation of the "panc8" dataset before and after integration. ***(A)*** *Before integration, grouping the panc8 data by technology results in a batch effect.* ***(B)*** *Integrating the datasets using Seurat's IntegrateLayers() function removes the batch effect.*

Subsequently, data preparation was performed for integrated and non-integrated alpha and beta cells. Due to the data being available as Seurat v5 object with layers for each technique, the original dataset preparation code was slightly adjusted, and Seurat version 5.3.0 was used [12, 13, 16, 17], and ggplot2 [19] version 3.5.2 was used for visualisation. In newer Seurat versions, the location of the counts within

the Seurat object has changed. Additionally, the count data of each technology was saved in a separate layer. We obtained the respective count data of the data (“Input_RDS”) via:

```
for (layer in count layers) {
    counts <- Input_RDS@assays$RNA@layers[[layer]]
    tech_name <- gsub("counts\\.", "", layer)
    cell_names <- WhichCells(Input_RDS, expression = tech == tech_name)
    # Check 1: number of rows and colums
  num_cells <- length(cell_names)
  num_columns <- ncol(counts)
    if (num_cells == num_columns) {
    cat(paste0(tech_name, ": ", num_cells, " cell names found and ", num_columns, " columns in the count layer -> OK\n"))
  } else {
    cat(paste0(tech_name, ": number of cell names (", num_cells, ") DOES NOT FIT to the number of columns in the count layer (", num_columns, ")!\n"))
    stop("Error: cell names and number of columns DO NOT MATCH, please check your data!")
  }

# Cell names, gene names, save as matrix
  colnames(counts) <- paste0(tech_name, "_", cell_names)
  rownames(counts) <- gene_names
  count_matrices[[tech_name]] <- as.matrix(counts)
}
```

The resulting count matrices for the different technologies were combined

```
combined_counts <- do.call(cbind, count_matrices)
```

and saved as data.frame

```
final_df <- cbind(Gene = gene_names, as.data.frame(combined_counts))
```

The data.frame was subsequently used as the “Counts_DF” data.frame of the original code

```
Counts_DF <- final_df
```

and the remaining steps of the data preparation were performed as previously described [11, 30] according to the data preparation steps available via GitHub (https://github.com/AC-PHD/Seurat_PFA_pipeline).

Supplementary Results for the Analysis of the Multi-Batch Pancreas Data

To validate the resulting MI genes, we analysed the data with the FindMarkers() function of the Seurat package, using three different methods: ‘wilcox’, for the Wilcoxon rank sum test (default in Seurat), ‘MAST’, which uses the MAST package [22] for differential expression testing, and ‘roc’, which employs ROC analysis and evaluates a classifier for each gene (using AUC), to classify between two groups, ranking the genes according to their ‘predictive power’ (Supplementary Figure S 2).

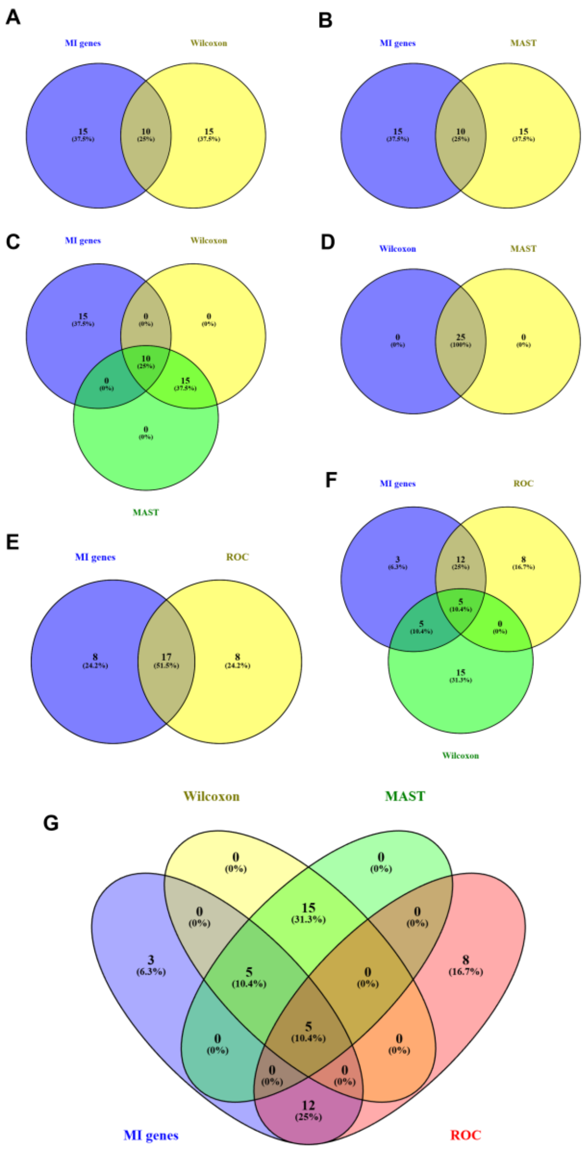

Supplementary Figure S 2: Comparison of the 25 top-ranked MI genes and the 25 top-ranked genes identified using the Seurat function FindMarkers() with different methods based on integrated data of the ‘panc8’ dataset from SeuratData. ***(A)*** *MI genes identified using integrated data in gSELECT (blue ellipse) compared with genes identified with Seurat’s*

FindMarkers() function using 'wilcox' as method (yellow ellipse). ***(B)*** *MI genes identified using integrated data in gSELECT (blue ellipse) compared with genes identified with Seurat's FindMarkers() function using 'MAST' as method (yellow ellipse).* ***(C)*** *Using FindMarkers() with 'wilcox' (yellow ellipse) and 'MAST' (green ellipse) identified the same genes, 10 of these genes were also identified by gSELECT (MI genes, blue ellipse).* ***(D)*** *Using FindMarkers() with 'wilcox' (blue ellipse) and 'MAST' (yellow ellipse) identified the same genes.* ***(E)*** *MI genes identified using integrated data in gSELECT (blue ellipse) compared with genes identified with Seurat's FindMarkers() function using 'roc' as method (yellow ellipse).* ***(F)*** *17 genes were identified by both, gSELECT and ROC, and 10 genes were identified by gSELECT and Wilcoxon rank sum test, five genes were identified by all three methods.* ***(G)*** *12 genes were identified by gSELECT and ROC, but not by Wilcoxon and MAST analysis, while five genes were identified by gSELECT, Wilcoxon and MAST analysis, but not by ROC, and five genes were identified by all four methods.*

Despite the UMAP visualisation showing a batch effect (Supplementary Figure S 1, and Supplementary Figure S 3F), both gSELECT and Seurat FindMarkers() identified the same genes for integrated and not-integrated data (Supplementary Figure S 3A-E). The differences appear to affect only the visual representation (UMAP), not the marker gene selection via FindMarkers() (in Seurat version 5.3.0) nor the identification of MI genes. While 'wilcox' and 'MAST' had ten genes in common with the MI genes, 17 of the MI genes were also identified when using 'roc' as test method. Again there were no differences between the integrated and the non-integrated panc8 data (Supplementary Figure S 3G) for the Seurat FindMarkers() analysis and for the MI genes (Supplementary Table S 1). A reason could be that the batches are associated with prevalent phenotypic differences that drive the UMAP clustering. Similar to our experiment in the main paper where the expression data has hidden information (information not obvious in the UMAP visualisation) about the sex of the mice that do not seem to be dominant compared to other differences, there could be hidden information about the differences of interest that yet does not drive the UMAP clustering. However, gSELECT is able to identify relevant genes even in such cases.

Supplementary Table S 1: *Comparison of the mutual information values for the top-ranked MI genes resulting from integrated and not integrated data.*

Integrated (without batch effect)		Not Integrated (with batch effect)	
gene_name	mutual information	gene_name	mutual information
INS	0.7736630530409307	INS	0.7736630530409307
GCG	0.6932520578584919	GCG	0.6932520578584919
GC	0.5679749620269819	GC	0.5679749620269819
IAPP	0.5580769074269463	IAPP	0.5580769074269463
IRX2	0.5562227035039561	IRX2	0.5562227035039561
ADCYAP1	0.5099718783087857	ADCYAP1	0.5099718783087857
TTR	0.4888938733184024	TTR	0.4888938733184024
TM4SF4	0.4806005916916795	TM4SF4	0.4806005916916795
HADH	0.470991519959492	HADH	0.470991519959492
CLU	0.4258275563628394	CLU	0.4258275563628394

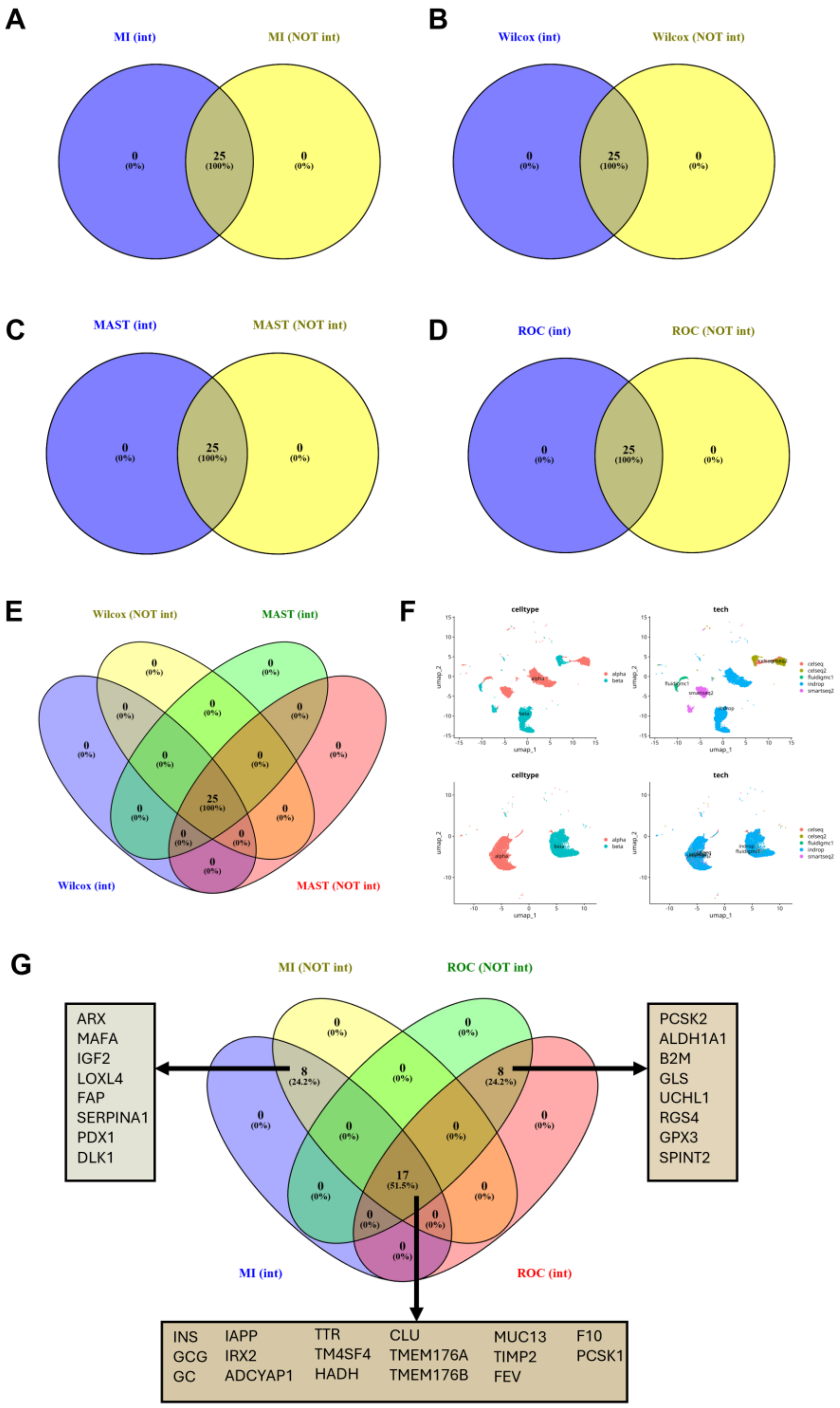

Supplementary Figure S 3: Integration appears to affect the batch effect in the UMAP visualisation but not the genes identified by gSELECT or FindMarkers(). *(**A**) MI genes identified by gSELECT using integrated data (blue ellipse) and data before integration (not integrated, yellow ellipse). (**B**) Genes identified by Seurat's FindMarkers() function using 'wilcox' and integrated data (blue ellipse) and data before integration (not integrated, yellow ellipse). (**C**) Genes identified by Seurat's FindMarkers() function using 'MAST' and integrated data (blue ellipse) and data before integration (not integrated, yellow ellipse). (**D**) Genes identified by Seurat's FindMarkers() function using 'roc' and integrated data (blue ellipse) and data before integration (not integrated, yellow ellipse). (**E**) The FindMarkers() methods 'wilcox' and 'MAST' identified the same genes for integrated and not integrated data. (**F**) The non-integrated data for the subset of alpha and beta cells is showing a clear batch effect (top), which was corrected by data integration (bottom). (**G**) Among the 25 top-ranked genes, eight genes were only identified with gSELECT but not with FindMarkers() using 'roc', and eight genes were only identified with with FindMarkers() using 'roc' but not with gSELECT, 17 genes were identified by both methods.*

Additional Information on the MI Genes for the Comparison between Pancreatic Alpha and Beta Cells

When comparing alpha and beta cells, which are both endocrine cell types of the islets of Langerhans [31], in the "panc8" multi batch human pancreatic islet cell dataset, gSELECT identified *INS*, *GCG*, *GC*, *IAPP*, *IRX2*, *ADCYAP1*, *TTR*, *TM4SF4*, *HADH*, *CLU*, *TMEM176A*, *TMEM176B*, *MUC13*, *ARX*, *TIMP2*, *FEV*, *F10*, *MAFA*, *PCSK1*, and *IGF2* as the 20 top-ranked MI genes.

The top ranked MI gene, *INS* (Insulin), can be used to identify beta cells, which secrete insulin [31-34], and the second ranked MI gene, *GCG* (glucagon), can be used to identify alpha cells, which secrete glucagon [31-34]. The endocrine islets, which are also compromised of at least three other cell types besides alpha and beta cells, play an essential role in glucose homeostasis [31]. Loss of functional beta cells characterises the development of diabetes, and type 2 diabetes is known to be caused by dysfunction or a reduced mass of beta cells in combination with increased INS resistance in peripheral tissues [31].

Additionally, several other MI genes have been associated with alpha or beta cell-specific expression, such as *IRX2* [31, 33-36], *ARX* [31, 32, 35, 36], *GC* [31], which have been associated with alpha cells, and *IAPP* [31-36], *HADH* [33-36], *MAFA* [31-36], *ADCYAP1* [31-34], which have been associated with beta cells. Almost all of the MI genes have been associated with alpha or beta cells in the human islet cell type-specific identity gene sets by van Gurp et al. (2022) [33, 34]. Other studies link the MI genes to biological functions. For instance, alpha and beta cell-related dysfunction in the processing of pro-GCG to GCG and pro-IAPP to IAPP, respectively, has been reported to correlate with increasing body mass index [31], which might indicate a possible biological importance of the two MI genes.

Supplementary Table S 2: *The 20 top-ranked MI genes and their associations with alpha and beta pancreatic islet cells in the literature.*

Gene	Information
INS<br>insulin	Specific marker for beta cells [31], expression reported as specific for beta cells [32], in *GCG*/*INS* bi-hormonal cells located in pancreatic alpha cell clusters, upregulation of key genes associated with beta cell identity, such as *INS* and *IAPP*, has been observed [33, 34], part of the beta cell gene set by van Gurp et al. (2022) [33, 34]
GCG<br>glucagon	Specific marker for alpha cells [31], dysfunction in the processing of pro-GCG to GCG has been reported to correlate with increasing body mass index [31], expression reported as specific for alpha cells [32], In *GCG*/*INS* bi-hormonal cells located in pancreatic beta cell clusters, upregulation of key genes associated with alpha cell identity, such as *GCG*, *ARX*, *TM4SF4* and *FEV*, has been observed [33, 34], part of the alpha cell gene set by van Gurp et al. (2022) [33, 34]
GC<br>group-specific component (vitamin D binding protein)	Known marker for alpha cells [31], shared marker for alpha and gamma cells [33, 34], part of the alpha cell gene set by van Gurp et al. (2022) [33, 34],
IAPP<br>Islet amyloid polypeptide	Known as beta cell-specific expression factor [35, 36], elevated expression has been reported in beta cells [31], dysfunction in the processing of pro-IAPP to IAPP has been reported to correlate with increasing body mass index [31], expression reported as specific for beta cells [32], in *GCG*/*INS* bi-hormonal cells located in pancreatic alpha cell clusters, upregulation of key genes associated with beta cell identity, such as *INS* and *IAPP*, has been observed [33, 34], part of the beta cell gene set by van Gurp et al. (2022) [33, 34]
IRX2<br>Iroquois-class homeodomain protein IRX-2	Known as transcription factor contributing to alpha cell fate specification [35, 36], has been suggested as potential inhibition target for promoting beta cell identity [35, 36], transcription factor in alpha cells [33, 34], part of the alpha cell gene set by van Gurp et al. (2022) [33, 34]
ADCYAP1<br>Pituitary adenylate cyclase-activating polypeptide	Expression reported as specific for beta cells [32], elevated expression in beta cells [31], in beta cells involved in maintaining glycemia [33, 34], part of the beta cell gene set by van Gurp et al. (2022) [33, 34]
TTR<br>Transthyretin	Expressed in alpha and beta cells [37], has been reported as expressed in alpha cells, and it has been suggested that *TTR* expression in pancreatic alpha cells might be involved in glucose homeostasis by regulating glucagon expression [37], Known as functional protein of beta cells, protects against beta-cell death and promotes insulin release [38], in diabetic patients, significantly lower TTR plasma levels have been reported [37], part of the alpha cell gene set by van Gurp et al. (2022) [33, 34]
TM4SF4	In *GCG*/*INS* bi-hormonal cells located in pancreatic beta cell clusters, upregulation of key genes associated with alpha cell identity, such as *GCG*, *ARX*, *TM4SF4* and *FEV*, has been observed [33, 34], cell surface marker in alpha cells [33, 34], shared marker for alpha and gamma cells according to van Gurp et al. (2022) [33, 34], part of the alpha cell gene set by van Gurp et al. (2022) [33, 34]
HADH	Known as beta cell-specific expression factor [35, 36], in beta cells involved in maintaining glycemia [33, 34], shared marker for beta and delta cells according to van Gurp et al. (2022) [33, 34], part of the beta cell gene set by van Gurp et al. (2022) [33, 34]

CLU	part of the alpha cell gene set by van Gurp et al. (2022) [33, 34]
TMEM176A	part of the alpha cell gene set by van Gurp et al. (2022) [33, 34]
TMEM176B	shared marker for alpha and gamma cells according to van Gurp et al. (2022) [33, 34], part of the alpha cell gene set by van Gurp et al. (2022) [33, 34]
MUC13<br>Mucin 13, cell surface associated	part of the alpha cell gene set by van Gurp et al. (2022) [33, 34]
ARX<br>Aristaless related homeobox	Known as transcription factor contributing to alpha cell fate specification [35, 36], has been observed as expressed in alpha and gamma cells [31], expression reported as specific for alpha cells [32], In *GCG*/*INS* bi-hormonal cells located in pancreatic beta cell clusters, upregulation of key genes associated with alpha cell identity, such as *GCG*, *ARX*, *TM4SF4* and *FEV*, has been observed [33, 34],
TIMP2	shared marker for beta and delta cells according to van Gurp et al. (2022) [33, 34], part of the beta cell gene set by van Gurp et al. (2022) [33, 34]
FEV	In *GCG*/*INS* bi-hormonal cells located in pancreatic beta cell clusters, upregulation of key genes associated with alpha cell identity, such as *GCG*, *ARX*, *TM4SF4* and *FEV*, has been observed [33, 34]
F10<br>Coagulation factor X	part of the alpha cell gene set by van Gurp et al. (2022) [33, 34]
MAFA	Expression has been reported as restricted to beta cells [35, 36], elevated expression has been reported in beta cells [31], known to regulate insulin expression [35, 36], known as key identity regulator for beta cells according to van Gurp et al. (2022) [33, 34], part of the beta cell gene set by van Gurp et al. (2022) [33, 34]
PCSK1	PCSK1 is known to convert pro-hormones to active hormones, with INS being among its substrates [31], altered function of PCSK1 has been associated with obesity in mice and humans [31], shared marker for beta and delta cells according to van Gurp et al. (2022) [33, 34], part of the beta cell gene set by van Gurp et al. (2022) [33, 34]
IGF2<br>Insulin-like growth factor 2 (somatomedin A)	part of the beta cell gene set by van Gurp et al. (2022) [33, 34]

Case Study Using a Lung Cancer Cell Line

The lung cancer cell data published by Xue et al. (2020) as GSE137912 [39], which was also employed to demonstrate the scenario of "no differences between the two groups", contains single-cell sequencing data of H358 cells, which were treated with the KRASG12C inhibitor ARS-1620 for 0 h, 4 h, 24 h, and 72 h [39]. Besides the scenario of no differences between the two groups, which is described in the main text, the single-cell sequencing data of H358 cells also offers the opportunity to analyse two rather similar groups according to UMAP visualisation (untreated H358 cells compared to H358 cells after 4 h of ARS-1620 treatment), and two clearly different groups according to UMAP visualisation (untreated H358 cells compared to H358 cells after 72 h of ARS-1620 treatment). In the following, we describe the respective analyses as a case study and validate the resulting genes of interest using various standard methods.

Overview of the Case Study Analysis Pipeline

gSELECT was used to analyse H358 cells treated with ARS-1620, comparing the different conditions, untreated (0 h), 4 h, and 72 h, to evaluate its ability to highlight potentially relevant genes before downstream analysis regarding the changes in gene expression under treatment. The data allow analysing three different scenarios: (1) no differences between the two groups, which is discussed in detail in the main text, (2) two rather similar groups according to UMAP visualisation, which is demonstrated in this case study using untreated H358 cells and H358 cells after 4 h of ARS-1620 treatment, and (3) two clearly different groups, which is demonstrated in this case study using untreated H358 cells and H358 cells after 72 h of ARS-1620 treatment.

The workflow is as follows: We first applied our tool to identify genes with high mutual information with regard to the cell type (represented by the label). These genes were then validated by standard differential expression methods, including Wilcoxon, MAST [22], DESeq2 [21], which are available via the R-package Seurat [12, 16-18, 40], as well as further validation using scClassify [41] and Python-based standard analysis methods such as scanpy's rank_genes_groups [4] and AUROC in Python using

scikit-learn [42] and via Seurat's FindMarkers() function, applied on the third scenario. To gain further insights into potential biological relevance, we examined whether the top-ranked MI genes showed known functional associations using the STRING database [43-45]. Additionally, we assessed the predictive power of these genes in classification tasks to determine their ability to distinguish between treatment conditions. Finally, to assess whether genes highlighted in the pre-analysis step have clinical relevance, we examined their expression in patient data from the TCGA database [23]. We performed survival analysis to explore potential associations with prognosis, which also highlights potential starting points for a causal investigation to analyse if the expression values of the selected genes under treatment might be related to therapy success.

Second Scenario: Two Rather Similar Groups according to their UMAP-visualisation

For this scenario in the case study, we compared untreated H358 cells and H358 cells after 4 h of ARS-1620 treatment, which show no clear separation between the two groups in the UMAP visualisation (Supplementary Figure S 4A) regarding our labels of interest. As no distinct differences are discernible in the UMAP visualisation, determining the value of further analysis is challenging.

As in the main text, the first analysis step is to perform a gSELECT analysis using all available non-constant genes. For untreated H358 cells and H358 cells after 4 h of ARS-1620 treatment, this analysis resulted in a high balanced accuracy (Supplementary Figure S 4C) and a small number of misclassified samples (Supplementary Figure S 4D, a mean of 8.50 misclassified samples (with standard deviation of 1.63) out of a total number of 2251 cells (1057 untreated cells, 1194 cells after 4 h of ARS-1620 treatment, which are here referred to as samples)). This indicates that the data could contain information about the differences between the two groups, despite the overlapping clusters observed in the UMAP visualisation. The MI genes are calculated based on raw data, on normalized genes (Scanpy's sc.pp.normalize_total(), sc.pp.log1p()), meaning normalizing each total cell count over all genes and then transforming the count per gene over all cells with a log-transformation, and on highly variable genes (Scanpy's sc.pp.highly_variable_genes). While the MI calculation for normalised and highly

variable genes resulted in a 100% concordance (Supplementary Figure S 4B), there is a deviation with the MI genes based on the raw data. As invertible functions like normalizations over the features (genes) do not change our MI calculation as the discretization routine bins the data points according to their relative order independent of absolute values, the issue is that normalization takes place also within one sample, which is the count normalization in a cell. At this step, it can happen that the relative position of a cell (data point) regarding a particular gene compared with other cells can shift and thus ends up in a different discretized bin compared to the raw data, which can lead to a different MI value for a particular gene calculated over all samples (cells). Subsequent analyses were performed using normalized genes. However, it is also possible to perform the analyses with raw data, highly variable genes or even custom groups.

The second step of the gSELECT analysis workflow is to calculate the accuracy of the top-ranked MI genes, which is visualised in Supplementary Figure S 4E. Besides considering all available genes to differentiate between the two groups, gSELECT can also calculate the accuracy of the top-ranked MI genes or different subgroups of the top-ranked MI genes. Supplementary Figure S 4E visualises the correlation between the number of top-ranked MI genes (e.g., the top 5, the top 10, the top 15, …) and the balanced accuracy resulting from only considering these genes regarding their predictive power. The accuracy of analysing the first top-ranked MI gene alone is slightly above 85%. Analysing either the ten or 25 highest-ranked MI genes achieves an accuracy exceeding 95%, comparable to the predictive accuracy obtained when analysing the complete set of over 25,000 genes. This suggests that a subset of the top-ranked MI genes can be as effective for differentiating between the two groups as the full gene set. A UMAP visualisation of the two groups considering only the ten top-ranked MI genes (Supplementary Figure S 4F) also indicates their predictive power of the MI genes, as the two groups are clearly separated when the UMAP visualisation is created using only the 10 top-ranked MI genes. The reason for this observation is that UMAP optimizes the projection of cells into the plane such that genes according to their expression profile that are close in the high dimensional space are close in the plane and similar for those cells that are distant. Consequently, the cells are arranged according to this objective not taking the specific differences into account. With gSELECT, we can provide a difference-aware or context-aware reduction of dimensions that focuses on dimensions (genes) that are associated

with the differences of interest. Consequently, in the smaller dimensional space based on the selected genes, the distances between cells rather coincide with the difference of interest, which could be diluted by other differences that are also present in the total data set with all dimensions (genes) and might represent also other markers or labels that we are not aware of. In contrast to the dimension reduction technique PCA that reduces dimensions according to variance in the data, which does not necessarily coincide with important information to separate the phenotypic observation of interest, we thus inform the dimension reduction with our intent of separating specific phenotypes and infuse relevant information into the dimension reduction process. For further validation, we also analysed the data using Seurat's FindMarkers() function with MAST, DESeq2, and Wilcoxon as test methods, ranking the resulting genes based on their absolute log2 fold changes and adjusted p-values of less than 0.05. The ten top-ranked genes were compared using a Venn diagram (Supplementary Figure S 4G), with eight genes being present in all four groups. This indicates the reliability of the ML algorithm and the resulting MI genes. The STRING [43, 46] analysis (version 12.0, analysed with medium confidence) of the 10 top-ranked MI genes shows that most of the top-ranked genes are also strongly interconnected (Supplementary Figure S 4H).

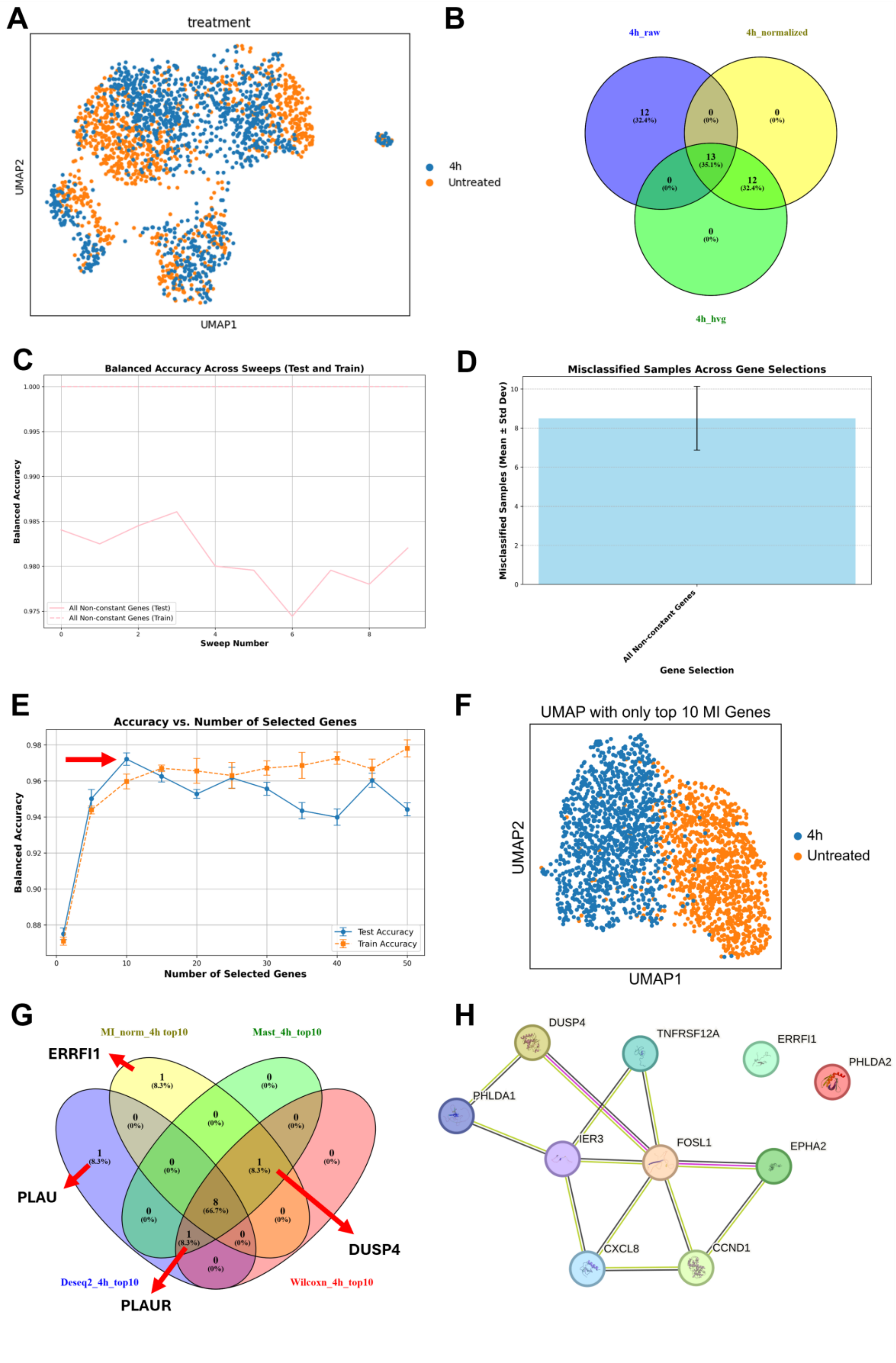

Supplementary Figure S 4: Results for the analysis of two rather similar groups (scenario 2). (A) *UMAP visualisation of untreated H358 cells and H358 cells after 4 h of ARS-1620 treatment.* ***(B)*** *Venn diagram of the MI genes resulting from analysing raw data, normalized genes and highly variable genes.* ***(C)*** *Balanced accuracy across sweeps for all available non-constant genes.* ***(D)*** *Number of misclassified samples for all non-constant genes (a mean of 8.50 misclassified samples (with a*

standard deviation of 1.63) out of a total number of 2251 cells (1057 untreated cells, 1194 cells after 4 h of ARS-1620 treatment, here referred to as samples)). ***(E)*** *Dependency between the number of selected MI genes and balanced accuracy for training (orange line) and test (blue line).* ***(F)*** *UMAP visualisation of the two groups when only considering the 10 top-ranked MI genes.* ***(G)*** *Venn diagram visualising the overlap between the 10 top-ranked MI genes (yellow ellipse), and the 10 top-ranked genes according to several standard analysis methods (DESeq2 (blue ellipse), MAST (green ellipse), and Wilcoxon (red ellipse)) via Seurat.* ***(H)*** *STRING analysis of the 10 top-ranked MI genes.*

Additionally, most of the MI genes are also identified as differentially expressed genes when using other methods, although the ranking of these genes might differ between the different methods. We remark that differences could come from the following fact that a log2 fold change is not that big, but the gene contains important information about the differences. Conversely, although having a high log2 fold change, the explanative power of a gene can be small compared to a gene with s small log2 fold change as the size of the log2 fold change might not always correlate with information content to explain an observed difference. Analogously, for any other measure, such as test statistics of corresponding statistical tests.

Third Scenario: Two Clearly Different Groups

When comparing untreated H358 cells to H358 cells after 72 h of ARS-1620 treatment, the two groups are clearly separated in the UMAP visualisation (Supplementary Figure S 5A). As in scenario 2, the comparison between raw data, normalized MI genes and highly variable MI genes (Supplementary Figure S 5B) indicates that normalized is a suitable option for the subsequent analyses since all of the genes overlap with the highly variable genes.

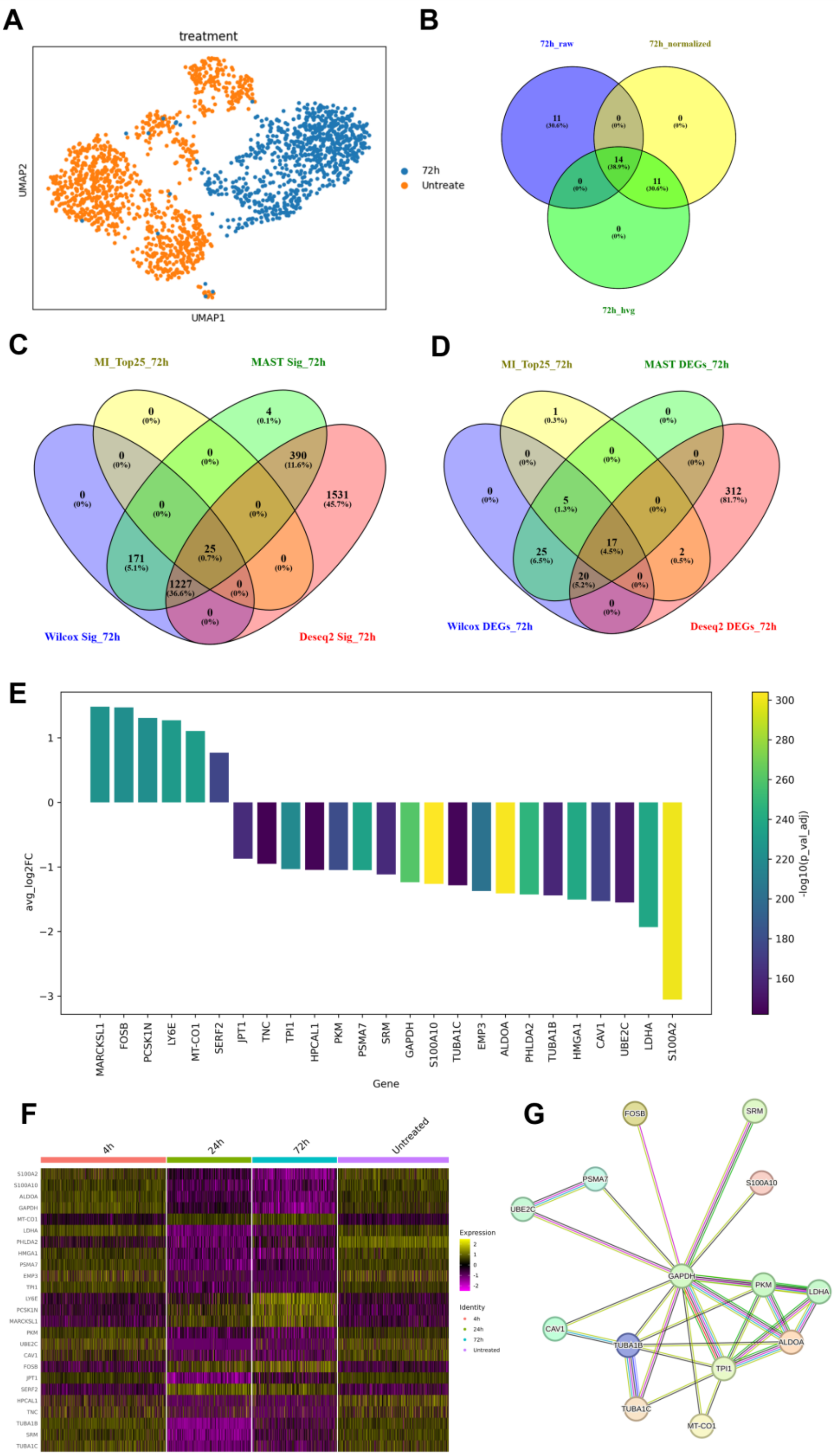

Supplementary Figure S 5: Results for the analysis of two clearly different groups (scenario 3). (A) *UMAP visualisation of untreated H358 cells and H358 cells after 72 h of ARS-1620 treatment.* ***(B)*** *Venn diagram of the MI genes resulting from analysing raw data, normalized genes and highly variable genes.* ***(C)*** *Venn diagram visualising the overlap between the top-ranked MI genes after 72 h of ARS-1620 treatment (yellow ellipse), and the significant genes according to several standard analysis methods (Wilcoxon (blue ellipse), MAST (green ellipse), and DESeq2 (red ellipse)) via Seurat.* ***(D)*** *Venn diagram*

visualising the overlap between the top-ranked MI genes after 72 h of ARS-1620 treatment (yellow ellipse), and the differentially expressed genes according to several standard analysis methods (Wilcoxon (blue ellipse), MAST (green ellipse), and DESeq2 (red ellipse)) via Seurat. ***(E)*** *Visualisation of the changes in gene expression for the 25 top-ranked MI genes according to MAST analysis. A positive log2 fold change indicates upregulation in H358 cells after 72 h of ARS-1620 treatment, and a negative log2 fold change downregulation.* ***(F)*** *Heatmap visualising the gene expression of the 25 top-ranked MI genes after 4 h, 24 h, and 72 h of ARS-1620 treatment and in untreated H358 cells.* ***(G)*** *STRING analysis of the 25 top-ranked MI genes.*

Comparing the 25 top-ranked MI genes with the analysis results of three standard methods MAST, DESeq2, and Wilcoxon (Supplementary Figure S 5C and Supplementary Figure S 5D) also indicates the relevance of the 25 top-ranked MI genes, as all of the 25 top-ranked MI genes are among the significant genes obtained with other standard analyses methods (Supplementary Figure S 5C).

17 of the top-ranked MI genes (*S100A2*, *LDHA*, *UBE2C*, *CAV1*, *HMGA1*, *TUBA1B*, *PHLDA2*, *ALDOA*, *EMP3*, *TUBA1C*, *S100A10*, *GAPDH*, *SRM*, *PSMA7*, *PKM*, *HPCAL1*, and *TPI1*) are also differentially expressed according to all three standard analysis methods (Supplementary Figure S 5D). Additionally, one of the 25 top-ranked MI genes (*SERF2*) was highly significant in all analyses but not among the differentially expressed genes, five of the MI genes were among the differentially expressed genes in Wilcoxon and MAST analysis (*MARCKSL1*, *FOSB*, *PCSK1N*, *LY6E*, *MT-CO1*). Two MI genes (*JPT1* and *TNC*) were among the differentially expressed genes in DESeq2. Supplementary Figure S 5E visualises the differences in gene expression for the 25 top-ranked MI genes, using the calculated log2 fold changes and adjusted p-values of the MAST analysis. The differences in gene expression after 4 h, 24 h, and 72 h of ARS-1620 treatment and untreated H358 cells are also shown as a heatmap in Supplementary Figure S 5F. While the top 25 MI genes identified at 72 h show minimal expression differences between 4 h and untreated samples, a much stronger effect is evident at 24 h and 72 h. Interestingly, the 25 top-ranked MI genes of a total of over 25,000 genes form a highly interconnected network (Supplementary Figure S 5G, STRING [43, 46] analysis with medium confidence and disconnected nodes hidden).

The predictive power of the MI genes can be evaluated using the balanced accuracy, with higher values for the balanced accuracy indicating a better predictive power. Corresponding to the clear separation between untreated H358 cells to H358 cells after 72 h of ARS-1620 treatment in the UMAP visualisation in Supplementary Figure S 5A, the balanced accuracy of all non-constant genes is almost 99.99%

(Supplementary Figure S 6A), with a mean of 2.30 misclassified samples out of a total number of 1864 cells (1057 untreated cells and 807 cells after 72 h of ARS-1620 treatment, with a standard deviation of 0.46, Supplementary Figure S 6B). The top-ranked MI gene (S100A2) alone is sufficient for a balanced accuracy of around 85%. With 10 MI genes, the predictive power increases to more than 96% and with the 25 top-ranked MI genes, the predictive power is more than 98% (Supplementary Figure S 6C). This high predictive power is achieved with only 25 MI genes instead of all 25,000 genes. At the same time, less than 10 samples get misclassified when using more than 10 MI genes (Supplementary Figure S 6D).

The comparison of the predictive power of five MI genes (blue) and five random genes (green) in Supplementary Figure S 6E demonstrates the consistently high predictive power of the MI genes. In contrast, the predictive power of the same number of random genes is around 60%, with some sweeps achieving a predictive power of less than 60% while other sweeps resulted in a predictive power of around 70%. Only one sweep with the random genes resulted in a predictive power of more than 80%, indicating that this relatively high accuracy was likely due to chance. The number of misclassified samples is also significantly lower for the MI genes than for the same number of random genes (Supplementary Figure S 6F). While fewer than 20 samples are misclassified when using the 5 top-ranked MI genes, the same number of random genes results in more than 120 misclassifications.

Besides identifying potentially relevant genes with another analysis method (mutual information), gSELECT can also calculate the predictive power of custom gene combinations, which is one of the – to the best of our knowledge – novel functions of gSELECT. These genes can be top-ranked MI genes but can also be other genes such as genes of interest from literature research, provided the genes are available in the dataset. Here, we demonstrate this function using the five top-ranked MI genes. Figure 8G showcases the predictive power of the ten top-ranked gene combinations (out of 31 possible combinations) for the top-five MI genes. As mentioned in the Methods section and the Tutorial, analysing a high number of genes with this explorative function can require a significant amount of time and computational power, as it follows a $2^n - 1$ growth pattern where n represents the number of genes.

Therefore, gSELECT automatically switches to a greedy algorithm when calculating the predictive power of more than ten genes by default. This default setting can be adjusted; however, we strongly recommend limiting the analysis to a maximum of ten genes because of the $2^n - 1$ growth pattern. Even adding just one more gene than in our example (resulting in a total of six genes) will result in 63 possible combinations, which will require more than twice the calculation time required for five genes.

As described in the main text, we also analysed the predictive power of different combinations of the five top-ranked MI genes (Supplementary Figure S 6G). This analysis uses one of the novel functions of gSELECT, testing the predictive power of all possible combinations of up to five genes. Here we demonstrate the use of this function on the five top-ranked MI genes in the comparison between H358 cells after 0 h and 72 h of ARS-1620 treatment, *S100A2*, *S100A10*, *ALDOA*, *GAPDH*, and *MT-CO1*. The ten top-ranked subsets (out of 31 possible combinations that were calculated) are shown in Supplementary Figure S 6G.

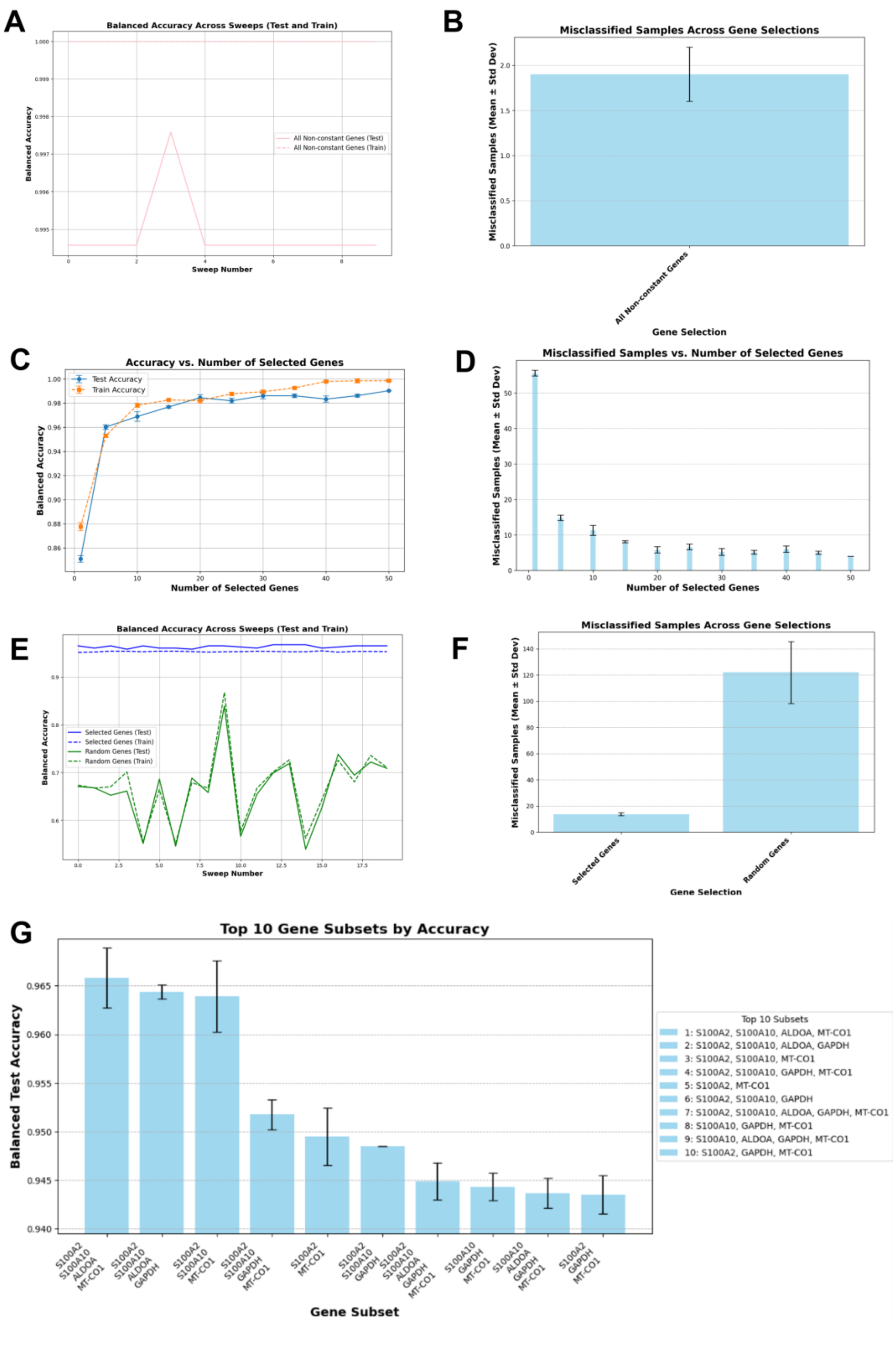

Supplementary Figure S 6: Balanced accuracy for different numbers of MI genes after 72 h of ARS-1620 treatment (A) *Balanced accuracy across sweeps for all available non-constant genes.* ***(B)*** *Number of misclassified samples for all non-constant genes (a mean of 2.30 misclassified samples (with standard deviation of 0.46) out of a total number of 1864 cells (1057 untreated cells and 807 cells after 72 h of ARS-1620 treatment, here referred to as samples).* ***(C)*** *Dependency between the number of selected MI genes and balanced accuracy for training (orange line) and test (blue line).* ***(D)*** *Dependency between the number of selected MI genes and the number of misclassified samples.* ***(E)*** *Comparison of the predictive power of the five top-ranked MI-genes (blue lines) and five random genes (green lines) in training (dashed lines) and test (solid lines).* ***(F)*** *Number of misclassified samples for the five top-ranked MI genes compared to the number of misclassified samples using five random genes.* ***(G)*** *Predictive power of different MI gene combinations, using different subsets of the 10 top-ranked MI genes.*

Top-ranked Mutual Information Genes: Validation by Enrichment Analysis

As visualised in the figures above, the MI genes are highly significant, highly interconnected, and also confirmed by other standard analysis methods, such as Wilcoxon, DESeq2 and MAST, which are available via the Seurat package (in R) [12, 16-18, 40]. For further validation, we performed enrichment analyses with the 25 top-ranked MI genes to see which pathways are associated with the respective genes. Glycolysis is among the most significantly enriched Hallmarks (Supplementary Figure S 7A). The genes involved in Glycolysis and the other significant Hallmarks are visualised as a CNET plot in Supplementary Figure S 7B, using the log2 fold changes of the MAST analysis.

As glycolysis plays an important role in cancer metabolism and is linked to the Warburg effect, we decided to analyse the top MI genes connected to the glycolysis pathway to showcase the gSELECT function for analysing custom genes of interest. To analyse the predictive power of the four top-ranked MI genes associated with glycolysis, *PKM*, *ALDOA*, *TPI1*, and *LDHA*, we calculated the balanced accuracy of these genes using the function for calculating the predictive power of custom genes, which is, to the best of our knowledge, also a novel function unique to gSELECT. With more than 90%, the balanced accuracy of the four MI genes associated with glycolysis is significantly higher than the predictive power of an equal number of random genes (Supplementary Figure S 7C).

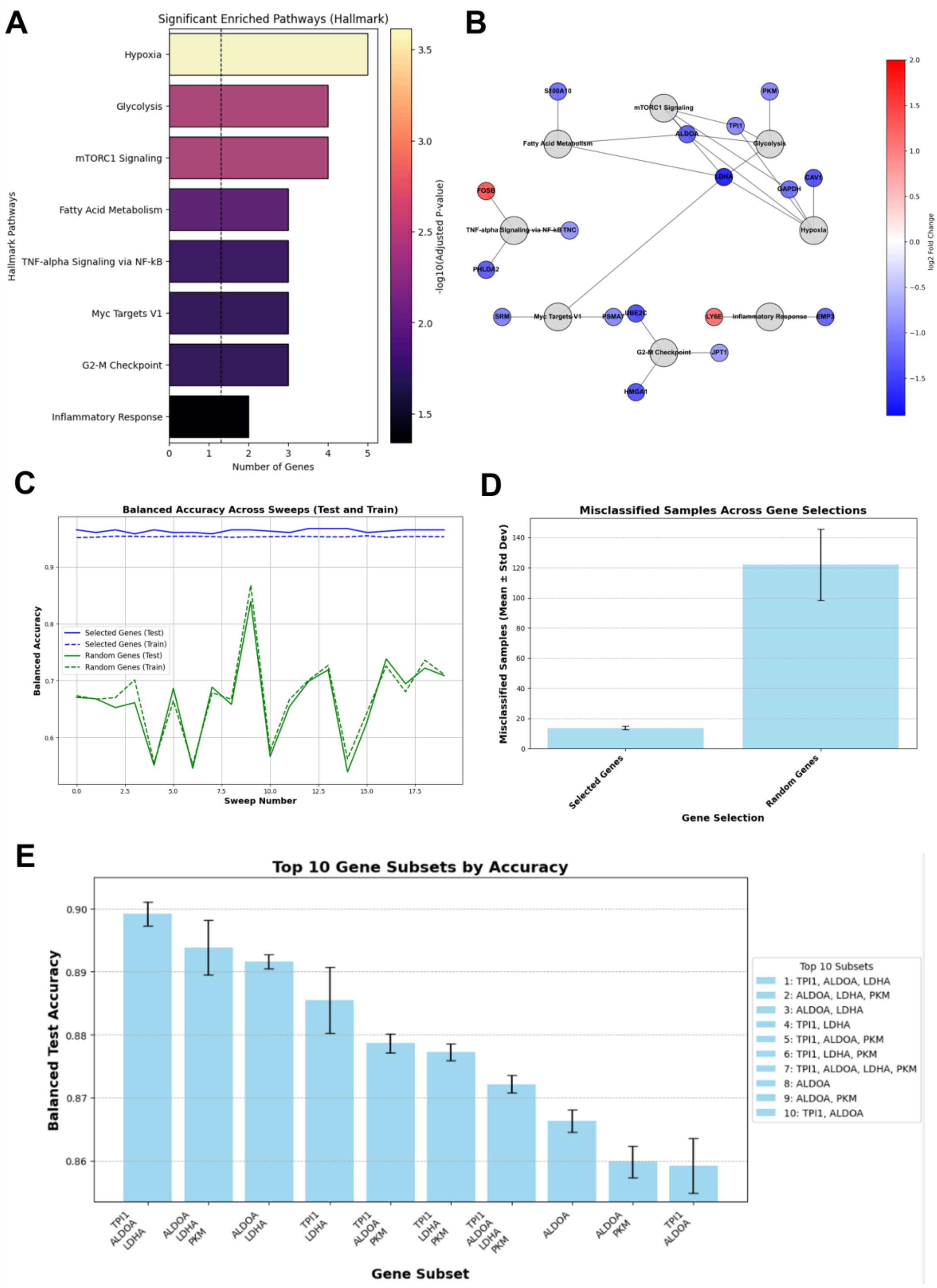

Supplementary Figure S 7: Enrichment analysis of the MI genes, especially of the MI genes associated with Glycolysis. (A) *Hallmark enrichment analysis of the top-ranked MI genes.* ***(B)*** *CNET plot of the associations between the top-ranked MI genes and the respective Hallmarks.* ***(C)*** *Balanced accuracy and predictive power of the four MI genes associated with Glycolysis, compared to four random genes.* ***(D)*** *Dependency between the four MI genes associated with Glycolysis and the number of misclassified samples compared to the number of misclassified samples for four random genes.* ***(E)*** *Balanced test accuracy for different combinations of the four MI genes associated with Glycolysis.*

The predictive power of the four MI genes is also validated by the low number of misclassified samples compared to the number of misclassified samples for four random genes (Supplementary Figure S 7D). gSELECT also offers the possibility to calculate the predictive power of individually selected genes of interest. Instead of analysing the five top-ranked MI genes (compare Supplementary Figure S 7G), we calculated the predictive power for all possible combinations of the four glycolysis-related top-ranked MI genes (Supplementary Figure S 7E). As mentioned in the Methods section and the Tutorial in the Supplementary Data, analysing more than five genes with this explorative function can require a significant amount of time and computational power, as it follows a $2^n - 1$ growth pattern where n represents the number of genes. By entering the four genes *PKM*, *ALDOA*, *TPI1*, and *LDHA*, the predictive power is automatically calculated for each of the genes alone (which results in four calculations), every possible combination of two of the genes (six possible combinations), every possible combination of three of the genes (four possible combinations), and for all four genes (one calculation). The balanced test accuracy is ranked for each of the 15 possible combinations, the ten top-ranked combinations are visualised in Supplementary Figure S 7E.

Top-ranked Mutual Information Genes: Validation by Patient Data

In addition, we performed survival analysis for the 25 top-ranked MI genes (Supplementary Figure S 8). According to the analysis, seven (*HMGA1*, *S100A10*, *TUBA1C*, *LDHA*, *TUBA1B*, *SRM*, and *PKM*) of the 25 top-ranked MI genes had a significant effect on survival. Five of them (*HMGA1*, *S100A10*, *TUBA1C*, *LDHA*, and *TUBA1B*) were also among the differentially expressed genes confirmed by every analysis method (compare Supplementary Figure S 5D).

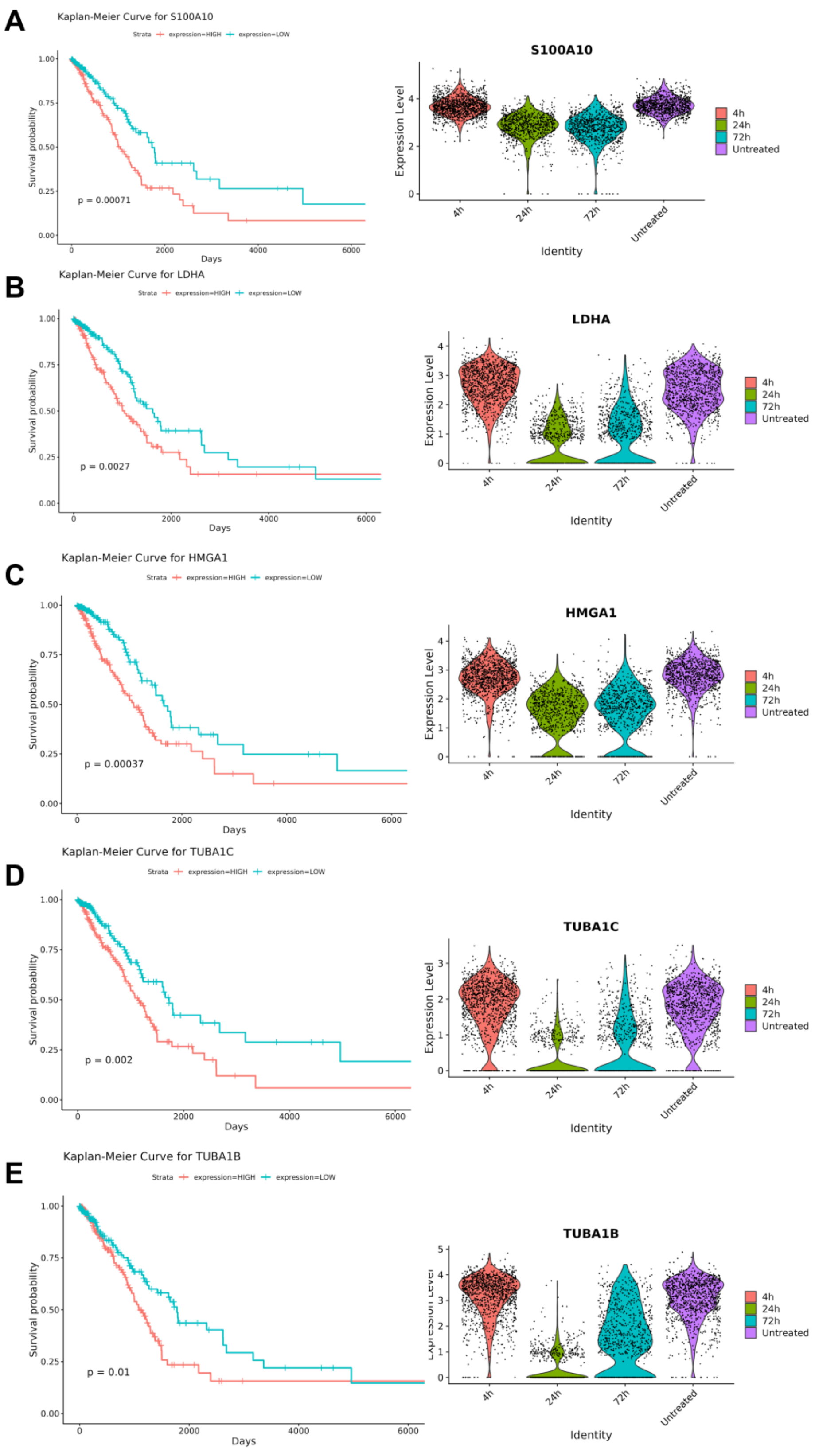

Supplementary Figure S 8: Significant results of the survival analyses of the top-ranked MI genes. (A) *Gene expression and survival analysis for S100A10.* ***(B)*** *Gene expression and survival analysis for LDHA.* ***(C)*** *Gene expression and survival analysis for HMGA1.* ***(D)*** *Gene expression and survival analysis for TUBA1C.* ***(E)*** *Gene expression and survival analysis for TUBA1B.*

The respective survival analyses and the gene expression in the different treatment groups are visualised in Supplementary Figure S 8A to E. For all five genes, low expression has been associated with a better prognosis. Therefore, their downregulation after 72 h of ARS-1620 treatment could indicate treatment success. Additionally, survival analyses and literature research, which are summarised in Supplementary Table S 3, further demonstrate the validity of the five MI genes and their potential relevance in lung cancer.

Supplementary Table S 3: *Summary of the survival data for the most relevant MI genes according to survival analysis and their role in lung cancer.*

**Gene**	**p value**	**High 5Y Survival**	**Low 5Y Survival**	**Advantage**	**Expression in 72h treated cells**	**Role in lung cancer**
S100A10	0.00123	0.28	0.40	LOW	LOW in Treated	Important for regulating the lung immune microenvironment and cancer metastasis and a potential therapeutic target for blocking cancer metastasis to the lung [47]<br>Upregulation is associated with poor prognosis in lung adenocarcinoma and in lung squamous cell carcinoma [48]
HMGA1	0.00210	0.32	0.37	LOW	LOW in Treated	High expression is associated with shorter overall survival and shorter first progression survival time in lung adenocarcinoma patients [49]<br>Correlated with poor survival in lung adenocarcinoma and involved in glycolysis in lung adenocarcinoma cells [50]
TUBA1C	0.00847	0.28	0.42	LOW	LOW in Treated	Upregulation is associated with poor prognosis in lung adenocarcinoma; additionally, expression correlated to several pathways, including cell cycle and glycolysis and is associated with tumour-infiltrating immune cells in the tumour microenvironment, possibly a new biomarker and therapeutic target [51]<br>Also highly expressed in many cancer types and associated with poor disease-specific survival and poor overall survival [52]
LDHA	0.01283	0.30	0.38	LOW	LOW in Treated	Aberrantly high expression in multiple cancers and known to facilitate glycolysis by converting pyruvate to lactate and to promote the malignant progression of tumours [53]<br>In non-small cell lung cancer, high expression of LDHA is associated with poor prognosis and radioresistance; thus, targeting LDHA might improve radiosensitivity [54]<br>In mouse models of NSCLC, inactivation of LDHA resulted in decreased tumorigenesis and disease regression [55]
TUBA1B	0.02721	0.25	0.43	LOW	LOW in Treated	In LUAD tissues, TUBA1B expression is much higher than in adjacent non-cancerous tissue, and TUBA1B expression is also correlated with clinical tumour stages. As TUBA1B was also elevated in serum, TUBA1B has been suggested as a non-invasive prognostic and diagnostic biomarker in LUAD [56]<br>Additionally, elevated TUBA1B levels have been associated with an immunosuppressive tumour microenvironment, which impacts cancer treatment outcomes and cancer progression [57]

Novel Function of gSELECT: Validating Accuracy of Gene Sets Suggested from Literature

S100A10 is not only one of the top-ranked MI genes and validated by other analysis methods and survival analysis, but it is also part of a three-gene expression signature associated with robust prediction of patient survival independent of sex, age, mutational load and smoking history [58]. Gocheva et al. (2017) analysed the extracellular matrix (ECM) composition of normal lungs, fibrotic lungs, lung tumours and metastases and observed a significantly higher abundance of S100A10 and S100A11 in the ECM of primary tumours and associated metastases [58]. Both members of the S100 protein family have already been associated with cancer: *S100A10* expression significantly correlates with more frequent vascular invasion, higher TNM stage, and a worse overall prognosis, and *S100A11* expression in LUAD significantly correlates with *KRAS* mutations and shorter disease-free survival and is associated with lymph node metastases in NSCLC [58]. *TNC*, which has also been observed in patients with lung cancer, was also upregulated in their mouse model and is known to be highly upregulated in solid tumours but absent in normal adult tissue [58].

As their three signature genes showed a significant association with survival, with high expression being associated with poorer survival, we used gSELECT to analyse whether the expression of the three genes was also affected by ARS-1620 treatment and to calculate the predictive power of the three genes. The balanced accuracy indicates a predictive power of 87% for the three signature genes (Supplementary Figure S 9A) and a relatively low number of misclassified samples (Supplementary Figure S 9B), which confirms the possible value of the three genes as prognostic markers.

As another gene of the S100 protein family, *S100A2*, was also among the top-ranked MI genes, we subsequently used the explorative function to analyse the influence of *S100A2* and the three signature genes on the predictive power. The ten top-ranked results for the possible combinations of the four genes are visualised in Supplementary Figure S 9C. In the dataset used for this analysis (untreated H358 cells compared to H358 cells after 72 h of ARS-1620 treatment), the combination of all four genes has the highest predictive power with an accuracy of more than 96%.

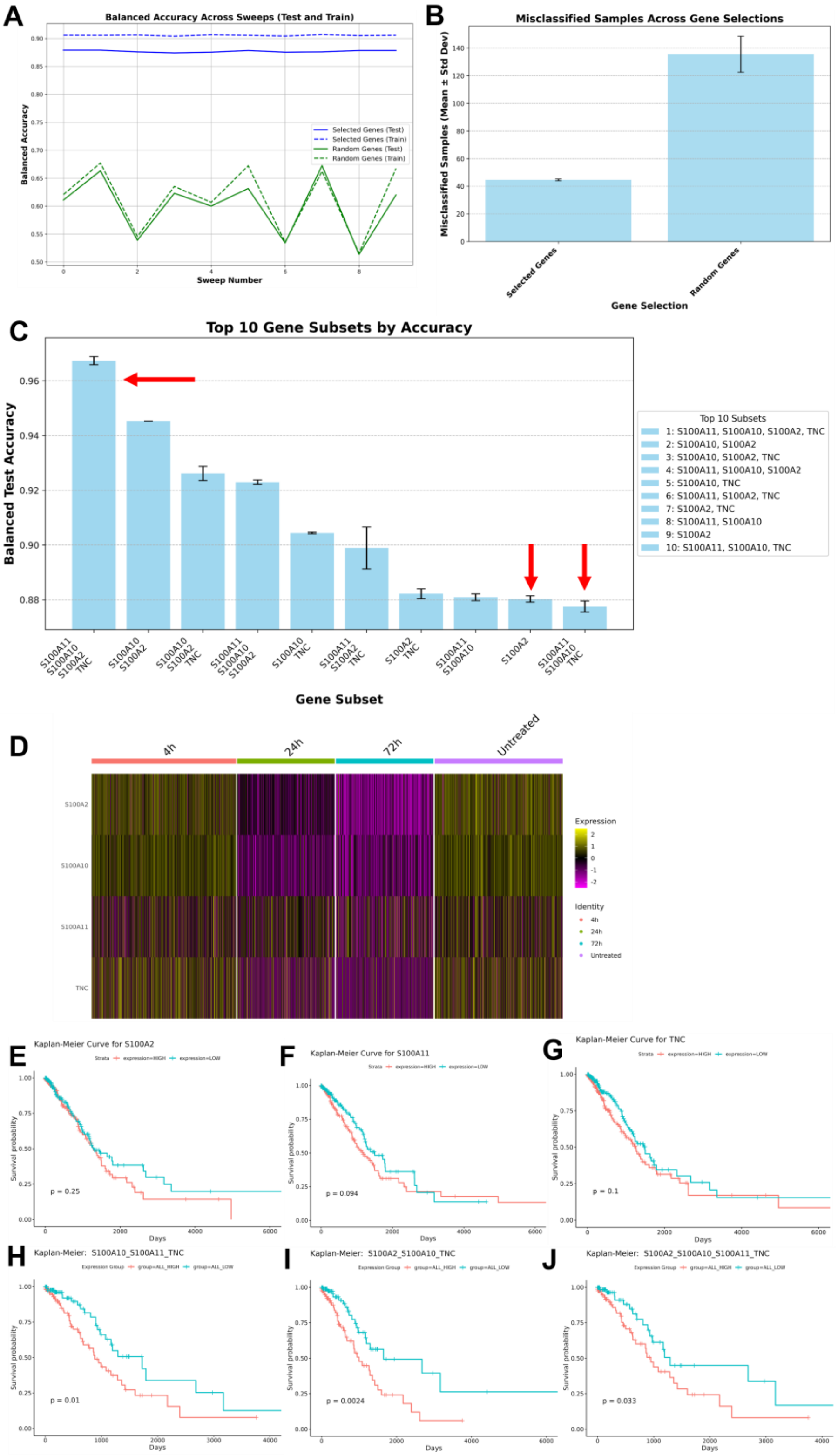

Supplementary Figure S 9: Using the explorative function to analyse the predictive power of custom genes. ***(A)*** *Balanced accuracy and predictive power of the three signature genes proposed by Gocheva et al. (2017), S100A10, S100A11, and TNC.* ***(B)*** *Dependency between the three signature genes and the number of misclassified samples compared to the number of misclassified samples for three random genes.* ***(C)*** *Balanced test accuracy for different combinations of the four genes S100A2, S100A10, S100A11, and TNC.* ***(D)*** *Heatmap visualising the gene expression of the four genes of interest after 4 h, 24 h, and 72 h of ARS-1620 treatment and in untreated H358 cells.* ***(E)*** *Survival analysis for S100A2.* ***(F)*** *Survival analysis for S100A11.* ***(G)*** *Survival analysis for TNC.* ***(E)*** *Combined survival analysis for S100A10, S100A11, and TNC.* ***(F)*** *Combined survival analysis for S100A2, S100A10, and TNC.* ***(G)*** *Combined survival analysis for S100A2, S100A10, S100A11, and TNC.*

In addition to the survival curve for *S100A10*, which is already visualised in Supplementary Figure S 8A, we also generated the survival curves for the other three genes of this analysis (Supplementary Figure S 9E to G). The survival curves for the top-ranked MI gene, *S100A2* (Supplementary Figure S 9E), the two signature genes, *S100A11* (Supplementary Figure S 9F) and *TNC* (Supplementary Figure S 9G) indicate that *TNC* and *S100A11* predominantly influence survival during the initial period, whereas *S100A2* exerts its primary effect after approximately 1000 days. We also visualised the combined survival for the combination of the three signature genes proposed by Gocheva et al. (2017) [58], *S100A10*, *S100A11*, and *TNC* (Supplementary Figure S 9H), and the combined survival curve for the combination of *TNC* and the two S100 family members that were among the top-ranked MI genes, *S100A2* and *S100A10* (Supplementary Figure S 9I), as well as the combined survival for the top-ranked combination of the four genes: all four genes are *S100A2*, *S100A10*, *S100A11* and *TNC*, see Supplementary Figure S 9J.

Finally, we compared the 10 top-ranked MI genes and the 10 top-ranked genes according to Wilcoxon, as Wilcoxon analysis is a very common analysis method for analysing single-cell data (Supplementary Figure S 10A). Both gene sets, the 10 top-ranked MI genes (yellow circle in Supplementary Figure S 10A) and the 10 top-ranked Wilcoxon genes (blue circle in Supplementary Figure S 10A), have high predictive power (top left of Supplementary Figure S 10A for the Wilcoxon genes, and top right of Supplementary Figure S 10A for the MI genes) and share six common genes: *S100A2*, *LDHA*, *HMGA1*, *PHLDA2*, *ALDOA*, and *EMP3*. Subsequently, we calculated the predictive power of the four genes that were specific to the Wilcoxon analysis (*UBE2C*, *CAV1*, *TUBA1B*, and *CXCL8*, lower left in Supplementary Figure S 10A) and for the MI genes (*S100A10*, *GAPDH*, *MT-CO1*, *PSMA7*, lower right in Supplementary Figure S 10A) where the MI genes provide more predictive power, which renders them as the most promising starting point for further analysis as they seem to encode the most information for the differences after treatment.

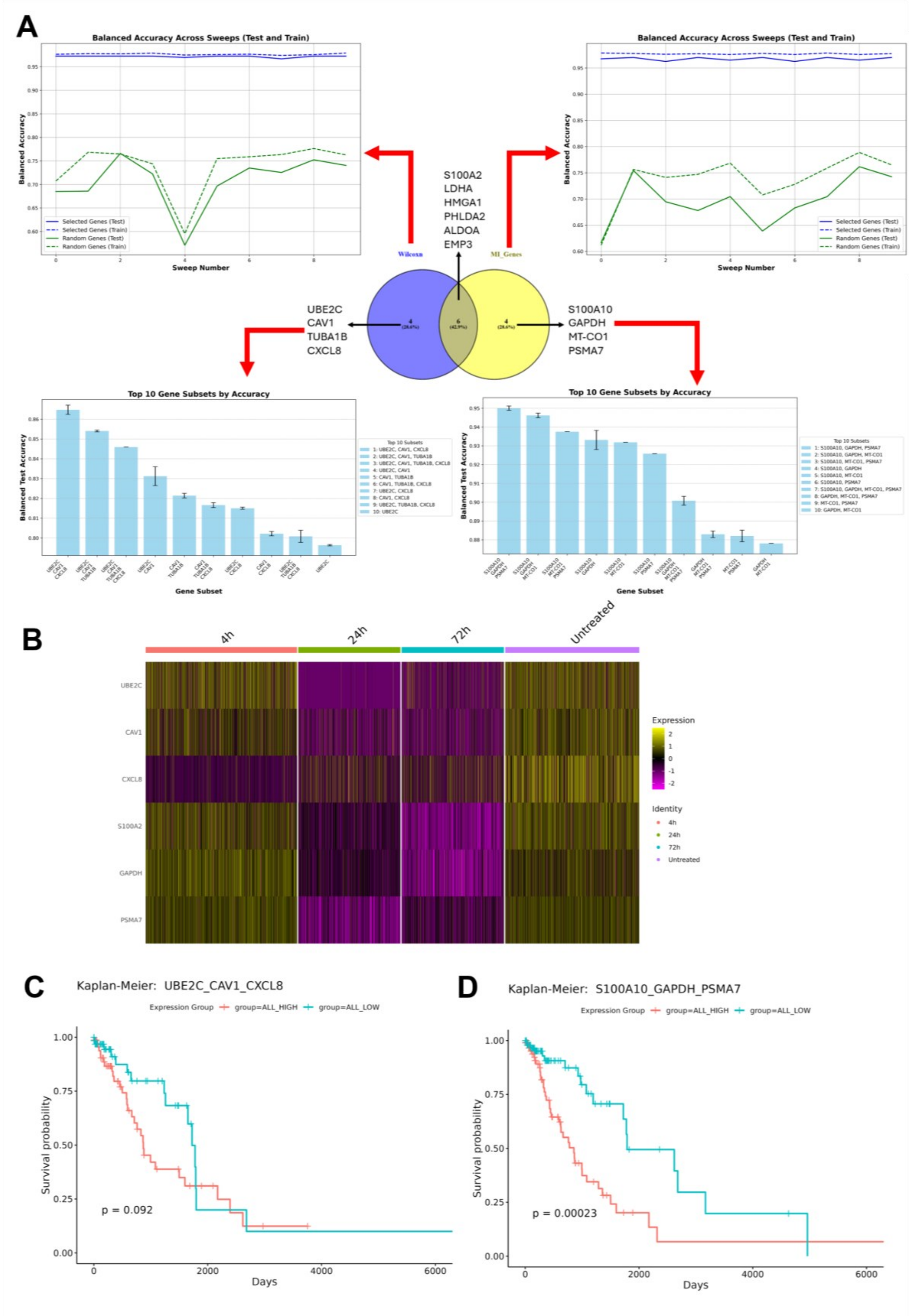

Supplementary Figure S 10: Detailed analysis of the 10 top-ranked genes according to Wilcoxon or mutual information. ***(A)*** *Venn diagram visualising the similarities and differences between the top-ranked genes according to Wilcoxon (blue) and mutual information (yellow), the predictive power of all ten genes and the predictive powers of different combinations of the genes that were specific to one of the two analyses.* ***(B)*** *Heatmap visualising the changes in gene expression of the genes of the respective combinations with the highest predictive power.* ***(C)*** *Survival analysis for the top-ranked combination of genes specific to Wilcoxon.* ***(D)*** *Survival analysis for the top-ranked combination of genes specific to mutual information.*

Similarly, we can use the gSELECT pipeline to compare any gene set suggestion of any method to start with the most promising ones to explain the observations or differences of interest. Except for *CXCL8*, all of the genes were among the top-ranked MI genes for the comparison of untreated H358 cells and H358 cells after 72 h of ARS-1620 treatment. While the highest achieved balanced accuracy for the "only Wilcoxon genes" was slightly more than 86% (for *UBE2C*, *CAV1* und *CXCL8*, lower left of Supplementary Figure S 10A), the best combination of the "only MI genes" (*S100A10*, *GAPDH*, and *PSMA7*, lower right of Supplementary Figure S 10A) achieved a balanced accuracy of about 95%, Supplementary Figure S 10B visualises the gene expression of the respective genes as a heatmap, showing that all genes except for *CXCL8* are downregulated after ARS-1620 treatment for 24 h or 72 h, while *CXCL8* is downregulated after 4 h of ARS-1620 treatment compared to untreated cells, but appears to be upregulated after 24 h or 72 h of treatment. The respective combined survival plots for the genes only identified using Wilcoxon (Supplementary Figure S 10C) or only according to mutual information (Supplementary Figure S 10D), and the respective data is summarised in Supplementary Table S 4.

Supplementary Table S 4: *Summary of the survival data for the most relevant MI genes according to survival analysis and their role in lung cancer.*

**Gene**	**Analysis Method**	**p value**	**High 5Y Survival**	**Low 5Y Survival**	**Advantage for Survival**
UBE2C	Wilcoxon	0.22455	0.35	0.31	HIGH
CAV1	Wilcoxon	0.05142	0.33	0.33	HIGH
CXCL8	Wilcoxon	0.41551	0.34	0.33	HIGH
S100A10	MI	0.00123	0.28	0.40	LOW
GAPDH	MI	0.31646	0.38	0.29	HIGH
PSMA7	MI	0.27120	0.28	0.42	LOW

Additional Validation of the gSELECT MI Genes and Benchmarking

To validate the importance of the MI genes obtained via gSELECT analysis, we analysed the single cell data published by Xue et al. (2020) [39] (GSE137912), using several standard methods.

Besides using three different test methods available via Seurat's FindMarkers() function ('wilcox' (for Wilcoxon as standard Seurat method), 'DESeq2' (for DESeq2 (1.44.0) [21] analysis), and 'MAST' (for MAST (1.30.0) [22] analysis)), which were used to validate the MI genes in the text above (Methods described in the Detailed Methods Section, Results described in the Lung Case Study Scenarios), we also performed additional analyses with other R-based and python-based standard methods, which are described below.

However, our aim is not to replace existing standard methods, but to complement them by offering additional functionalities. These functionalities include the ability to pre-analyse datasets to identify whether further analysis may be warranted, and to assess whether specific genes warrant further investigations, including experimental validation. Therefore, we only benchmark the accuracy of the MI genes by comparing the analysis results of various standard methods with the predicted MI genes, using Seurat's FindMarkers() with different test methods ('wilcox', 'DESeq2', and 'MAST'), scanpy's rank_genes_groups with two different methods (Wilcoxon and t-test with overestimated variance), AUROC analysis (in Python and via Seurat's FindMarkers() with 'roc' as test used), and scClassify, to analyse the comparison between untreated H358 cells and H358 cells after 72 h of ARS-1620 treatment.

Seurat FindMarkers

To validate the MI genes for the third scenario, Seurat's FindMarkers() function was used with three different methods: Wilcoxon rank sum test (the default option in Seurat) [12, 16-18, 40], DESeq2 analysis [21], and MAST [22]. The resulting 25 top-ranked genes (with adjusted p-value < 0.05, and

sorted by absolute log2fold change) and their overlap with the top 25 MI genes are visualised in Supplementary Figure S 11 and summarised in Supplementary Table S 5 (MI genes written in bold).

When using ‘wilcox’ and ‘MAST’ as test method, the 25 top-ranked MI genes and the 25 top-ranked genes identified using Seurat’s FindMarkers() function, have 16 common genes. For ‘DESeq2’, it results in 15 common genes.

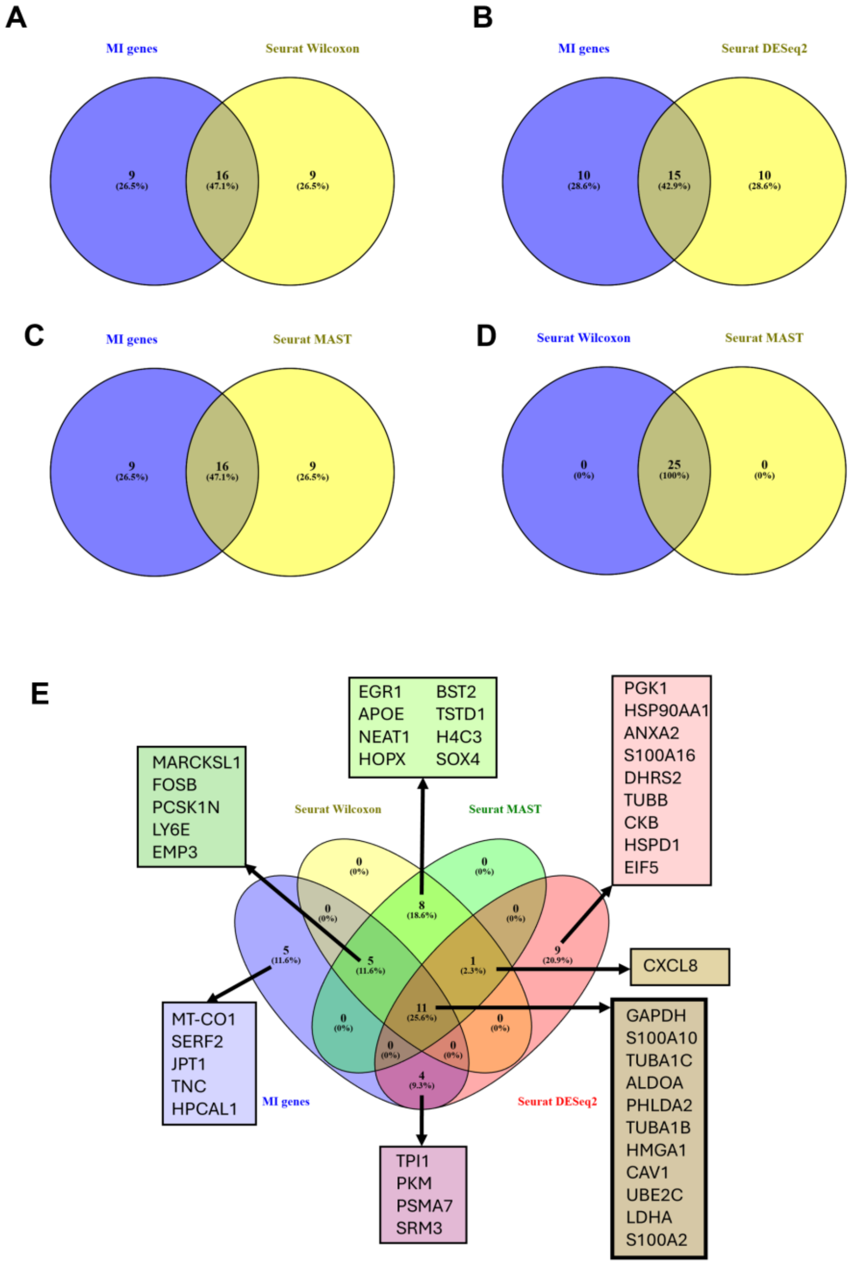

Supplementary Figure S 11: Comparison of the 25 top-ranked MI genes and the 25 top-ranked genes identified using the Seurat function FindMarkers() with different methods based on the comparison between untreated H358 cells and H358 cells after 72 h of ARS-1620 treatment. (A) *MI genes identified using gSELECT (blue ellipse) compared with genes identified with Seurat’s FindMarkers() function using ‘wilcox’ as method (yellow ellipse).* ***(B)*** *MI genes identified gSELECT (blue ellipse) compared with genes identified with Seurat’s FindMarkers() function using ‘DESeq2’ as method (yellow ellipse).* ***(C)*** *MI genes identified using gSELECT (blue ellipse) compared with genes identified with Seurat’s FindMarkers() function using ‘MAST’*

as method (yellow ellipse). ***(D)*** *Using FindMarkers() with 'wilcox' (blue ellipse) and 'MAST' (yellow ellipse) identified the same genes.* ***(E)*** *11 MI genes were also identified by all three tests of Seurat's FindMarkers() function.*

Supplementary Table S 5: *The top-ranked genes according to Seurat's FindMarkers() using "wilcoxon", "DESeq2", and "MAST" as method. Genes also among the MI genes are written in bold.*

Wilcoxon (via Seurat)	DESeq2 (via Seurat)	MAST (via Seurat)
**S100A2**	**S100A2**	**S100A2**
EGR1	**LDHA**	EGR1
**LDHA**	**CAV1**	**LDHA**
**UBE2C**	**PHLDA2**	**UBE2C**
**CAV1**	**ALDOA**	**CAV1**
**HMGA1**	S100A10	**HMGA1**
**MARCKSL1**	CXCL8	**MARCKSL1**
APOE	**TUBA1B**	APOE
**FOSB**	**HMGA1**	**FOSB**
**TUBA1B**	**GAPDH**	**TUBA1B**
**PHLDA2**	PGK1	**PHLDA2**
**ALDOA**	**UBE2C**	**ALDOA**
**EMP3**	HSP90AA1	**EMP3**
NEAT1	**TUBA1C**	NEAT1
CXCL8	**PSMA7**	CXCL8
HOPX	ANXA2	HOPX
**PCSK1N**	**TPI1**	**PCSK1N**
**TUBA1C**	**S100A16**	**TUBA1C**
BST2	**PKM**	BST2
**LY6E**	DHRS2	**LY6E**
**S100A10**	TUBB	**S100A10**
TSTD1	CKB	TSTD1
H4C3	HSPD1	H4C3
**GAPDH**	**SRM**	**GAPDH**
SOX4	EIF5	SOX4

Scanpy rank_genes_groups

To analyse the data using scanpy [4], we imported the same h5ad file that was imported in R for Seurat analysis in python and subset the resulting AnnData object for "untreated" and "72h" using pandas [6]. After saving the raw counts as a layer, and filtering for genes expressed in ≥ 10% of cells of at least one of the compared groups, to match the filtering criteria applied in the Seurat analyses described above (using FindMarkers() with different methods for test.use ('wilcox', 'DESeq2', 'MAST')), we used scanpy (with flavor "cell_ranger") to find highly variable genes. Subsequently, we performed

normalization and log-transformation and set the categories for the subsequent differential expression analysis, which was performed using scanpy's rank_genes_groups grouped by treatment, with Benjamini-Hochberg as correction method and two different methods: Wilcoxon and t-test with overestimated variance, which overestimates the variance of each group. The resulting genes were ranked according to their absolute scores (|score|), and the 25 top-ranked genes are summarised in Supplementary Table S 6. 20 of the top 25 genes obtained using 'wilcoxon' as method, and 22 of the top 25 genes obtained using 't-test_overestim_var' as method, are also among the 25 top-ranked MI genes, and 19 MI genes were identified by both methods, further validating the analysis method (visualised in Supplementary Figure S 12 and written in bold in Supplementary Table S 6).

Supplementary Table S 6: *The top-ranked genes according to scanpy rank_genes_groups using "wilcoxon" and "t-test_overestim_var" as method. Genes also among the MI genes are written in bold.*

**Wilcoxon**	**t-test with overestimated variance (t-test_overestim_var)**
**S100A2**	**S100A2**
**S100A10**	**S100A10**
**MT-CO1**	**LDHA**
**ALDOA**	**GAPDH**
**GAPDH**	**ALDOA**
**PHLDA2**	**PHLDA2**
**LDHA**	**HMGA1**
**PSMA7**	**LY6E**
**HMGA1**	EGR1
**LY6E**	**EMP3**
**PCSK1N**	**MARCKSL1**
**TPI1**	**PCSK1N**
**MARCKSL1**	**CAV1**
**FOSB**	**PSMA7**
**CAV1**	**TPI1**
TSTD1	**UBE2C**
**PKM**	**FOSB**
EGR1	**TUBA1B**
**EMP3**	TSTD1
**SRM**	**TUBA1C**
**JPT1**	**SRM**
BST2	**MT-CO1**
EIF5	**PKM**
**TUBA1B**	**HPCAL1**
NME1	BST2

Additionally, 17 of the 25 top-ranked genes identified using ‘wilcoxon’ (S100A2, EGR1, LDHA, CAV1, HMGA1, MARCKSL1, FOSB, TUBA1B, PHLDA2, ALDOA, EMP3, PCSK1N, BST2, LY6E, S100A10, TSTD1, and GAPDH, Supplementary Figure S 12A) were also identified with Seurat’s FindMarkers() function using ‘wilcox’ as method, which might be due to the slight differences in the respective analyses.

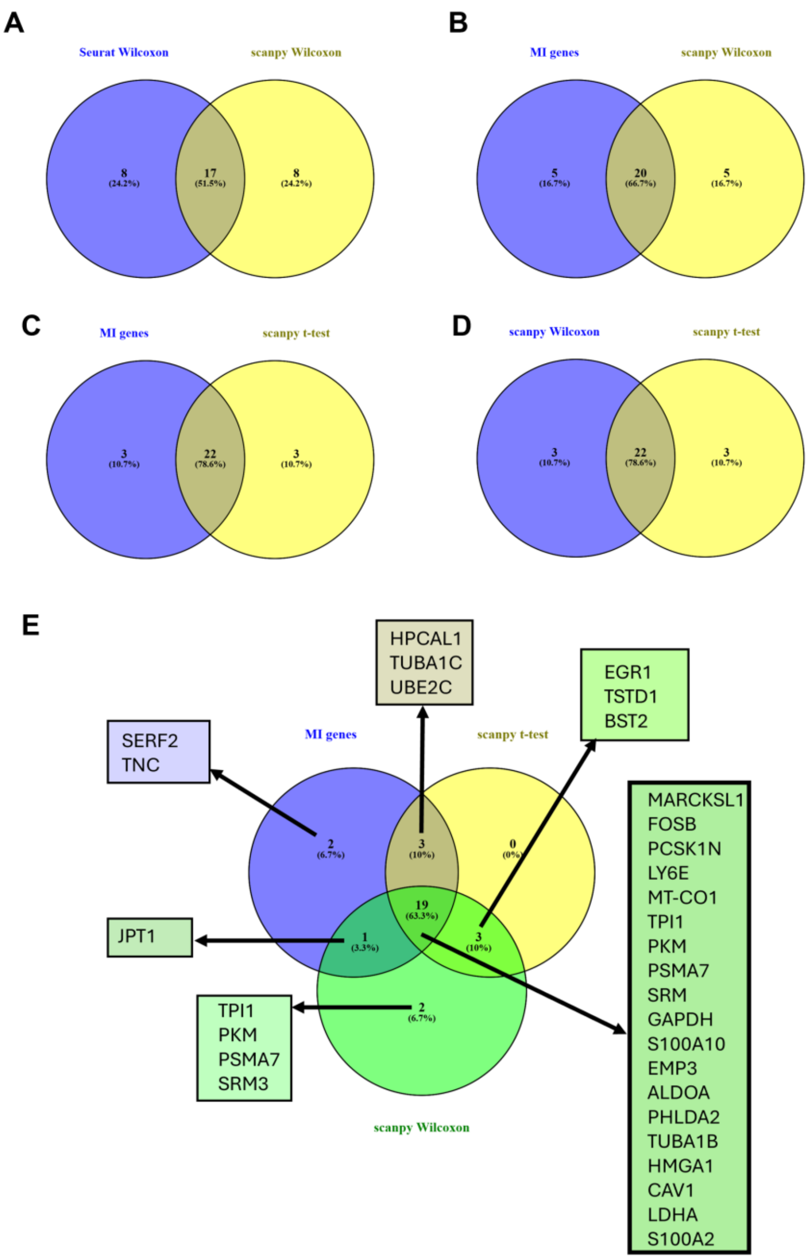

Supplementary Figure S 12: Comparison of the 25 top-ranked MI genes and the 25 top-ranked genes identified using scanpy’s rank_genes_groups with different methods based on the comparison between untreated H358 cells and H358 cells after 72 h of ARS-1620 treatment. (A) *Using the Wilcoxon rank sum test as test method resulted in 17 common genes between the Seurat’s FindMarkers (blue ellipse) and scanpy’s rank_genes_groups (yellow ellipse).* ***(B)*** *MI genes identified using*

gSELECT (blue ellipse) compared with genes identified with scanpy's rank_genes_groups using 'wilcoxon' (yellow ellipse). ***(C)*** *MI genes identified using gSELECT (blue ellipse) compared with genes identified with scanpy's rank_genes_groups using 't-test_overestim_var' (yellow ellipse).* ***(D)*** *scanpy's rank_genes_groups using 'wilcoxon' and 't-test_overestim_var' identified 22 common genes.* ***(E)*** *19 MI genes were identified by both scanpy rank_genes_groups methods.*

AUROC

For the Area Under the Receiver Operating Characteristic (AUROC) curve, the data was prepared analogous to the preparation of the data for scanpy rank genes (loading the same h5ad file, creating a subset of two groups, "Untreated" and "72h"), before a binary vector was created (0 = "Untreated", 1 = "72h"). The gene expression matrix of the subset was used to extract the expression values of each gene. Genes with no variation in gene expression were skipped and their AUROC score set to 0.5, for the other genes, the AUROC scores were calculated using roc_auc_score() from scikit-learn [42] and transformed to a 0-1 effect-size metric ('power' = $2 \cdot |AUROC - 0.5|$) for downstream ranking. The 25 top-ranked genes are summarised in Supplementary Table S 7. Additionally, Seurat's FindMarkers() function, which was described above, using 'roc' as used test, resulted in 19 genes (bold in Supplementary Table S 7 (right)), which were among the top-ranked results in Seurat FindMarkers() with 'roc' and among the top-ranked MI genes (Supplementary Figure S 13).

Supplementary Table S 7: *The 25 top-ranked genes according to AUROC curve, performed in python (left) and R via Seurat's FindMarkers() function using 'roc' (right).*

**AUROC (python)**	**AUROC (Seurat FindMarkers())**
**S100A2**	**S100A2**
**S100A10**	**S100A10**
**PHLDA2**	**ALDOA**
**ALDOA**	**GAPDH**
**HMGA1**	**PHLDA2**
**GAPDH**	**PSMA7**
**TPI1**	**LDHA**
**LDHA**	**HMGA1**
**PSMA7**	**LY6E**
S100A16	TPI1
YBX1	**PCSK1N**
**SRM**	**MARCKSL1**

UBE2E3	**SERF2**
TXN	**CAV1**
EEF1D	**FOSB**
**CAV1**	**PKM**
PFN1	TSTD1
**PKM**	EGR1
AURKAIP1	**EMP3**
PRELID1	**SRM**
EIF5	**JPT1**
CHCHD2	EIF5
**JPT1**	BST2
GADD45GIP1	EEF1D
NME1	**TUBA1B**

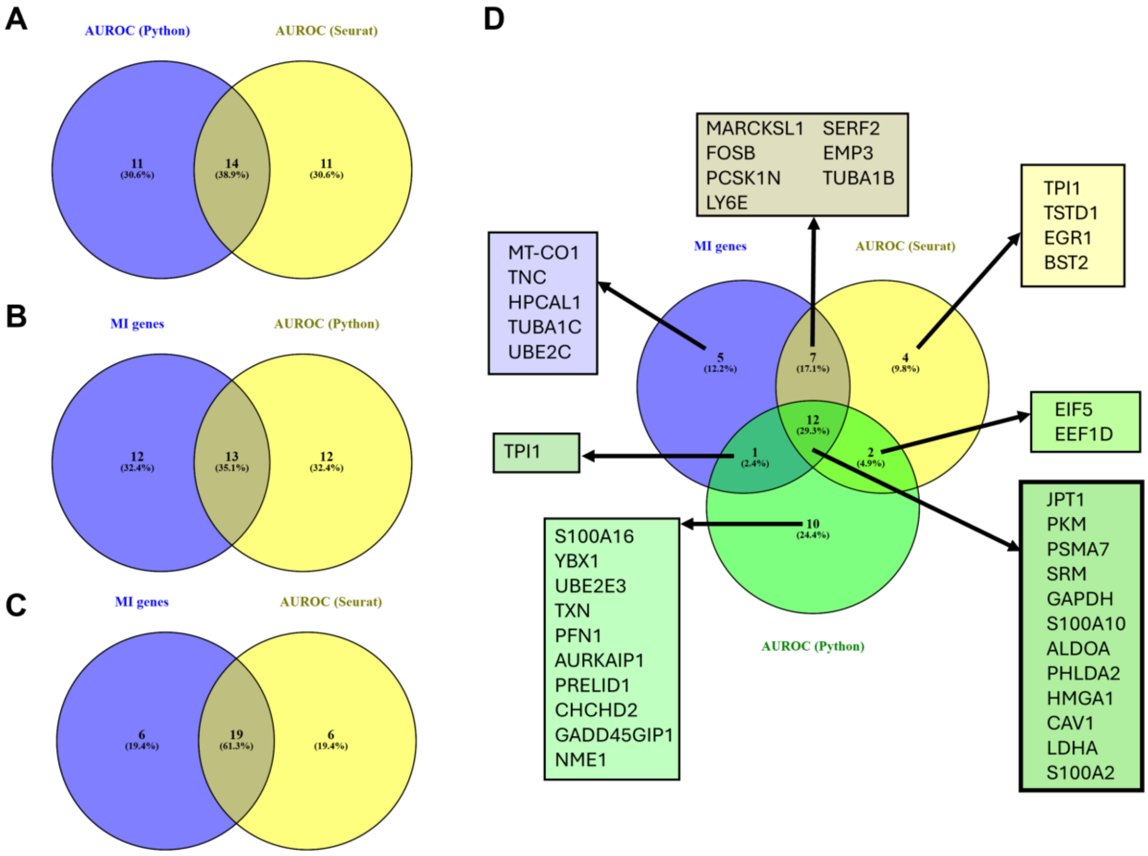

Supplementary Figure S 13: Comparison of the 25 top-ranked MI genes and the 25 top-ranked genes identified using AUROC on the comparison between untreated H358 cells and H358 cells after 72 h of ARS-1620 treatment. (A) *14 genes were identified by both AUROC in Python (blue ellipse) and AUROC via Seurat's FindMarkers() function using 'roc' (yellow ellipse).* ***(B)*** *MI genes identified using gSELECT (blue ellipse) compared with genes identified with AUROC in Python (yellow ellipse).* ***(C)*** *MI genes identified using gSELECT (blue ellipse) compared with genes identified with AUROC via Seurat's FindMarkers() function using 'roc' (yellow ellipse).* ***(D)*** *12 MI genes were also identified by both AUROC analyses.*

scClassify

To compare the top-ranked MI genes with the results of tree-based selectors in scClassify [41] (version 1.20.0), we performed the first data preparation steps analogous to the Seurat analysis workflow (loading the data, subsetting the data, and normalizing the data), before we obtained the log-normalized matrix from the data slot, defined the two levels ("Untreated" and "72h"), and performed a 80/20 split using caret [59] (version 7.0-1). Subsequently, scClassify was run using "HC" (using hclust) as tree, the default algorithm "WKNN" and the default gene selection method "limma", and "pearson" (the default) and additionally "spearman" as similarity measures. All 211 of untreated H358 cells were predicted correctly as untreated cells, out of the 161 cells in the 72h group, 130 were predicted correctly as "72h", 21 were unassigned, and 10 were incorrectly predicted as "untreated". The top-ranked 25 genes of the resulting hvg-list are summarised in Supplementary Table S 8 (MI genes in bold) and visualised in Supplementary Figure S 14B.

Supplementary Table S 8: *The 25 top-ranked genes according to the scClassify analysis, MI genes are written in bold.*

scClassify	MI genes
**S100A2**	MARCKSL1
**ALDOA**	FOSB
**LDHA**	PCSK1N
**PHLDA2**	LY6E
**HMGA1**	MT-CO1
**EMP3**	SERF2
**CAV1**	JPT1
**SRM**	TNC
**TUBA1B**	TPI1
**TUBA1C**	HPCAL1
**UBE2C**	PKM
**PKM**	PSMA7
S100A16	SRM
UBE2E3	GAPDH
**HPCAL1**	S100A10
NME1	TUBA1C
PGK1	EMP3
TUBB	ALDOA
EIF5	PHLDA2
PRELID1	TUBA1B

PHF19	HMGA1
LYAR	CAV1
RANBP1	UBE2C
HMGB2	LDHA
MAL2	S100A2

The volcano plot in Supplementary Figure S 14A shows all differentially regulated genes, based on SeuratFindMarkers() with 'wilcox' and thresholds set to 0. All significant (adjusted p-value < 0.05) genes that are up- or downregulated with an absolute log2 fold change > 1 are visualised in red or blue, respectively. Out of 13660 genes, 1140 were significant DEGs, 552 of which were upregulated and 618 were downregulated.

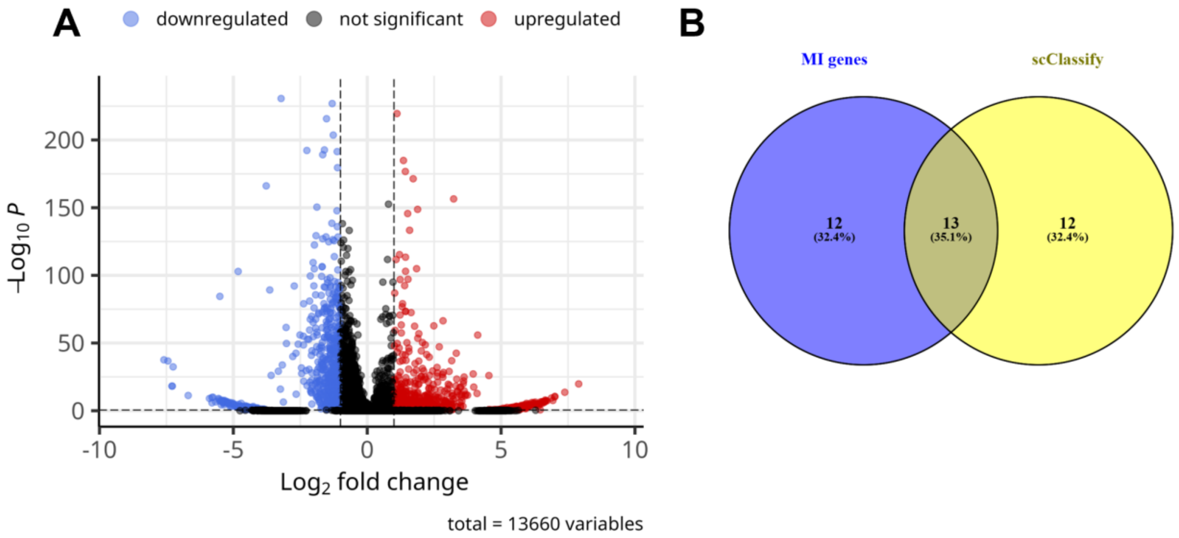

Supplementary Figure S 14: Differentially expressed genes and comparison between MI genes and genes identified via scClassify. (A) *Volano plot visualisation of the differentially expressed genes according to Seurat's FindMarkers() using Wilcoxon rank sum test.* ***(B)*** *Venn diagram comparing the top-ranked MI genes and the top-ranked genes identified using scClassify.*

In silico Validation of the 25 top-ranked MI Genes

To conclude, we also compared the 25 top-ranked MI genes with the top 25 genes identified by all the methods mentioned above, which is visualised as a pairwise intersection matrix (displayed as minimal triangular matrix, coloured by Jaccard index to better visualise the similarity between different groups, generated online using molbiotools' Multiple List Comparator [60], available at https://molbiotools.com/listcompare.php, accessed on 15th of July 2025) in Supplementary Figure S 15 and summarised in Supplementary Table S 9. Except for TNC, which is among the differentially expressed genes validated using DESeq2 (Supplementary Figure S 5D), but not among the top-ranked 25 of these genes, all of the 25 top-ranked MI genes could be validated by at least another method for finding differentially expressed genes/markers/genes relevant to the differences between the two groups (untreated and 72 h ARS-treated H358 cells).

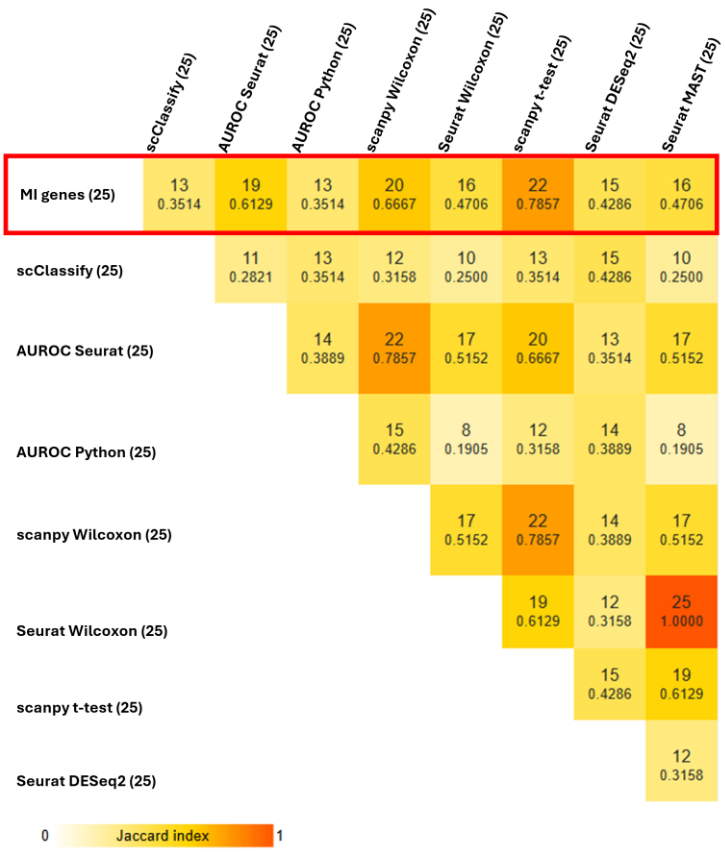

Supplementary Figure S 15: Pairwise intersection matrix of the common genes identified with the various methods described above. *Displayed as minimal triangular matrix, coloured by Jaccard index to better visualise the similarity between different groups, generated online using molbiotools' Multiple List Comparator [60] (available at https://molbiotools.com/listcompare.php, accessed on 15th of July 2025), The bigger number indicates the respective intersection sizes, the smaller number below displays the Jaccard index values.*

Supplementary Table S 9: *MI genes also identified by other standard analysis methods. The number of identified MI genes is next to the respective method's name, the common genes are shown below.*

**MI Genes**	**scClassify (13)**	**AUROC Seurat (19)**	**AUROC Python (13)**	**scanpy Wilcoxon (20)**	**Seurat Wilcoxon (16)**	**scanpy t-test (22)**	**Seurat DESeq2 (15)**	**Seurat MAST (16)**
ALDOA	**ALDOA**	**ALDOA**	**ALDOA**	**ALDOA**	**ALDOA**	**ALDOA**	**ALDOA**	**ALDOA**
CAV1	**CAV1**	**CAV1**	**CAV1**	**CAV1**	**CAV1**	**CAV1**	**CAV1**	**CAV1**
HMGA1	**HMGA1**	**HMGA1**	**HMGA1**	**HMGA1**	**HMGA1**	**HMGA1**	**HMGA1**	**HMGA1**
LDHA	**LDHA**	**LDHA**	**LDHA**	**LDHA**	**LDHA**	**LDHA**	**LDHA**	**LDHA**
PHLDA2	**PHLDA2**	**PHLDA2**	**PHLDA2**	**PHLDA2**	**PHLDA2**	**PHLDA2**	**PHLDA2**	**PHLDA2**
S100A2	**S100A2**	**S100A2**	**S100A2**	**S100A2**	**S100A2**	**S100A2**	**S100A2**	**S100A2**
GAPDH		GAPDH	GAPDH	GAPDH	GAPDH	GAPDH	GAPDH	GAPDH
S100A10		S100A10	S100A10	S100A10	S100A10	S100A10	S100A10	S100A10
TUBA1B	TUBA1B	TUBA1B		TUBA1B	TUBA1B	TUBA1B	TUBA1B	TUBA1B
EMP3	EMP3	EMP3		EMP3	EMP3	EMP3		EMP3
PKM	PKM	PKM	PKM	PKM		PKM	PKM	
SRM	SRM	SRM	SRM	SRM		SRM	SRM	
FOSB		FOSB		FOSB	FOSB	FOSB		FOSB
LY6E		LY6E		LY6E	LY6E	LY6E		LY6E
MARCKSL1		MARCKSL1		MARCKSL1	MARCKSL1	MARCKSL1		MARCKSL1
PCSK1N		PCSK1N		PCSK1N	PCSK1N	PCSK1N		PCSK1N
PSMA7		PSMA7	PSMA7	PSMA7		PSMA7	PSMA7	
TUBA1C	TUBA1C				TUBA1C	TUBA1C	TUBA1C	TUBA1C
UBE2C	UBE2C				UBE2C	UBE2C	UBE2C	UBE2C
TPI1			TPI1	TPI1		TPI1	TPI1	
HPCAL1	HPCAL1					HPCAL1		
MT-CO1				MT-CO1		MT-CO1		
JPT1		JPT1	JPT1	JPT1				
SERF2		SERF2						
TNC								

Analysing the data using different standard analysis methods demonstrates the potential importance of the MI genes, as all of the 25 top-ranked MI genes were also identified using other methods. Except for TNC, all of the top 25 MI genes were even among the 25 top-ranked genes identified by other methods. Six genes (ALDOA, CAV1, HMGA1, LDHA, PHLDA2, and S100A2) were even among the 25 top-ranked genes identified by every standard method used for validation. Additionally, to benchmark gene sets for group classification, we computed the area under the receiver operating characteristic curve (AUROC) using logistic regression in R. For each gene set (the respective 25 top-ranked genes identified by the methods described above), an expression matrix was subset to the respective genes, and a binary classification model was trained using caret [59] (version 7.0-1). Probabilities were obtained using

predict() and AUROC values were computed via the pROC package [61] (version 1.18.5). The respective plots were generated using ggplot2 [19] (version 3.5.2), and are visualised in Supplementary Figure S 16.

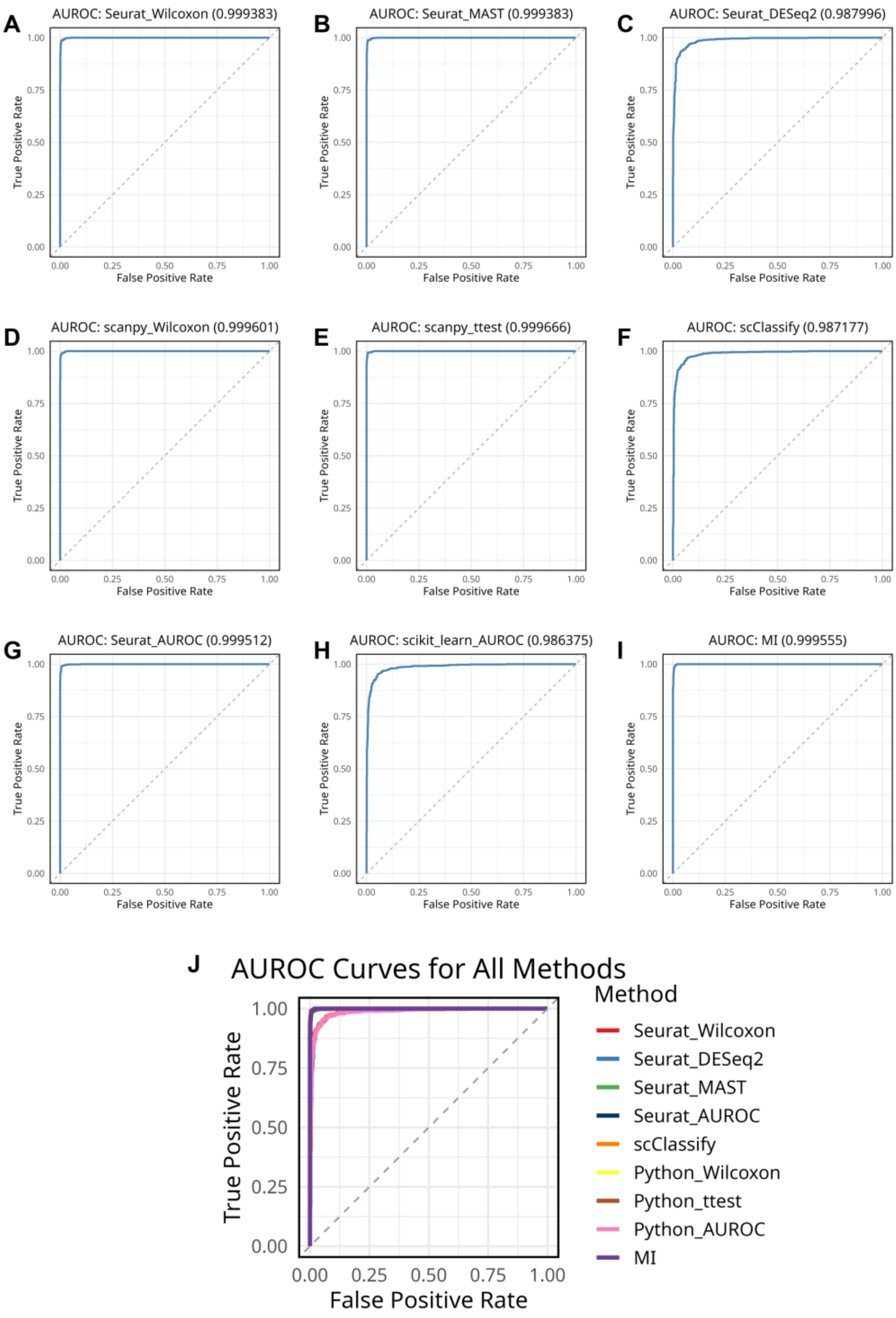

Supplementary Figure S 16: AUROC for the 25 top-ranked genes of each method used to validate the MI genes. *(A) AUROC for the genes identified using Seurat's FindMarkers() using 'wilcox'.* ***(B)*** *AUROC for the genes identified using Seurat's FindMarkers() using 'MAST'.* ***(C)*** *AUROC for the genes identified using Seurat's FindMarkers() using 'DESeq2'.* ***(D)*** *AUROC for the genes identified using scanpy's rank_genes_groups using 'wilcoxon'.* ***(E)*** *AUROC for the genes identified using scanpy's rank_genes_groups using 't-test_overestim_var'.* ***(F)*** *AUROC for the genes identified using scClassify's tree-based classification.* ***(G)*** *AUROC for the genes identified using Seurat's FindMarkers() using 'roc'.* ***(H)*** *AUROC for the genes identified using scikit-learn' roc_auc_score().* ***(H)*** *AUROC for the MI genes.* ***(H)*** *Overlay of AUROC curves for all methods.*

These results demonstrate that gSELECT is able to find potentially relevant genes as well as other standard methods, proving its usefulness as a pre-analysis tool. Of course, as with all in silico identified genes, further experimental validation of the genes is required. However, this is precisely the purpose of gSELECT: to support decision-making and helping to prioritise genes that may merit further laboratory investigations. Used as a pre-analysis tool, gSELECT can highlight potentially informative genes (the MI genes). Additionally, the explorative function of gSELECT, which was introduced in the main text and above in the chapter describing the third scenario of two clearly different groups, allows evaluating user-identified genes of interest (e.g., genes of interest from literature research).

Additional Literature Validation of Selected MI Genes

Since the main intention of this work is to show how different well-established methods can be combined to validate computational results, we omit to explain all the different methods but focus on the explanation of the combination of the well-established methods.

For quantifying the predictive power of a set of genes regarding predicting the corresponding phenotypic feature it holds the following. One or more models can be fit as an approximation of the function mapping the values of the gene expression to the phenotype according to a split of the data set into training and test set. If this is possible with a sufficient accuracy, the gene selection carries sufficient information to describe the separation of the corresponding cells into phenotypes. For the comparison, the same model with its configuration is fit to all available genes where genes with a constant expression pattern are filtered out. Since in the total feature set (all genes with a non-constant expression pattern), all available information given the data are available, the rationale is to get the best possible predictive power and thus the upper bound to detect information loss during the gene selection process. Furthermore, a model is trained several times on the same number of genes that have been randomly selected among the total number of genes that have a non-constant gene expression to get the lower bound that a random selection could already achieve in terms of predictive power for separating the phenotypes. We remark that if a model fails in its accuracy to separate the phenotypes, it does not necessarily mean the selection of genes is not good in the sense that it does not have the power to

separate. Maybe the fitting has gone wrong, e.g., a not beneficial convergence or hyperparameters, or the model's architecture is not suited to fit the corresponding relations. For this reason, in case of failure, one helpful option is to train several models to exclude such an issue as well as possible. Another option is to use UMAP or t-SNE to check for a clear clustering. If there is one, there must be information that allow this separation.

This comparison makes it more likely that the selected genes provide meaningful biological insights rather than being artifacts of the computational approach.

In the following, we will first evaluate the efficacy of our newly developed tool in identifying key genetic markers. Second, we examine the specific genes identified by this tool and address their respective roles in lung cancer. The relevance of these genes not only confirms the efficacy of our novel tool gSELECT but also highlights the potential importance of the respective genes in lung cancer.

We remark that tools like Perplexity.ai or any other generative AI combined with internet search might further extend our options to analyse the causal relations of small sets of genes that have a high predictive power regarding a phenotypic difference. For example, we can insert the set of genes identified with gSELECT and ask Perplexity.ai why these genes are important to explain the observed phenotypic differences or why influencing them can affect the observed phenotypic differences. For example, we could ask if and why certain MI genes (e.g., the top-ranked MI genes, or MI genes associated with a certain pathway, …) are important for survival of lung cancer patients. These tools might identify relevant publications or documents and generate a helpful answer that allows us to come up with corresponding hypotheses for the causal relations with corresponding tests to validate them. Since gSELECT requires little user input, and AI-based literature research is faster than human literature research, this combination offers a timesaving first analysis step. However, the results of the AI-based literature research still need to be validated by a human researcher. Nevertheless, validating if the cited sources really contain the respective information is faster and more convenient than performing manual literature research.

At the same time, gSELECT can validate genes of interest that have been identified as promising candidates in literature research (manual or AI-supported literature research or previous knowledge).

Here, a (publicly available) h5ad file of an analysis similar to the intended research question (e.g., a dataset containing different lung cancer cells after a certain treatment) can be loaded in gSELECT to analyse the predictive power of the custom genes of interest. By combining gSELECT and an automated AI-based literature research, future refinements of gSELECT could also link the power of analysing biological data with the advantages of AI in scientific research, particularly in literature research. Our gSELECT might in particular be helpful in this scenario as with smaller gene sets, the AI can be more precise and limits its options for answers to specific but important cases as their context length, in which all the retrieved documents have to fit, could be limited. Having too many irrelevant candidates might result in not considering important information.

Performance of gSELECT in Different Scenarios

The first possible scenario, two almost identical groups, which is discussed in detail in the main text, shows that gSELECT can assist in assessing whether further analyses are likely to provide additional insights. For two very similar groups, which cannot be discerned using all available non-constant genes, further analyses might not provide added value. When analysing treated and untreated cells, such a result might also indicate treatment failure or resistance, as described in the first scenario in the main text.

Second Scenario – rather similar groups in UMAP

The second scenario describes two rather similar groups according to UMAP visualisation, such as untreated cells compared to cells after 4 h of ARS-1620 treatment. However, despite the groups being not clearly separated in a UMAP plot, which might be due to the short treatment duration of 4 h, both classical analysis methods and gSELECT can identify differentially expressed genes between the two groups. This is already indicated by the high balanced accuracy of over 97% when analysing all non-constant genes, and by the high accuracy of over 95% when analysing the ten top-ranked MI genes. In this analysis, using gSELECT as a pre-analysis tool would indicate that further analysis is very likely to yield interesting results. To avoid missing potentially relevant genes due to false negative results, we

recommend performing several sweeps and consider the mutual information. Additionally, a second analysis method, such as Wilcoxon, can be employed.

In situations where the UMAP visualisation of two groups of interest shows no clear separation, gSELECT can be used as a quick and easy-to-use pre-analysis tool, indicating whether further analysis is advisable. gSELECT is a user-friendly and easy-to-use tool, with a reliability that is compatible with other standard analysis methods, which typically require more programming effort. Pre-analysis using gSELECT can help to determine whether further analysis of a dataset with no clear separation in the UMAP visualisation is advisable, which can save time and resources.

Third Scenario – distinct differences in UMAP

To demonstrate all features of gSELECT in a case study, we also analysed two distinct groups of H358 cells: untreated H358 cells, and H358 cells after treatment for 72 h; dataset GSE137912 published by Xue et al. (2020) [39], which are clearly separated in the UMAP visualisation. The authors used single cell sequencing to analyse the response of different isogenic cell populations (three $KRAS^{G12C}$ mutant tumour cell models: H358, H2122 and SW1573) to $KRAS^{G12C}$ inhibitor treatment and report that some cancer cells were able to bypass inhibition by producing new $KRAS^{G12C}$ in its active, drug-insensitive state, while cells with the inactive, drug-sensitive $KRAS^{G12C}$ state remained sensitive to treatment [39]. The resulting single cell dataset, GSE137912, has also been used to demonstrate other machine learning methods, such as the innovative machine learning based framework introduced by Lin et al. (2023) [62], who focused on finding differences in gene expression between cells showing no response or a positive response following KRAS inhibitor treatment, aiming to gain further insight into KRAS inhibitor resistance [62]. Their machine learning framework was designed to classify cells as proliferative or quiescent following KRAS inhibitor treatment and to identify key genes for the classification by using a combination of feature ranking, incremental feature selection, and classification algorithms [62].

While these studies focused on resistant cells, we applied gSELECT to compare untreated and treated H358 cells. Our aim was to identify genes with high mutual information and predictive power in distinguishing between the two conditions, and to demonstrate gSELECT's core functions. The resulting

MI genes indicate that ARS-1620 treatment appeared to affect several genes which could indicate a positive treatment response, e.g., the downregulation of genes associated with glycolysis and the Warburg effect. That the cells were affected by ARS-1620 treatment was also observed by Xue et al. (2020) [39]. However, the approach is not limited to this comparison. Any grouping can be analysed, provided that metadata are available in the .h5ad file or as a labelled .csv file (containing e.g., two groups with distinct labels, such as "0" or "untreated" for untreated cells and "1" or "treated" for treated cells, prepared according to the description in Caliskan et al. (2023) [11]). This enables the identification of predictive genes across diverse conditions.

Therefore, we performed several different analyses, which are all possible with gSELECT: (1) analysis of all available, non-constant genes, which is suitable as a first pre-analysis step, (2) analysis of the top-ranked MI genes, which is suitable as a second pre-analysis step and can indicate potential genes of interest, (3) analysis of the predictive power of custom genes of interest, which is suitable as an additional analysis step, e.g. before setting up further laboratory experiments, and can be performed during pre-analysis (e.g. to evaluate potential genes of interest according to literature research) or as post-analysis step to confirm newly identified genes of interest, and (4) calculating the predictive power of different gene combinations, which can indicate gene combinations with especially high predictive accuracy, which could be of special interest for deeper biological understanding or as potential candidates for treatment approaches targeting more than one gene (e.g. combination treatments). Details on the analysis options can be found in the Tutorial included in the Supplementary Data.

A potential use case for such a gSELECT pre-analysis is analysing sequencing data (newly generated sequencing data for a specific research question or a (publicly) available dataset addressing a similar research question), to narrow down candidate genes for detailed experimental investigation. Additionally, gSELECT can complement classical analysis methods by providing further validation of gene candidates identified through other approaches, confirming their biological relevance *in silico*.

Besides confirming the respective MI genes using other standard analysis methods such as Wilcoxon, DESeq2 [21], and MAST [22], we also performed Survival Analyses of the top-ranked MI genes using TCGA-LUAD (The Cancer Genome Atlas Program Lung Adenocarcinoma) data [23] to validate their

clinical relevance, and Enrichment Analysis, to validate their potential biological relevance. The MI genes, which could all be confirmed with further *in silico* analyses have already been associated with lung cancer or with pathways also associated with lung cancer. This demonstrates that gSELECT can identify genes of interest that might be promising biomarkers or therapeutic targets.

Enrichment analysis with the top 25 MI genes after 72 h showed that hypoxia and glycolysis were the two top-ranked significant pathways. Genes associated with glycolysis Pathway (*PKM*, *ALDOA*, *TPI1*, and *LDHA*) were also assessed for their predictive value. The high predictive value of these genes (over 90%) indicates that their expression appears to be highly affected by ARS-1620 treatment. Therefore, these genes could contribute to the difference between the two conditions.

KRAS mutations drive alterations in metabolic pathways, including enhanced nutrient uptake and elevated glutaminolysis as well as increased glycolysis and elevated synthesis of fatty acids nucleotides [63]. Tumour metabolism is a critical hallmark of cancer and significantly contributes to the development and progression of tumours [63]. The Warburg effect, named after Otto Warburg, the winner of the Nobel Prize in Medicine in 1931 [64], is a well-known tumour metabolism [63] and describes the alterations in tumour cells regarding energy processing [64]. Instead of relying on oxidative phosphorylation in the mitochondria, tumour cells preferentially utilize glycolysis for energy production, which is followed by lactate fermentation and lactate secretion, even if oxygen is present [63].

Although the Warburg effect results in less ATP per glucose molecule than oxidative phosphorylation, it operates much faster and therefore supports the accelerated growth and proliferation of cancer cells [63]. In metabolic reprogramming, KRAS enhances glycolysis, enabling uncontrolled proliferation and growth [63]. Due to the hypoxia microenvironment and the Warburg effect, tumour cells predominantly undergo aerobic glycolysis [65]. Lactate dehydrogenase A (LDHA), which is among the MI genes and downregulated in H358 cells after ARS-1620 treatment, has been shown to link the Warburg effect and the activity of phosphatidylinositol three kinase (PI3K) [65, 66]. Via the Warburg metabolism, cancer cells can maintain the biological activity of the PI3K signalling pathway, which ensures the continuous proliferation of tumour cells [65]. Therefore, inhibiting the “Warburg switch” of LDHA and thus

inhibiting the growth of cancer cells has been suggested as a promising treatment method for cancer [65]. In different cancer types, the Warburg effect can be promoted by different subtypes of certain enzymes, including LDHA, Aldolase, Fructose-Bisphosphate A (ALDOA), and triosephosphate isomerase (TPI, also TPI1) [65], all of which are MI genes and downregulated after ARS-1620 treatment. LDHA is involved in catalysing the interconversion between pyruvate and lactate in the cytoplasm of tumour cells [65]. In multiple cancers, LDHA is aberrantly highly expressed, and known to promote the malignant progression of tumours due to facilitating glycolysis by converting pyruvate to lactate [53]. Additionally, it is involved in several Hallmarks of Cancer [53]. As discussed in detail by Feng et al. (2018), LDHA can trigger angiogenesis, enhance metastasis and cancer cell invasion, promote the proliferation of cancer cells and maintain their survival, and can assist cancer cells in immune escape [53].

While non-tumour lung tissues were reported to show negative LDHA expression, almost 90% of NSCLC patients were reported to be positive for LDHA, with the staining intensity for LDHA being positively related to the histological type and lymph node metastasis, which indicates a prognostic value for LDHA in NSCLC [53].

Silencing LDHA expression is rarely harmful to normal cells but inhibits tumour growth, migration and cell proliferation in tumour models, making LDHA to a potential antitumor target [53]. In a mouse model created by Xie et al. (2014), reduced expression of LDHA in adult mice resulted in no obvious toxicities but non-lethal anaemia, while reduced expression in highly glycolytic tumours, including tumours with oncogenic KRAS-mutations, resulted in decreased tumorigenesis and regression of established tumours appeared to affect cancer stem cells, which are not typically targeted by cancer therapies [55]. Their work also demonstrated the critical role of LDHA in tumour progression [55]. Furthermore, inhibiting LDHA has been shown to change the metabolism of cancer cells from glycolysis to mitochondrial respiration and to enhance the effectiveness of target drugs, chemotherapy, and radiotherapy [54]. This is especially interesting as the 5-year survival rate of stage III lung cancer patients after chemo-radiotherapy is still relatively low with approximately 15-20% and radioresistance appears to widely exist in NSCLC patients [54]. LDHA has also been suggested as promising target for overcoming paclitaxel resistance, since inhibiting LDHA resensitized paclitaxel-resistant cells to the

drug, while upregulation of LDHA increases glucose utilization and lactate production, which has been reported to promote occurrence and metastasis of pancreatic adenocarcinoma [65].

ALDOA is known to be highly expressed in various cancer types, acting as a glycolytic enzyme [65]. It catalyses fructose-1,6-diphosphate to dihydroxyacetone phosphate and glyceraldehyde 3-phosphate during the transformation of glucose to pyruvate and has been suggested as therapeutic target in osteosarcoma [65]. TPI1, which is upregulated in tissues with increased ability for aerobic glycolysis, is involved in the catalysis of dihydroxyacetone phosphate to glyceraldehyde 3-phosphate [65].

The PKM gene encodes for PKM1 and PKM2, two isoforms resulting from mutually exclusive alternative splicing of PKM [67], which show a splicing difference but perform the same catalytic function [68]. The key glycolytic enzyme pyruvate kinase M2 (PKM2) is upregulated in cancer cells [67] and the only subtype of pyruvate kinase expressed in tumour tissues, and it has been reported that a knock down of PKM2 results in a reduced production of lactic acid coupled with increased oxygen consumption, which ultimately leads to a reversal of the Warburg effect [65]. In human lung cancer cells, the Warburg effect can be inhibited by replacing PKM2 with PKM1 [67]. PKM2 regulates the expression of cyclin D1, which is involved in cell cycle progression, and can upregulate the expression of c-Myc, which in turn promotes the expression of glycolytic enzyme genes and aerobic glycolysis [67]. This makes PKM2 essential for cell cycle progression, tumorigenesis, and the Warburg effect [67].

In summary, the MI genes associated with glycolysis appear to be affected by ARS-1620 treatment, as they are downregulated after treatment. Since downregulation of these genes is favourable for survival, which can also be seen in the respective survival analyses, these results indicate that ARS-1620 treatment for 72 h was effective in H358 cells. However, although ARS-1620 treatment is initially effective on H358 cells [69], continuous ARS-1620 treatment (with 1.0 µM ARS-1620) induces acquired resistance within 2-3 weeks [69], indicating the need for combination treatments which are already being studied [69, 70].

All analyses were performed *in silico*, which is a common and essential first step in modern gene prioritisation workflows. The consistent and literature-supported identification of MI genes by gSELECT highlights the method's potential to guide subsequent experimental efforts. Although further

in vitro and *in vivo* validation will ultimately be required, as with any computational approach, our method enables a focused selection of promising candidates, thereby helping to streamline and rationalise subsequent experimental validation efforts. Since the primary objective of this study was to evaluate whether gSELECT effectively identifies relevant genes for the differences between treated and untreated cells, these results seem to confirm that the identified MI genes indeed have also predictive power and help to evaluate important genes which can be further analysed.

As gSELECT offers this functionality for both the top-ranked MI genes and custom-selected genes, promising gene combinations for combination therapies can be calculated for various genes of interest to predict the gene combination that is most related to the difference of interest based on predictive power, independent of whether they were among the most-relevant genes of the analysed data according to some ranking method, are based on literature research or selected because of available treatment options targeting these genes. This can facilitate finding new combination therapies and evaluate the potential of different combination therapies *in silico*, which might aid in pre-selecting potential combination treatments for laboratory studies, potentially saving costs and time.

gSELECT offers four core functionalities, which could be demonstrated using single cell sequencing data of a publicly available lung cancer dataset. It can help to evaluate which comparisons within a dataset are suitable for subsequent DE analysis and verify potentially relevant genes using machine learning and mutual information. The resulting mutual information genes were verified using several standard analysis methods. Literature research demonstrated the reliability of gSELECT in finding important genes. The automated workflow is user-friendly and can easily be integrated in any standard single cell analysis workflow using h5ad files.

This allows users to support their own hypotheses by validating genes of interest, as potentially relevant genes that are well-related to the phenotypic differences will most likely have a high predictive power (recognisable by a relatively high balanced accuracy). Furthermore, the predictive power of custom gene combinations can be calculated, which might aid in the development of combination therapies.

References

1. Edgar, R., M. Domrachev, and A.E. Lash, *Gene Expression Omnibus: NCBI gene expression and hybridization array data repository.* Nucleic Acids Research, 2002. **30**(1): p. 207-210.
2. Barrett, T., et al., *NCBI GEO: archive for functional genomics data sets—update.* Nucleic Acids Research, 2013. **41**(D1): p. D991-D995.
3. Satija, R., P. Hoffman, and A. Butler, *SeuratData: Install and Manage Seurat Datasets*. 2025.
4. Wolf, F.A., P. Angerer, and F.J. Theis, *SCANPY: large-scale single-cell gene expression data analysis.* Genome Biology, 2018. **19**(1): p. 15.
5. Wolock, S.L., R. Lopez, and A.M. Klein, *Scrublet: Computational Identification of Cell Doublets in Single-Cell Transcriptomic Data.* Cell Systems, 2019. **8**(4): p. 281-291.e9.
6. The pandas development team, *pandas-dev/pandas: Pandas*. 2024.
7. Virtanen, P., et al., *SciPy 1.0: fundamental algorithms for scientific computing in Python.* Nature Methods, 2020. **17**(3): p. 261-272.
8. Harris, C.R., et al., *Array programming with NumPy.* Nature, 2020. **585**(7825): p. 357-362.
9. Hunter, J.D., *Matplotlib: A 2D Graphics Environment.* Computing in Science & Engineering, 2007. **9**(3): p. 90-95.
10. Dyer, S.C., et al., *Ensembl 2025.* Nucleic Acids Research, 2025. **53**(D1): p. D948-D957.
11. Caliskan, A., et al., *Optimized cell type signatures revealed from single-cell data by combining principal feature analysis, mutual information, and machine learning.* Computational and Structural Biotechnology Journal, 2023. **21**: p. 3293-3314.
12. Satija, R., et al., *Spatial reconstruction of single-cell gene expression data.* Nature Biotechnology, 2015. **33**(5): p. 495-502.
13. Hao, Y., et al., *Dictionary learning for integrative, multimodal and scalable single-cell analysis.* Nature Biotechnology, 2024. **42**(2): p. 293-304.
14. R Core Team, *R: A Language and Environment for Statistical Computing; Vienna, Austria*. 2021, R Foundation for Statistical Computing, : Vienna, Austria.
15. Ushey, K., J. Allaire, and Y. Tang, *reticulate: Interface to 'Python'*. 2024.
16. Hao, Y., et al., *Integrated analysis of multimodal single-cell data.* Cell, 2021. **184**(13): p. 3573-3587.e29.
17. Stuart, T., et al., *Comprehensive Integration of Single-Cell Data.* Cell, 2019. **177**(7): p. 1888-1902.e21.
18. Butler, A., et al., *Integrating single-cell transcriptomic data across different conditions, technologies, and species.* Nature Biotechnology, 2018. **36**(5): p. 411-420.
19. Wickham, H., *ggplot2: Elegant Graphics for Data Analysis*. 2016: Springer-Verlag New York.
20. Hadley, W., et al., *Welcome to the Tidyverse.* Journal of Open Source Software, 2019. **4**(43): p. 1686.
21. Love, M.I., W. Huber, and S. Anders, *Moderated estimation of fold change and dispersion for RNA-seq data with DESeq2.* Genome Biology, 2014. **15**(12): p. 550.
22. Finak, G., et al., *MAST: a flexible statistical framework for assessing transcriptional changes and characterizing heterogeneity in single-cell RNA sequencing data.* Genome Biology, 2015. **16**(1): p. 278.
23. Collisson, E.A., et al., *Comprehensive molecular profiling of lung adenocarcinoma.* Nature, 2014. **511**(7511): p. 543-550.
24. Yao, Z., et al., *A taxonomy of transcriptomic cell types across the isocortex and hippocampal formation.* Cell, 2021. **184**(12): p. 3222-3241.e26.
25. Virshup, I., et al., *anndata: Access and store annotated data matrices.* Journal of Open Source Software, 2024. **9**(101): p. 4371.
26. Collette, A., et al., *h5py/h5py: 3.7.0*. 2022, Zenodo.
27. Kibirige, H., *scikit-misc*. 2023.
28. Satija, R., Satija Lab, and Collaborators. *Introduction to scRNA-seq integration*. Seurat Vignette 2023 [cited 2025 23.07.2025]; Available from: https://satijalab.org/seurat/articles/integration_introduction.

29. Satija Lab. *Mapping and annotating query datasets*. 2023 23 June 2025].
30. Rasbach, L., et al., *An orchestra of machine learning methods reveals landmarks in single-cell data exemplified with aging fibroblasts.* PLOS ONE, 2024. **19**(4): p. e0302045.
31. Segerstolpe, Å., et al., *Single-Cell Transcriptome Profiling of Human Pancreatic Islets in Health and Type 2 Diabetes.* Cell Metabolism, 2016. **24**(4): p. 593-607.
32. Prasad, Rashmi B. and L. Groop, *Single-Cell Sequencing of Human Pancreatic Islets—New Kids on the Block.* Cell Metabolism, 2016. **24**(4): p. 523-524.
33. van Gurp, L., et al., *Generation of human islet cell type-specific identity genesets.* Nature Communications, 2022. **13**(1): p. 2020.
34. van Gurp, L., et al., *Author Correction: Generation of human islet cell type-specific identity genesets.* Nature Communications, 2024. **15**(1): p. 2574.
35. Dorrell, C., et al., *Transcriptomes of the major human pancreatic cell types.* Diabetologia, 2011. **54**(11): p. 2832-2844.
36. Dorrell, C., et al., *Erratum to: Transcriptomes of the major human pancreatic cell types.* Diabetologia, 2013. **56**(5): p. 1192-1192.
37. Su, Y., et al., *Novel function of transthyretin in pancreatic alpha cells.* FEBS Letters, 2012. **586**(23): p. 4215-4222.
38. Dekki, N., et al., *Transthyretin binds to glucose-regulated proteins and is subjected to endocytosis by the pancreatic β-cell.* Cellular and Molecular Life Sciences, 2012. **69**(10): p. 1733-1743.
39. Xue, J.Y., et al., *Rapid non-uniform adaptation to conformation-specific KRAS(G12C) inhibition.* Nature, 2020. **577**(7790): p. 421-425.
40. Satija, R., Satija Lab, and Collaborators. *Differential expression testing*. Seurat Vignette 2024 [cited 2025 13.03.2025]; Available from: https://satijalab.org/seurat/articles/de_vignette.html.
41. Lin, Y., et al., *scClassify: sample size estimation and multiscale classification of cells using single and multiple reference.* Molecular Systems Biology, 2020. **16**(6): p. e9389.
42. Pedregosa, F., et al., *Scikit-learn: Machine Learning in Python.* Journal of Machine Learning Research, 2012. **12**.
43. Jensen, L.J., et al., *STRING 8—a global view on proteins and their functional interactions in 630 organisms.* Nucleic Acids Research, 2009. **37**(suppl_1): p. D412-D416.
44. Szklarczyk, D., et al., *The STRING database in 2021: customizable protein-protein networks, and functional characterization of user-uploaded gene/measurement sets.* Nucleic acids research, 2021. **49**(D1): p. D605-D612.
45. Szklarczyk, D., et al., *The STRING database in 2023: protein-protein association networks and functional enrichment analyses for any sequenced genome of interest.* Nucleic acids research, 2023. **51**(D1): p. D638-D646.
46. Szklarczyk, D., et al., *The STRING database in 2023: protein–protein association networks and functional enrichment analyses for any sequenced genome of interest.* Nucleic Acids Research, 2023. **51**(D1): p. D638-D646.
47. Li, J., et al., *S100A10 promotes cancer metastasis via recruitment of MDSCs within the lungs.* OncoImmunology, 2024. **13**(1): p. 2381803.
48. Sato, K., et al., *S100A10 upregulation associates with poor prognosis in lung squamous cell carcinoma.* Biochemical and Biophysical Research Communications, 2018. **505**(2): p. 466-470.
49. Saed, L., et al. *Prognostic Significance of HMGA1 Expression in Lung Cancer Based on Bioinformatics Analysis*. International Journal of Molecular Sciences, 2022. **23**, DOI: 10.3390/ijms23136933.
50. Ma, Y., et al., *HMGA1 is a Prognostic Biomarker and Correlated with Glycolysis in Lung Adenocarcinoma.* Journal of Cancer, 2024. **15**(10): p. 2913-2927.
51. Bian, T., et al., *Prognostic biomarker TUBA1C is correlated to immune cell infiltration in the tumor microenvironment of lung adenocarcinoma.* Cancer Cell International, 2021. **21**(1): p. 144.
52. Fan, X., et al., *Identification of TUBA1C as a prognostic biomarker and associated with immune cells infiltration in human tumors.* PREPRINT (Version 1) available at Research Square, 2022.

53. Feng, Y., et al., *Lactate dehydrogenase A: A key player in carcinogenesis and potential target in cancer therapy.* Cancer Medicine, 2018. **7**(12): p. 6124-6136.
54. Yang, Y., et al., *Targeting lactate dehydrogenase a improves radiotherapy efficacy in non-small cell lung cancer: from bedside to bench.* Journal of Translational Medicine, 2021. **19**(1): p. 170.
55. Xie, H., et al., *Targeting Lactate Dehydrogenase-A Inhibits Tumorigenesis and Tumor Progression in Mouse Models of Lung Cancer and Impacts Tumor-Initiating Cells.* Cell Metabolism, 2014. **19**(5): p. 795-809.
56. Pang, X.-L., et al., *Tubulin Alpha-1b as a Potential Biomarker for Lung Adenocarcinoma Diagnosis and Prognosis.* Technology in Cancer Research & Treatment, 2023. **22**: p. 15330338231178391.
57. Qi, J., et al., *TUBA1B as a novel prognostic biomarker correlated with immunosuppressive tumor microenvironment and immunotherapy response.* Frontiers in Pharmacology, 2025. **16**.
58. Gocheva, V., et al., *Quantitative proteomics identify Tenascin-C as a promoter of lung cancer progression and contributor to a signature prognostic of patient survival.* Proceedings of the National Academy of Sciences, 2017. **114**(28): p. E5625-E5634.
59. Kuhn, M., *Building Predictive Models in R Using the caret Package.* Journal of Statistical Software, 2008. **28**(5): p. 1 - 26.
60. Molbiotools. *Compare Lists - Multiple List Comparator*. 2025 [cited 2025 15 July 2025]; Available from: https://molbiotools.com/listcompare.php.
61. Robin, X., et al., *pROC: an open-source package for R and S+ to analyze and compare ROC curves.* BMC Bioinformatics, 2011. **12**(1): p. 77.
62. Lin, X., et al., *Identifying genes associated with resistance to KRAS G12C inhibitors via machine learning methods.* Biochimica et Biophysica Acta (BBA) - General Subjects, 2023. **1867**(12): p. 130484.
63. Ma, Q., et al., *The roles of KRAS in cancer metabolism, tumor microenvironment and clinical therapy.* Molecular Cancer, 2025. **24**(1): p. 14.
64. Aguilera, O., et al., *Vitamin C uncouples the Warburg metabolic switch in KRAS mutant colon cancer.* Oncotarget, 2016. **7**(30).
65. Liao, M., et al., *Targeting the Warburg effect: A revisited perspective from molecular mechanisms to traditional and innovative therapeutic strategies in cancer.* Acta Pharmaceutica Sinica B, 2024. **14**(3): p. 953-1008.
66. Xu, K., et al., *Glycolysis fuels phosphoinositide 3-kinase signaling to bolster T cell immunity.* Science, 2021. **371**(6527): p. 405-410.
67. Yang, W. and Z. Lu, *Nuclear PKM2 regulates the Warburg effect.* Cell Cycle, 2013. **12**(19): p. 3343-3347.
68. Zhang, Z., et al., *PKM2, function and expression and regulation.* Cell & Bioscience, 2019. **9**(1): p. 52.
69. Liu, Y., et al., *Enhancing the Therapeutic Efficacy of KRAS^{G12C} Inhibitors in Lung Adenocarcinoma Cell Models by Cotargeting the MAPK Pathway or HSP90.* Journal of Oncology, 2021. **2021**: p. 2721466.
70. Lou, K., et al., *KRASG12C inhibition produces a driver-limited state revealing collateral dependencies.* Science Signaling, 2019. **12**(583): p. eaaw9450.
