# Supplementary material for: gSELECT: A novel pre-analysis machine-learning library enabling early hypothesis testing and predictive gene selection in single-cell data
Source: Comput Struct Biotechnol J. 2025 Aug 5;27:3510–27. doi: 10.1016/j.csbj.2025.07.047 (PMC12354962; doi:10.1016/j.csbj.2025.07.047)
Supplement: Supplementary file 2 — Supplementary material [file mmc2.pdf]

Content

Tutorial for using gSelect 2

Downloading and Preparing a h5ad File 3

Installation of gSELECT 6

Installation of the development version 6

Running gSELECT 7

Exploring the Data 8

Optional UMAP Visualization 12

gSELECT Analysis 13

Optional: Creating a final Hold-out Test Set 13

When not using a final hold-out test set 14

Mutual Information 15

Analysis Option 1: Classification and Predictive Value of ALL genes 18

Short explanation to number_sweeps and max_iterations 19

Visualisation 21

Analysis Option 2: Classification and Predictive Power of SELECTED genes 22

Analysis Option 3: Finding the Optimal Number of Genes 25

Analysis Option 4: Calculating the Predictive Power of CUSTOM genes 28

Analysis Option 5: Explorative Analysis of Predictive Power 30

Final Notes 34

References 34

Tutorial for using gSelect

The tutorial provided with the manuscript is based on the version of gSELECT as of July 2025. The most up-to-date version of the tool is available on GitHub (https://github.com/CaliskanDeniz/gSELECT) and includes a detailed README file, which documents all current functions. Additionally, the repository contains a Jupyter notebook that can be executed directly and provides step-by-step guidance for using gSELECT. Should any changes or updates be made to the tool in the future, both the documentation and the notebook will be maintained accordingly to reflect the current state of the software. Further scripts, e.g., the scripts for data preparation, are available at https://github.com/AC-PHD/gselect-singlecell-scripts

For this tutorial, we will use a part of the single cell atlas of human and mouse white adipose tissue, which has been created by Emont et al. (2022) and is available via the Single Cell Portal (https://singlecell.broadinstitute.org/single_cell/study/SCP1376/a-single-cell-atlas-of-human-and-mouse-white-adipose-tissue#study-summary) and contains a variety of cell types from adipose tissue[1].

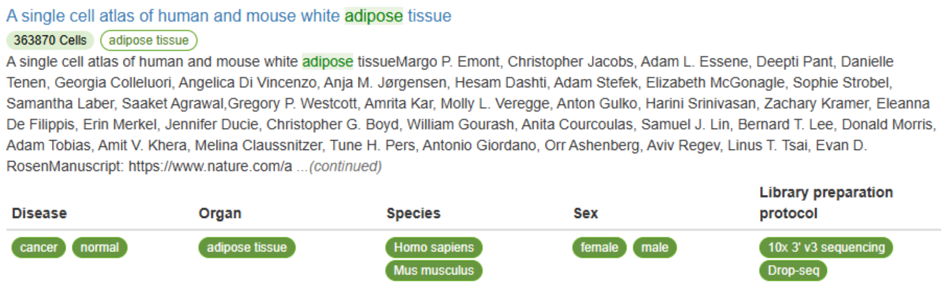

In order to download the samples, sign in is required, which might require an organization or university email address.

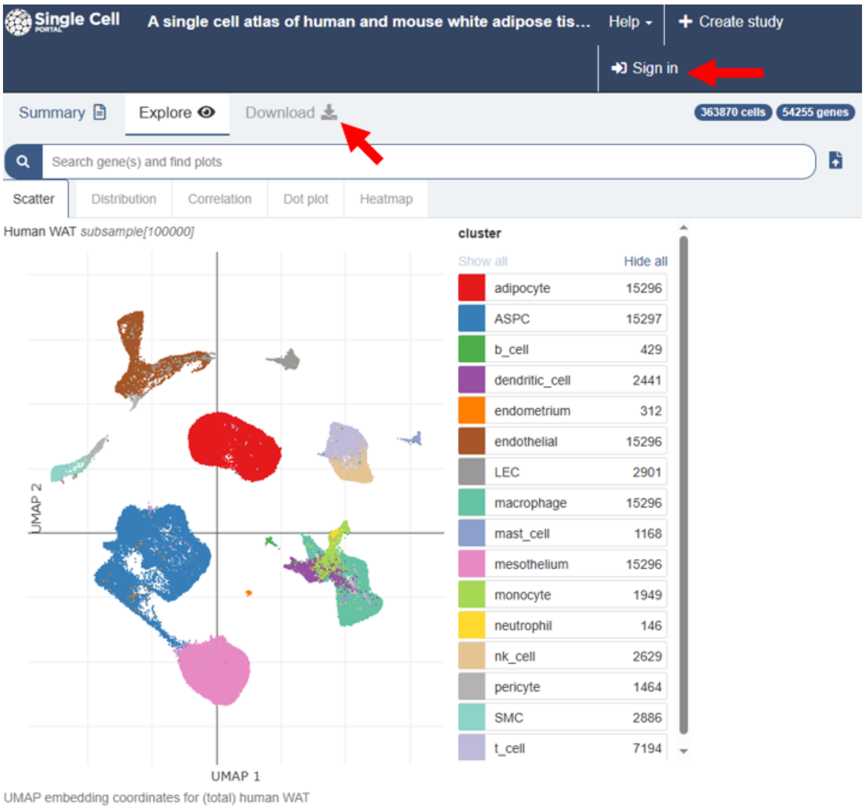

Downloading and Preparing a h5ad File

After signing in, the data is available for download. For this tutorial, we only need to download

- the barcode file (Hs10X.data.barcodes.tsv.gz)
- the feature file (Hs10X.data.features.tsv.gz)
- the matrix file (Hs10X.data.mtx.gz (normalized))
- the metadata file (metadata.tsv)

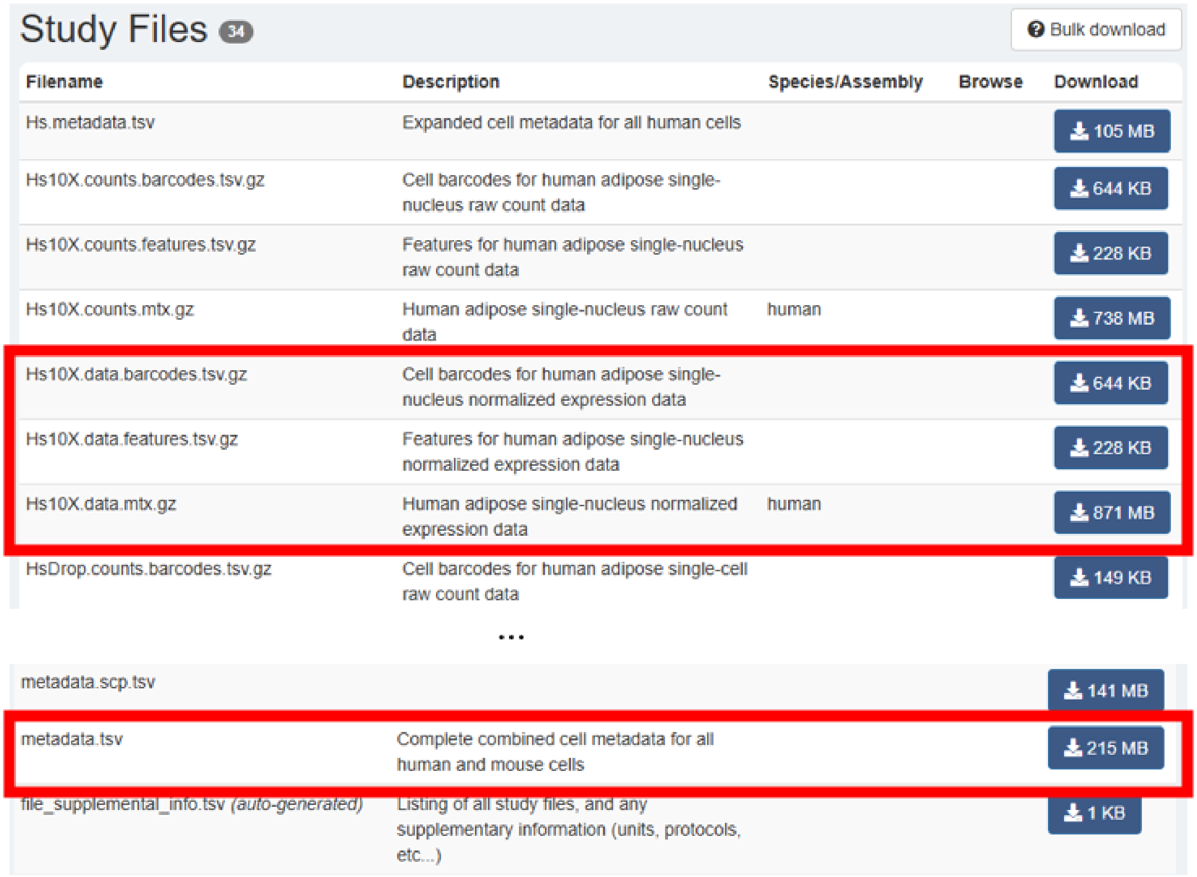

To prepare the data, it is best to save the downloaded files in the same directory as the mtx_to_h5ad.ipynb script, which can be found at https://github.com/AC-PHD/gselect-singlecell-scripts.

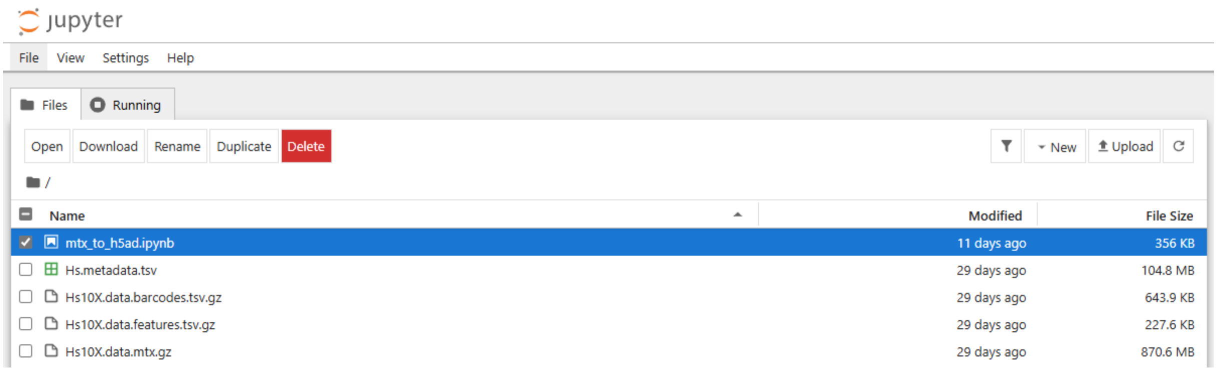

To run the script, open Jupyter Notebook and set the path for the respective files (upper red box) and the name for the output file (lower red box) before running the code.

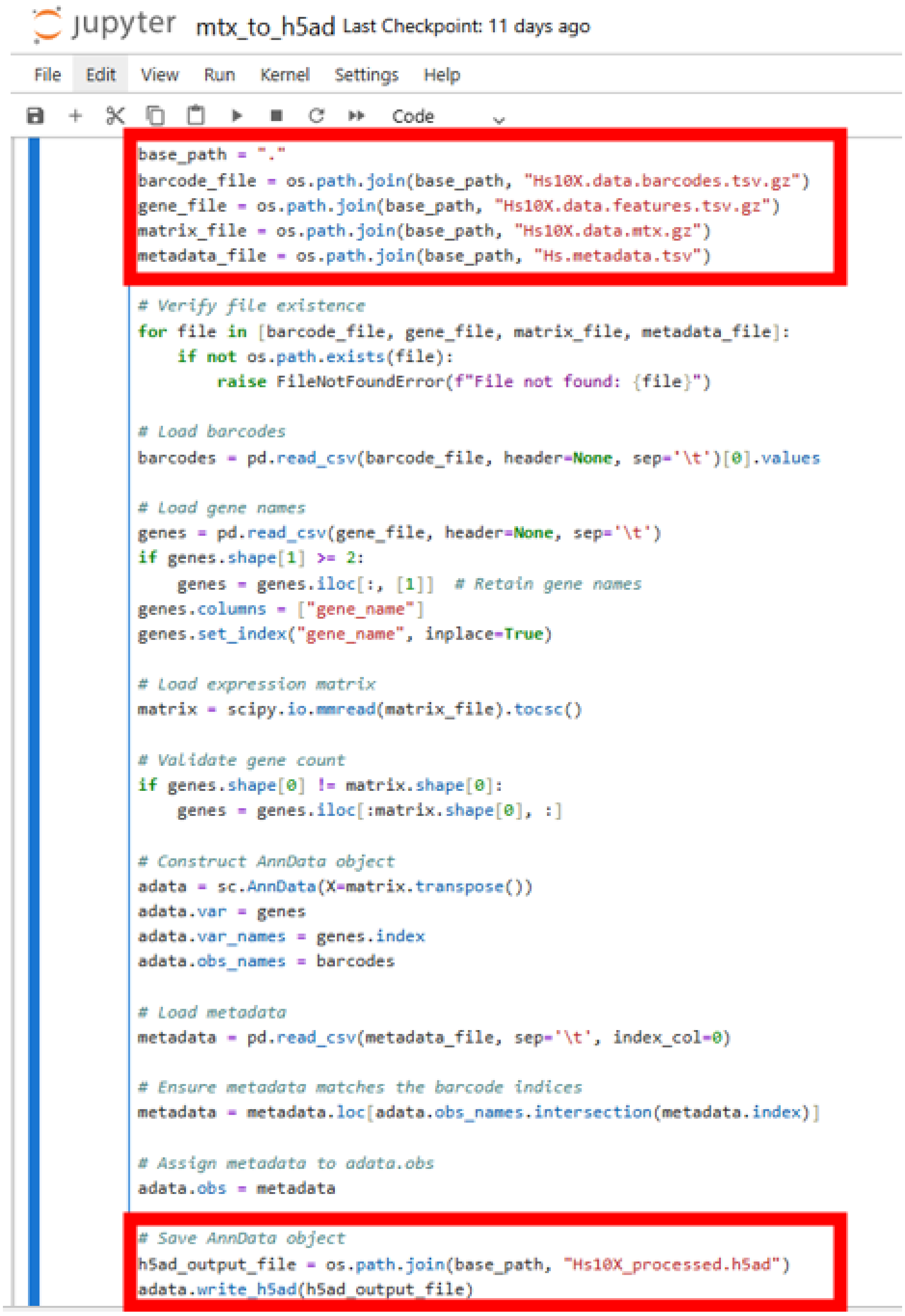


```python
base_path = "."
barcode_file = os.path.join(base_path, "Hs10X.data.barcodes.tsv.gz")
gene_file = os.path.join(base_path, "Hs10X.data.features.tsv.gz")
matrix_file = os.path.join(base_path, "Hs10X.data.mtx.gz")
metadata_file = os.path.join(base_path, "Hs.metadata.tsv")

# Verify file existence
for file in [barcode_file, gene_file, matrix_file, metadata_file]:
    if not os.path.exists(file):
        raise FileNotFoundError(f"File not found: {file}")

# Load barcodes
barcodes = pd.read_csv(barcode_file, header=None, sep='\t')[0].values

# Load gene names
genes = pd.read_csv(gene_file, header=None, sep='\t')
if genes.shape[1] >= 2:
    genes = genes.iloc[:, [1]]  # Retain gene names
genes.columns = ["gene_name"]
genes.set_index("gene_name", inplace=True)

# Load expression matrix
matrix = scipy.io.mmread(matrix_file).tocsc()

# Validate gene count
if genes.shape[0] != matrix.shape[0]:
    genes = genes.iloc[:matrix.shape[0], :]

# Construct AnnData object
adata = sc.AnnData(X=matrix.transpose())
adata.var = genes
adata.var_names = genes.index
adata.obs_names = barcodes

# Load metadata
metadata = pd.read_csv(metadata_file, sep='\t', index_col=0)

# Ensure metadata matches the barcode indices
metadata = metadata.loc[adata.obs_names.intersection(metadata.index)]

# Assign metadata to adata.obs
adata.obs = metadata

# Save AnnData object
h5ad_output_file = os.path.join(base_path, "Hs10X_processed.h5ad")
adata.write_h5ad(h5ad_output_file)
```

After the script has finished running, the h5ad file is saved in the directory and can be used in gSELECT. Alternatively, it is also possible to run gSELECT with any other h5ad file.

Installation of gSELECT

gSELECT has two installation options:

- pip install
- installing the development version via Git clone

For using pip install, run pip install gSELECT (e.g., in a Jupyter Notebook). After installing gSELECT via this option, the same notebook can be used for the subsequent data analysis. If you used pip install, you can skip the following section and continue with the section "Running gSELECT"

Installation of the development version

To install the development version of gSELECT, you can download the necessary files at https://github.com/CaliskanDeniz/gSELECT with

git clone git@github.com:CaliskanDeniz/gSELECT.git which will download the gSELECT directory.

For the subsequent analysis, it is best to copy the h5ad file into the same directory that contains the subdirectory gSELECT:

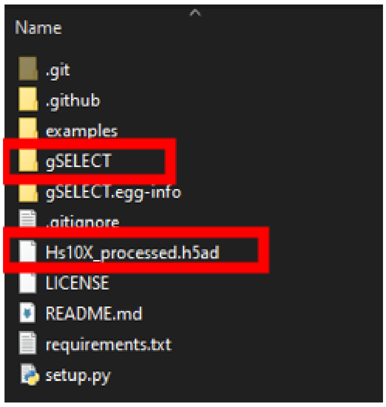

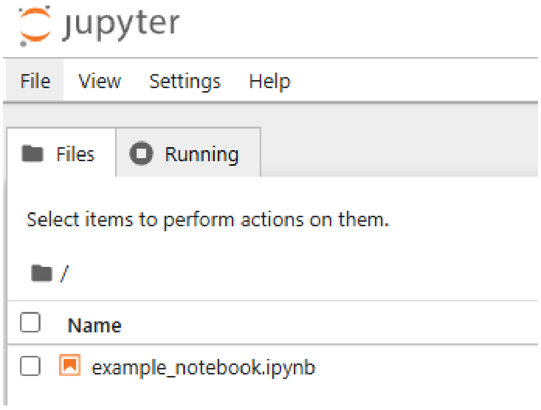

To run the example code, open Jupyter Notebook in the subdirectory "examples", and run the example script example_notebook.ipynb (available at https://github.com/CaliskanDeniz/gSELECT)

Running gSELECT

How to run gSELECT is independent of the installation method, therefore the following explanations apply to both the development and the normal version of gSELECT.

When running gSELECT for the first time, the dependencies need to be installed by running

pip install -e ../.

This needs to be performed only once.

Additionally, the required libraries need to be imported

import gSELECT.io as gsio

import gSELECT.feature_selection as gsfs

import gSELECT.classification as gsc

import gSELECT.visualization as gsv

The next section allows entering the information required for the analysis:

- the file path to the h5ad file,
- a custom output directory (optional, gSELECT will create and use the directory "output" by default),

filepath = "../Hs10x_processed.h5ad"

output_path = "output"

Exploring the Data

To compare two different groups additional information is required (which can also be obtained by exploring the h5ad file)

- the filter column containing the information on the respective groups that are being analysed
- the filter values (e.g. “treated” and “untreated”, or “cell type of interest 1” and “cell type of interest 2”

Knowing the respective filter column before the analysis is not required, as gSELECT allows exploring the data with the explore_h5ad function.

gsio.explore_h5ad(filepath)

Running the explore function will result in all available information of the h5ad file being displayed:

```
Loaded H5AD file: ../Hs10X_processed.h5ad
Shape of the data (cells, genes): (137684, 29093)

--- Summary of `adata.obs` (cell metadata) ---
<class 'pandas.core.frame.DataFrame'>
Index: 137684 entries, Hs_OAT_12-1_AAACCCAAGCACAAAT to Hs_SAT_11-1_TTTGTTGGTGCAGATG
Data columns (total 50 columns):
 #   Column                                    Non-Null Count   Dtype
---  ------                                    --------------   -----
 0   biosample_id                              137684 non-null  category
 1   biosample_type                            137684 non-null  category
 2   library_preparation_protocol__ontology_id 137684 non-null  category
 3   library_preparation_protocol__ontology_label 137684 non-null  category
 4   donor_id                                  137684 non-null  category
 5   species__ontology_id                      137684 non-null  category
 6   species__ontology_label                   137684 non-null  category
 7   organ__ontology_id                        137684 non-null  category
 8   organ__ontology_label                     137684 non-null  category
 9   tissue__ontology_id                       137684 non-null  category
 10  tissue__ontology_label                    137684 non-null  category
 11  tissue__type__ontology_id                 137684 non-null  category
 12  tissue__type__ontology_label              137684 non-null  category
 13  depot__ontology_id                        137684 non-null  category
 14  depot__ontology_label                     137684 non-null  category
 15  sex__ontology_id                          137684 non-null  category
 16  sex__ontology_label                       137684 non-null  category
 17  race__ontology_id                         137684 non-null  category
 18  race__ontology_label                      137684 non-null  category
 19  ethnicity__ontology_id                    137684 non-null  category
 20  ethnicity__ontology_label                 137684 non-null  category
[illegible]
```

Including information on the different columns of the metadata. Each of these columns can be selected for the subsequent analysis, e.g., information on patient data

```
Column: development_stage__ontology_label | Unique values (11):
['36-year-old human stage', '24-year-old human stage', '68-year-old human stage', '29-year-old human stage', '57-year-old human stage', '51-year-o
man stage', '53-year-old human stage', '41-year-old human stage', '73-year-old human stage', '35-year-old human stage']
Categories (11, object): ['24-year-old human stage', '29-year-old human stage', '35-year-old human stage', '36-year-old human stage', ..., '57-yea
human stage', '58-year-old human stage', '68-year-old human stage', '73-year-old human stage']
...
```

Or the age group (for instance to analyse differences in adipose tissue between different age groups, such as ‘30-40’ and ‘70-80’)

```
Column: organism_age__group | Unique values (6):
['30-40', '20-30', '60-70', '50-60', '40-50', '70-80']
Categories (6, object): ['20-30', '30-40', '40-50', '50-60', '60-70', '70-80']
```

Any information available in the metadata of the h5ad file can be selected for a subsequent comparison.

Here, we chose the cell type column:

```
Column: cell_type__custom | Unique values (16):
['ASPC', 'mesothelium', 'pericyte', 'adipocyte', 'macrophage', 'endothelial', 'LEC', 'monocyte', 't_cell', 'dendritic_cell']
Categories (16, object): ['ASPC', 'LEC', 'SMC', 'adipocyte', ..., 'neutrophil', 'nk_cell', 'pericyte', 't_cell']
...
```

To save calculation time for the tutorial, we chose dendritic cells and monocytes due to the relatively small number of cells in the respective groups. Comparing bigger groups is also possible but increases the computational time.

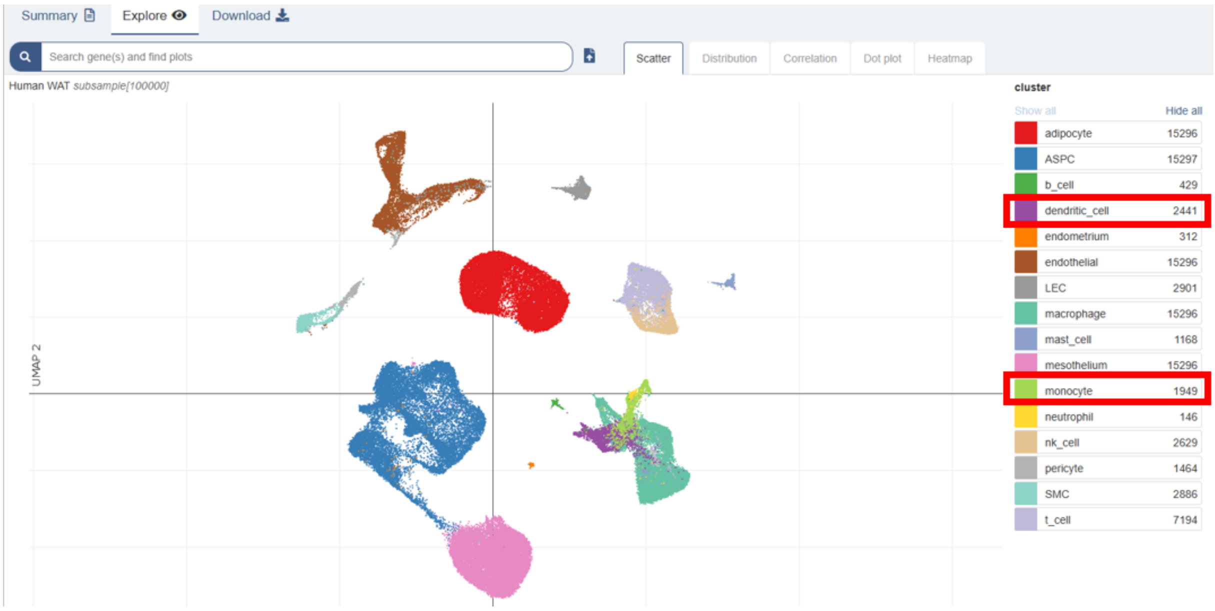

The name of the column is required as 'filter_column', while the two groups (which have to be part of the filter column) are entered as 'filter_values'. As long as two different groups are selected, it is not important which of the groups is used as 'value_zero' and which of the groups is used as 'value_one'

```
Column: cell_type__custom Unique values (16):
['ASPC', 'mesothelium', 'pericyte', 'adipocyte', 'macrophage', 'endothelial', 'LEC', 'monocyte', 't_cell', 'dendritic_cell'
Categories (16, object): ['ASPC', 'LEC', 'SMC', 'adipocyte', ..., 'neutrophil', 'nk_cell', 'pericyte', 't_cell']
...
```

```
filter_column = "your_filtercolumn"
filter_values=['value_zero', 'value_one']
```

→

```
filter_column = "cell_type__custom"
filter_values=['dendritic_cell', 'monocyte']
```

After that the h5ad file can be loaded with the needed information

filter_column = "cell_type__custom"

filter_values=['dendritic_cell', 'monocyte']

genes,data = gsio.load_h5ad(filepath,filter_column=filter_column,

filter_values=filter_values)

The output

```
Loaded H5AD file: ../Hs10X_processed.h5ad
```

indicates that the data was successfully loaded in gSELECT. While loading version-dependent warnings might appear, however, if the file path appears at ‘Loaded H5AD file:’ the data is correctly loaded into gSELECT.

Note:

If you want to analyse data which you have prepared as a csv you only need to load your csv

genes,data = gsio.load(filepath)

Optional UMAP Visualization

With this information about the respective groups, it is also possible to create a UMAP visualization of the selected group, using the script available at https://github.com/AC-PHD/gselect-singlecell-scripts. The script only requires the h5ad file, the target column, and the two groups of interest.

```
import scanpy as sc
import matplotlib.pyplot as plt

def load_and_process_h5ad(file_path: str, filter_column: str, filter_values: list) -> sc.AnnData:
    adata = sc.read_h5ad(file_path)
    if filter_column not in adata.obs.columns:
        raise ValueError(f"Column '{filter_column}' not found in `adata.obs`. Available columns: {adata.obs.columns.tolist()}")

    adata = adata[adata.obs[filter_column].isin(filter_values)].copy()
    return adata

def perform_umap(adata: sc.AnnData) -> sc.AnnData:
    if "X_pca" not in adata.obsm:
        sc.tl.pca(adata)
    sc.pp.neighbors(adata)
    sc.tl.umap(adata)
    return adata

def plot_umap(adata: sc.AnnData, color_column: str) -> None:
    if color_column not in adata.obs.columns:
        raise ValueError(f"Column '{color_column}' not found in `adata.obs`. Available columns: {adata.obs.columns.tolist()}")

    sc.pl.umap(adata, color=color_column, show=True)

# --- Inputs ---
file_path = "../Hs10X_processed.h5ad"  # Path to H5AD file
target_column = "cell_type__custom"  # Column to filter
filter_values = ['dendritic_cell', 'monocyte']  # Values

# Pipeline
adata = load_and_process_h5ad(file_path, target_column, filter_values)
adata = perform_umap(adata)
plot_umap(adata, color_column=target_column)
```

In the resulting UMAP, both groups show two predominantly distinct clusters, with evident separation yet a small degree of overlap

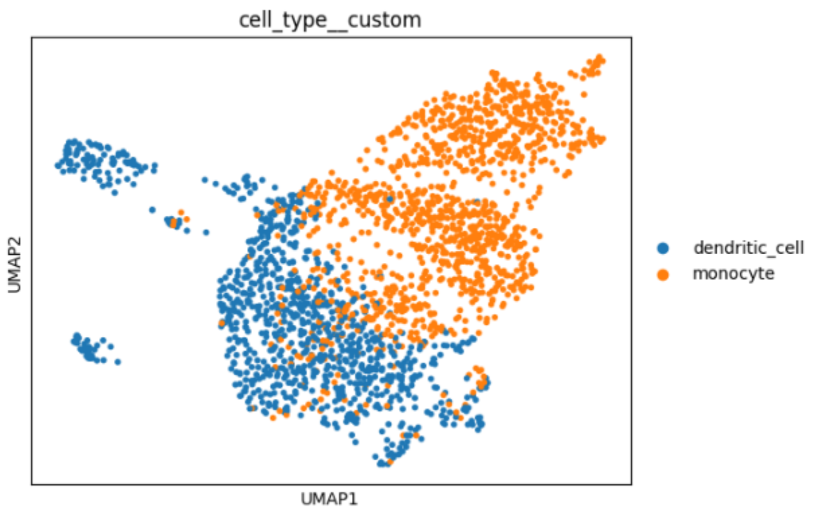

gSELECT Analysis

Optional: Creating a final Hold-out Test Set

As an optional step, a final test set can be created to prevent information leakage and ensure an unbiased evaluation of model performance.

This step is not required for all analyses but can be helpful to separate the model development (training) from the final evaluation.

By removing the test set *before* calculating mutual information, selecting genes, or training the model, you avoid information leakage and circular analysis. This allows an independent and unbiased evaluation of the final model.

The final test set is kept separate and used **only** once at the end to evaluate the generalization performance of the selected gene set or trained classifier.

To create a hold-out test set:

- The data is first transposed so that samples are rows and genes are columns.
- 80% of the samples are randomly selected as the training set. (you can change this by changing the variable **(frac=0.8))**
- The remaining 20% of the samples are set aside as a final test set.
- Both sets are then transposed back to the original format (samples × genes).

```
data_total = data.transpose()

training_data = data_total.sample(frac=0.8)

test_data = data_total.drop(training_data.index)

training_data = training_data.transpose()

test_data = test_data.transpose()
```

When not using a final hold-out test set

By calculating mutual information and training the model on the **entire dataset**, you maximize the number of available samples for both feature selection and learning. This has two main advantages:

- **Higher statistical power**: With more samples, mutual information scores are more robust, especially for small effect sizes or noisy data.
- **More stable gene selection and model training**: The classifier has access to all available expression profiles, which can improve model performance metrics such as accuracy or stability of top-ranked genes.

However, it is important to note that this approach only provides an **internal estimate** of performance (e.g., through cross-validation or multiple sweeps), and **not** an external validation. As a result, the reported accuracy might be **optimistically biased**, particularly if the number of sweeps or folds is small or if the signal is weak.

Mutual Information

The first step is calculating the mutual information. An additional, optional feature of gSELECT is the possibility to exclude certain genes from the analysis by entering gene names in the exclusion_list. When the exclusion_list is empty, all genes are considered.

If you are using the Split function, you can run this code to calculate the mutual information

```
exclusion_list = [
    "example_gene"
]
mutual_info = gsfs.compute_mutual_information(genes, training_data,
output_folder=output_path,exclusion_list=exclusion_list)
```

If you want to calculate mutual information on the whole set, you can use the code like this: Instead of test_data use data.

```
exclusion_list = [
    "example_gene"
]
mutual_info = gsfs.compute_mutual_information(genes, data,
output_folder=output_path,exclusion_list=exclusion_list)
```

If you want to exclude genes – you can just fill out the exclusion list and calculate mutual information:

```
exclusion_list = [
    "LYST","RIPOR2","TCF7L2","ADAM28","VCAN","PRKCB"
]
mutual_info = gsfs.compute_mutual_information(genes, data,
output_folder=output_path,exclusion_list=exclusion_list)
```

The output

```
Full mutual information saved to output\mutual_information.csv
```

Indicates that the mutual information was successfully calculated and saved. The list of genes is saved as mutual_information.csv in output. The csv file contains a list of all genes, ranked according to mutual information.

ATTENTION: Mutual information will only be calculated when there is no mutual_information.csv yet. If the analysis is performed for the second time, the mutual information will not be calculated again if the mutual_information.csv already exists in the output directory.

Table below shows the top 10 genes for both comparisons. Although both lists are quite similar and the top 10 genes mainly are ranked at the top there are slight differences in mutual information values and rankings.

Mutal Information on Full Data		Mutal Information on Training Data	
**gene_name**	**mutual information**	**gene_name**	**mutual information**
**LYST**	0.255319507099773	**LYST**	0.24401017355587934
**TCF7L2**	0.24252696191432055	**TCF7L2**	0.23584455901832374
**ADAM28**	0.24016590615093392	**PRKCB**	0.23571996727517897
**RIPOR2**	0.2381116299013658	**VCAN**	0.23270768282894005
**VCAN**	0.23306675459745768	**CCSER1**	0.23212783177318952
**CCSER1**	0.2307069499981879	**RIPOR2**	0.2320222243479464
**PRKCB**	0.22184433536989284	**ADAM28**	0.226043445402618
**SLC11A1**	0.2138660433144842	**SLC11A1**	0.22303440849657347
**LYN**	0.20023686626627313	**CTSS**	0.19500734135855904
**AC120193.1**	0.18999917586564474	**UTRN**	0.19393815499077238

In this tutorial, we use the full dataset but for calculating the accuracy of all genes and the top10 MI-genes we will show and compare the plots for both – full data and split data.

Analysis Option 1: Classification and Predictive Value of ALL genes

As a first step, we recommend analysing all genes, as this will show the maximal possible predictive power. Additionally, the balanced accuracy of analysing all genes indicates whether further analyses are likely to yield results. (A balanced accuracy of about 50%, which is similar to guessing would indicate that analysing the groups might not be advisable.)

```
results = gsc.run_all_genes(
    data,
    mutual_info,
    number_sweeps=10,
    max_iterations=500
)
gsv.plot_all_genes(
    results,
    output_folder=output_path,
    dpi=600,
    csv_name="all_genes_rankings.csv",
    save_csv=True,
    save_png=True
)
```

Short explanation to number_sweeps and max_iterations

The number_sweeps parameter defines how often the classification is repeated with different internal random seeds and training configurations. Each sweep includes:

- a new random initialization of the model,
- a new training-validation split,
- and a full training run with the current gene selection.

Running multiple sweeps increases the **stability** and **reliability** of the accuracy estimates by averaging across different runs. This helps to avoid misleading results due to a single lucky or unlucky model initialization.

However, this also means:

- The **runtime increases linearly** with the number of sweeps.
- For example, number_sweeps=10 will take roughly **10× longer** than number_sweeps=1, assuming constant settings elsewhere.

For exploratory analyses, a lower number (e.g., number_sweeps=3) is often sufficient. For final evaluations, increasing the number (e.g., to 10 or more) can improve result robustness.

The **parameter max_iterations** defines how many optimisation steps each multilayer perceptron (MLP) classifier is allowed to perform during training. A higher number of iterations can improve model convergence, especially for complex datasets or small learning rates. This ensures that the model has enough opportunity to converge, even when using all genes or complex gene subsets. If the number of iterations is too low, the model might not reach its optimal performance, especially on noisy or high-dimensional data.

The max_iterations parameter affects how long the training step takes. Each sweep trains a multilayer perceptron (MLP) using a numerical optimization procedure (e.g., stochastic gradient descent or a variant thereof). Setting max_iterations=500 allows up to 500 optimization steps per sweep if not already converged before.

- A **higher value** may final accuracy, but also **increases runtime**, especially when used in combination with a large number of sweeps or many input features.
- A **lower value** speeds up the process but might result in **premature stopping** if the model has not yet converged.

Note: In most cases, 500 iterations are sufficient. If a convergence warning appears during training, increasing this value might help.

For most datasets, max_iterations=500 strikes a good balance between performance and runtime. If runtime becomes a limiting factor (e.g., in exhaustive searches), reducing this value may be considered, although it could reduce predictive accuracy or the corresponding best value comes with a higher uncertainty (standard deviation of the model accuracies over all sweeps).

Visualisation

The resulting plots are shown in the Notebook and saved in the output directory.

On the full set, the balanced accuracy of all genes is around 95% with about 19 misclassified samples. The balanced accuracy indicates that the prediction of the group was to 95% correct when using all available genes, which equals a high predictive power for this gene selection. Additionally, out of all the cells (around 2000 cells per cell type) in the single cell data (here referred to as samples), only 19 were misclassified as belonging to the other group.

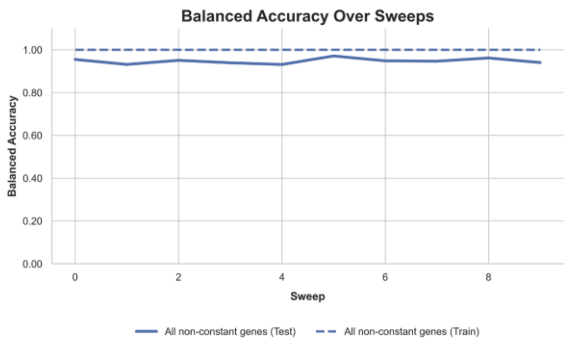

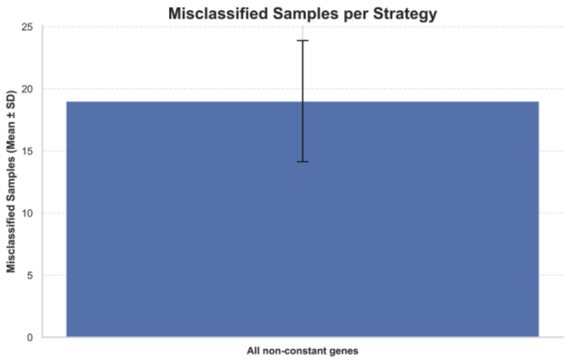

Mean balanced accuracy: 0.9458290952115844

Std deviation: 0.011183965170036312

For split data we get similar results in this use case:

Mean balanced accuracy: 0.9475330306881184

Std deviation: 0.01211766506918404

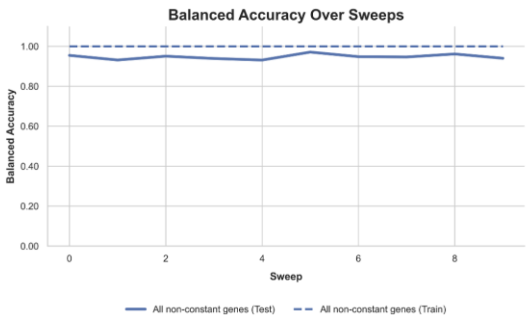

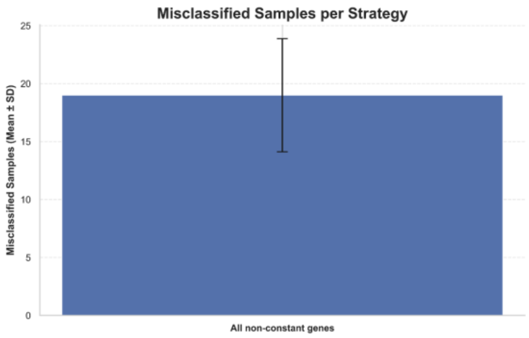

Analysis Option 2: Classification and Predictive Power of SELECTED genes

Since the analysis of all genes indicates that the data is suitable for further analysis, we now analyse the predictive power of a smaller number of genes.

This performs two analyses: One with the 10 top-ranked genes according to mutual information (the MI genes, shown as blue lines) and one with an equal number of random genes (here 10 random genes, shown as green lines).

```
results = gsc.run_selected_genes(
    data,
    mutual_info,
    number_sweeps=10,
    top_n_genes=10,
    include_random=True,
    max_iterations=500
)
gsv.plot_results(
    results,
    output_folder=output_path,
    dpi=600,
    save_csv=True,
    csv_name="selected_genes_results.csv",
    save_png=True
)
```

Here we have the case that max_iterations of 500 were not enough. There is a warning in the system – for better results you can choose a higher max_iterations value.

```
2025-07-20 13:45:02,199 - INFO - Running experiment with       gene_name  index feature  mutual information
14307      LYST          16323            0.244010
20705    TCF7L2          23086            0.235845
17448     PRKCB          19676            0.235720
22228      VCAN          24697            0.232708
7736     CCSER1           9202            0.232128
18333    RIPOR2          20606            0.232022
3855     ADAM28           4778            0.226043
19312   SLC11A1          21622            0.223034
8832       CTSS          10347            0.195007
22192      UTRN          24660            0.193938...
2025-07-20 13:45:02,200 - INFO - Running sweep 1 of 10...
2025-07-20 13:45:02,200 - INFO - Peak memory usage: 2285728.00 MB
/Users/aylin/anaconda3/lib/python3.10/site-packages/sklearn/neural_network/_multilayer_perceptron.py:780: Convergen
ceWarning: Stochastic Optimizer: Maximum iterations (500) reached and the optimization hasn't converged yet.
  warnings.warn(
2025-07-20 13:45:03,616 - INFO - Running sweep 2 of 10...
```

The results for the full run with top 10 MI-Gens are again visualized. The blue line is for the selected Genes in test, the dotted blue line is for training - the green lines are for randomly selected genes.

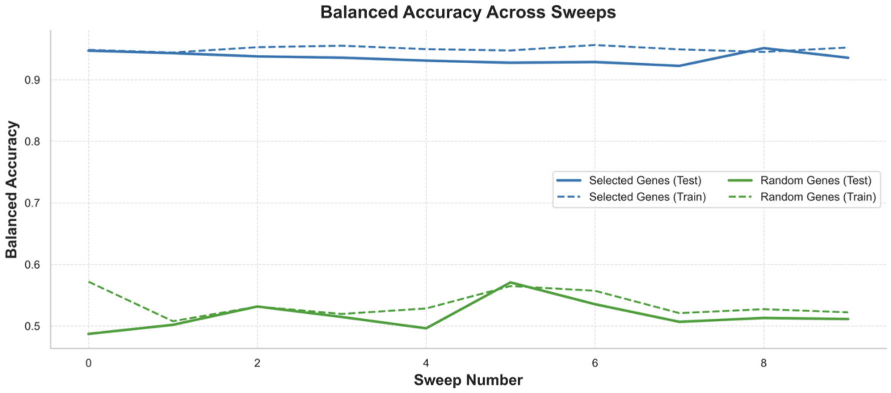

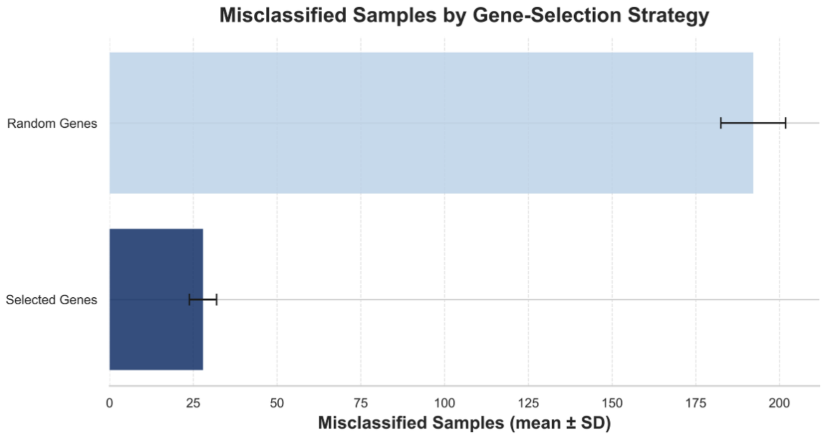

For each sweep you can check the accuracies in the selected_genes_results.csv

For the Split Analysis we again have very similar results

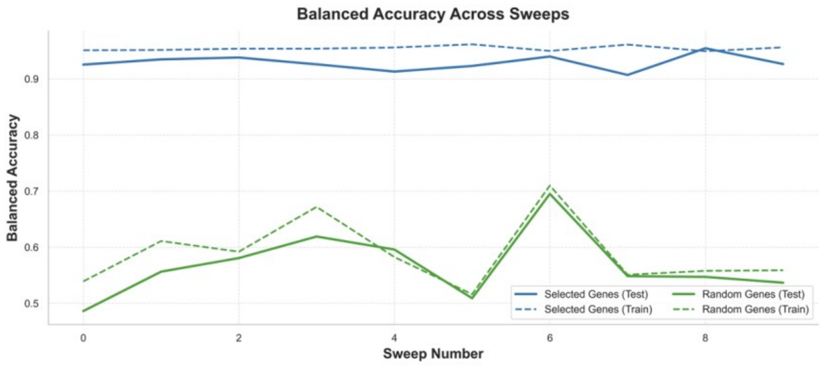

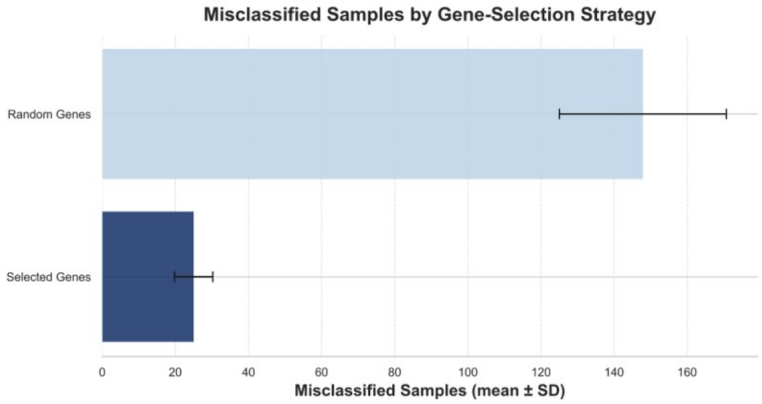

Analysis Option 3: Finding the Optimal Number of Genes

Analysing the 10 top-ranked genes already indicated a high predictive power for five MI genes. To find the optimal number of MI genes, different numbers of genes can be tested

```
gene_selection = [1, 2, 3, 4, 5, 6, 7, 8, 9, 10, 15, 20]
results = gsc.run_multiple_gene_selections(
    data,
    mutual_info,
    number_sweeps=10,
    gene_selection=gene_selection,
    max_iterations=500
)
gsv.plot_multiple_gene_selections(
    results,
    output_folder=output_path,
    save_csv=True,
)
```

Here, we tested the top-ranked MI gene alone, the 2 top-ranked MI genes, …., the 10 top-ranked MI genes, the 15 top-ranked MI genes and the 20 top-ranked MI genes.

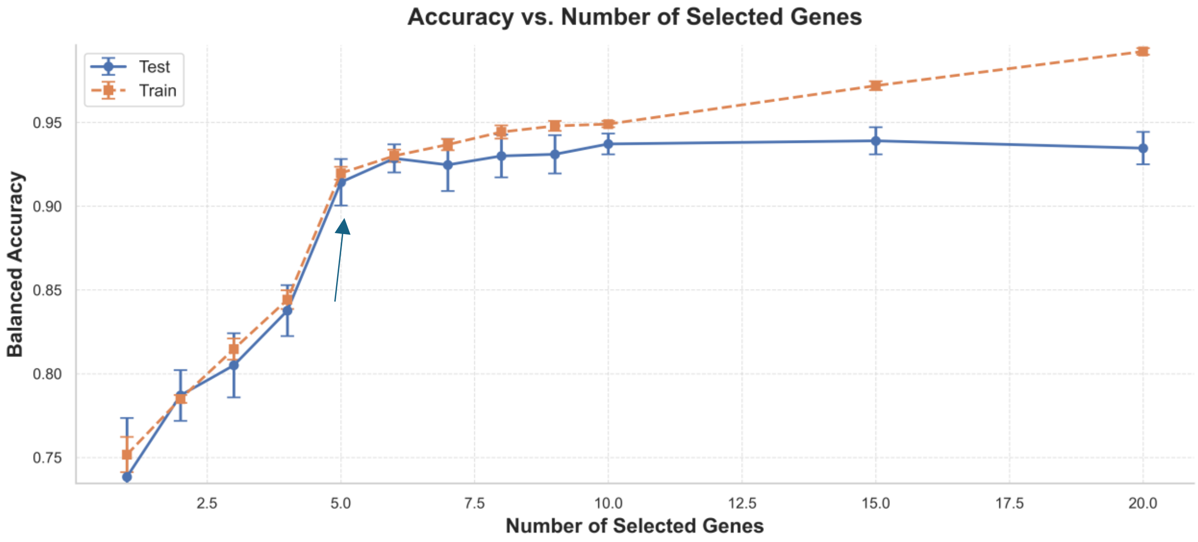

The analysis results indicate that the 5 top-ranked MI genes are the smallest number of genes to achieve an accuracy of over 90%.

For split data we have a very similar results – but here the top 6 MI-Genes seem to have the best accuracy.

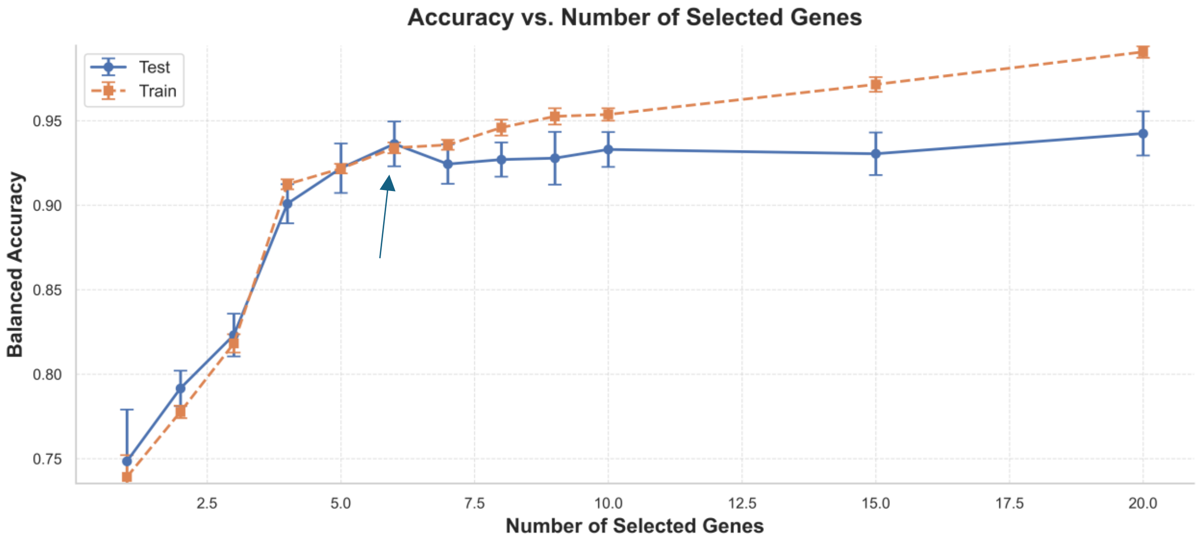

Mutal Information on Full Data			Mutal Information on Training Data		
gene_name		mutual information	gene_name		mutual information
LYST	1	0.255319507099773	LYST	1	0.24401017355587934
TCF7L2	2	0.24252696191432055	TCF7L2	2	0.23584455901832374
ADAM28	x	0.24016590615093392	PRKCB	x	0.23571996727517897
RIPOR2	4	0.2381116299013658	VCAN	4	0.23270768282894005
VCAN	5	0.23306675459745768	CCSER1	5	0.23212783177318952
CCSER1	6	0.2307069499981879	RIPOR2	6	0.2320222243479464

If we compare the table, we can see that both have 5 of 6 genes in common when comparing the top 6 MI-Genes.

Analysis Option 4: Calculating the Predictive Power of CUSTOM genes

The predictive power of any gene(s) of interest from literature, previous knowledge, … can be calculated using the gene_list:

```
gene_list = ["SLC11A1", "FCN1", "MRC1", "LYN", "HLA-DQA1", "RIPOR2"]
results = gsc.run_with_custom_gene_set(
    data,
    gene_list,
    mutual_info,
    number_sweeps=5,
    max_iterations=500
)
gsv.plot_results(
    results,
    output_folder=output_path,
    dpi=600,
    save_csv=True,
    csv_name="custom_gene_set_results.csv",
    save_png=True
)
```

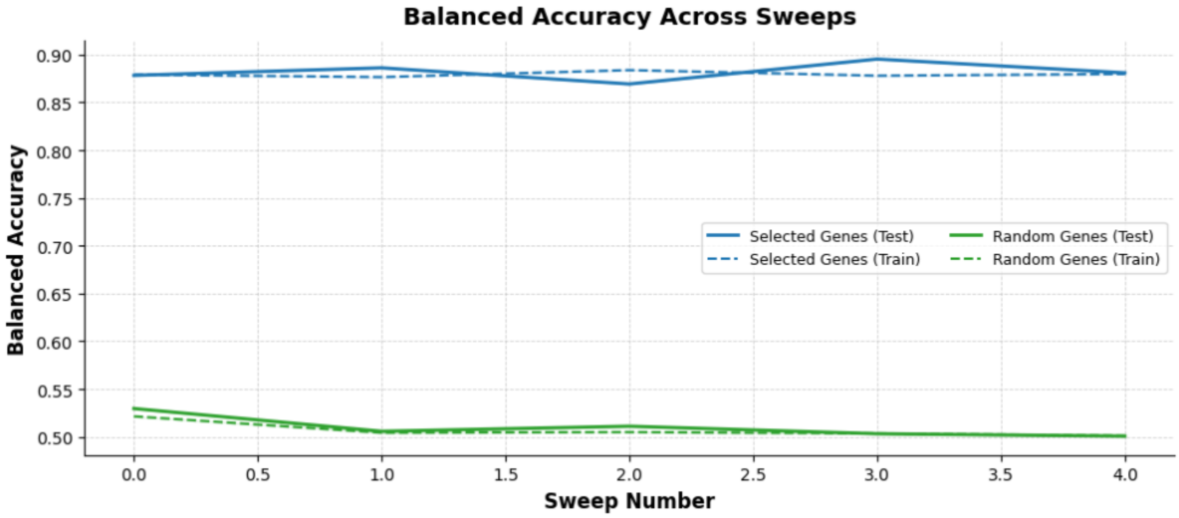

You will get the results for your selected genes in comparison to random genes. Training and test data will be visualized, and all results will be saved in a csv file.

Analysis Option 5: Explorative Analysis of Predictive Power

With gSELECT it is also possible to analyse the predictive power different gene combinations. With the run_explorative_gene_selections() function, gSELECT will test all possible different combinations for the respective number of genes.

As described in the methods section, the number of possible subsets follows $2^n - 1$.

While analysing 3 genes results in 7 combinations (A, B, C, AB, AC, BC, and ABC), analysing 5 genes will already result in 31 combinations, and analysing 10 genes already results in more than 1000 possible combinations, while analysing 20 genes results in more than a million of possible combinations.

5 genes → 31 combinations

10 genes → 1,023 combinations

15 genes → 32,767 combinations

20 genes → 1,048,575 combinations

25 genes → 33,554,431 combinations

30 genes → 1,073,741,823 combinations

….

To reduce computational load when working with large datasets or high numbers of candidate genes, the run_explorative_gene_selections() function supports both multithreading and a greedy fallback mode. By default, all available threads are used. However, setting num_threads to a lower value (e.g., 2) allows the analysis to proceed in the background while keeping system resources available for other tasks.

When use_greedy_if_large=True, the function automatically switches from exhaustive evaluation to a greedy search strategy once the number of top-ranked genes exceeds the greedy_threshold (default: 10). This avoids the exponential growth of subset combinations, which would otherwise render the exhaustive approach infeasible. For further details on the greedy mode and its implementation logic, please refer to the *Methods* section of the manuscript.

If use_greedy_if_large=False, gSELECT will always perform an exhaustive search, regardless of the number of genes. Similarly, decreasing greedy_threshold forces earlier switching to the greedy strategy; increasing it delays the switch. To enforce full exhaustive evaluation at all times, set use_greedy_if_large=False.

It is also possible to run the explorative function with custom genes.

```
gene_list = ["SLC11A1", "LYN", "PRKC8", "HLA-DQA1", "RIPOR2"]
results = gsc.run_explorative_gene_selections_with_custom_set(
    data,
    gene_list,
    mutual_info,
    number_sweeps=3,
    num_threads=12,
    max_iterations=500
)
```

Here we selected top 15 ranked MI-Genes. With exhaustive mode we would have 32,767 combinations. As our greedy threshold was set on 10 this means that the calculation will be made in greedy mode.

```
results = gsc.run_explorative_gene_selections(
    data,
    mutual_info,
    number_sweeps=3,
    top_n_genes=15,
    num_threads=12,
    max_iterations=500,
    use_greedy_if_large=True,
    greedy_threshold=10
)
gsv.plot_explorative_gene_selections(
    results,
    top_n=10,
    output_folder=output_path,
    cmap_name="Blues",
    show_delta=True,
    annotate=True,
    dpi=600,
    csv_name="explorative_gene_subset_rankings.csv",
    save_csv=True,
    save_png=True,
)
```

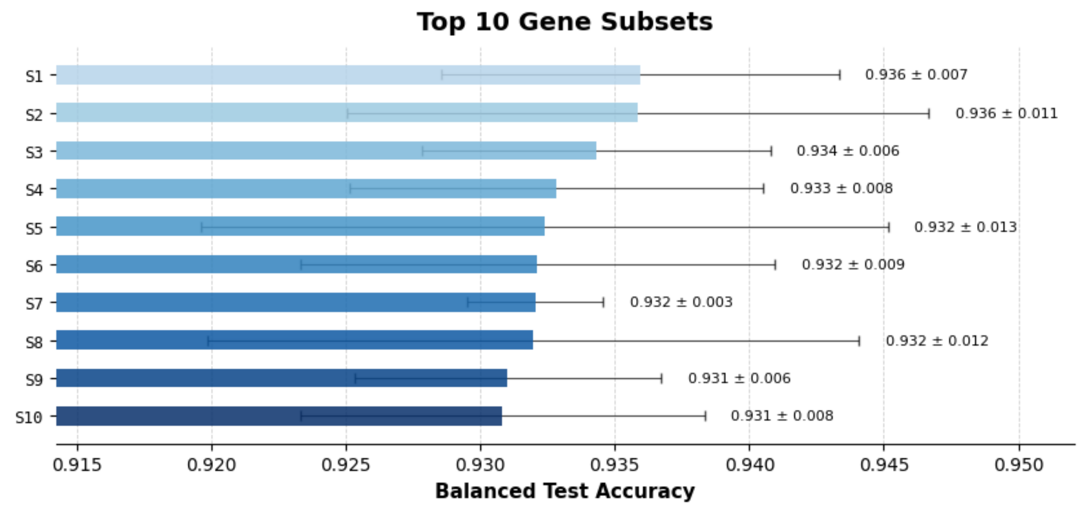


```
Gene Subset Legend:
S1: LYST, VCAN, TCF7L2, UTRN, ADAM28, HLA-DQA1, PRKCB, RIPOR2, LYN
S2: LYST, VCAN, TCF7L2, UTRN, ADAM28, HLA-DQA1, PRKCB, RIPOR2, CCSER1
S3: LYST, VCAN, TCF7L2, UTRN, ADAM28, HLA-DQA1, PRKCB, RIPOR2, AFF3
S4: LYST, VCAN, TCF7L2, UTRN, ADAM28, HLA-DQA1, PRKCB, RIPOR2
S5: LYST, VCAN, TCF7L2, UTRN, ADAM28, HLA-DQA1, PRKCB, AFF3
S6: LYST, VCAN, TCF7L2, UTRN, ADAM28, HLA-DQA1, PRKCB, RIPOR2, AC120193.1
S7: LYST, VCAN, TCF7L2, UTRN, ADAM28, HLA-DQA1, PRKCB, RIPOR2, CTSS
S8: LYST, VCAN, TCF7L2, UTRN, ADAM28, HLA-DQA1, PRKCB, RIPOR2, LYN, CCSER1
S9: LYST, VCAN, TCF7L2, UTRN, ADAM28, HLA-DQA1, PRKCB, RIPOR2, LYN, AC120193.1
S10: LYST, VCAN, TCF7L2, UTRN, ADAM28, HLA-DQA1, PRKCB
```

The plot displays the 10 best-performing gene subsets in terms of balanced test accuracy, based on greedy subset evaluation (see Methods). All top subsets include the same 7 core genes – *LYST*, *VCAN*, *TCF7L2*, *UTRN*, *ADAM28*, *HLA-DQA1*, and *PRKCB* – suggesting that these genes are particularly informative for distinguishing between the two groups.

While the predictive performance is compelling, further validation would be required to assess biological relevance. In particular, follow-up analyses, literature curation, or pathway enrichment would be advisable and, ideally, experimental validation should be considered to confirm the functional role of these candidate markers in a real-world application.

Final Notes

As stated above, this tutorial is based on the July 2025 version of gSELECT.

The most recent version of the code is always available on GitHub, including a detailed README and a ready-to-use Jupyter Notebook.

We will continue to update the GitHub repository as new features are added, or improvements are made. If you are working with large datasets, please refer to the *Methods* section of the manuscript for technical details (e.g. on greedy mode and resource management).

For feedback, questions, or contributions, feel free to open an issue or contact us directly.

Happy gene prioritizing!

References

1 Emont, M. P. *et al.* A single-cell atlas of human and mouse white adipose tissue. *Nature* **603**, 926-933 (2022). https://doi.org:10.1038/s41586-022-04518-2
